# Proteostasis regulators modulate proteasomal activity and gene expression to attenuate multiple phenotypes in Fabry disease

**DOI:** 10.1042/BCJ20190513

**Published:** 2020-01-30

**Authors:** Susanne Seemann, Mathias Ernst, Chiara Cimmaruta, Stephan Struckmann, Claudia Cozma, Dirk Koczan, Anne-Marie Knospe, Linda Rebecca Haake, Valentina Citro, Anja U. Bräuer, Giuseppina Andreotti, Maria Vittoria Cubellis, Georg Fuellen, Andreas Hermann, Anne-Katrin Giese, Arndt Rolfs, Jan Lukas

**Affiliations:** 1 Translational Neurodegeneration Section “Albrecht-Kossel”, Department of Neurology, University Medical Center Rostock, University of Rostock, 18147 Rostock, Germany; 2 Institute for Biostatistics and Informatics in Medicine and Ageing Research, University Medical Center Rostock, 18057 Rostock, Germany; 3 Institute of Biomolecular Chemistry, CNR, 80078 Pozzuoli, Italy; 4 Department of Biology, University Federico II, 80126 Naples, Italy; 5 Centogene AG, Rostock, Germany; 6 Institute of Immunology, University Medical Center Rostock, 18057 Rostock, Germany; 7 Institute of Anatomy, University Medical Center Rostock, 18057 Rostock, Germany; 8 Research Group Anatomy, School of Medicine and Health Sciences, Carl von Ossietzky University Oldenburg, 26129 Oldenburg, Germany; 9 Research Center for Neurosensory Science, Carl von Ossietzky University Oldenburg, Oldenburg, Germany; 10 Center for Transdisciplinary Neurosciences Rostock (CTNR), University Medical Center Rostock, University of Rostock, 18147 Rostock, Germany; 11 German Center for Neurodegenerative Diseases (DZNE) Rostock/Greifswald, 18147 Rostock, Germany; 12 Department of Neurology, Massachusetts General Hospital, Harvard Medical School, Boston, MA, U.S.A.; 13 Program in Medical and Population Genetics, Broad Institute of MIT and Harvard, Cambridge, MA, U.S.A.; 14 University Medical Center Rostock, University of Rostock, 18057 Rostock, Germany

**Keywords:** globotriaosylsphingosine, lysosomal enzyme, proteasome inhibitor, protein misfolding, transcriptomics

## Abstract

The lysosomal storage disorder Fabry disease is characterized by a deficiency of the lysosomal enzyme α-Galactosidase A. The observation that missense variants in the encoding *GLA* gene often lead to structural destabilization, endoplasmic reticulum retention and proteasomal degradation of the misfolded, but otherwise catalytically functional enzyme has resulted in the exploration of alternative therapeutic approaches. In this context, we have investigated proteostasis regulators (PRs) for their potential to increase cellular enzyme activity, and to reduce the disease-specific accumulation of the biomarker globotriaosylsphingosine in patient-derived cell culture. The PRs also acted synergistically with the clinically approved 1-deoxygalactonojirimycine, demonstrating the potential of combination treatment in a therapeutic application. Extensive characterization of the effective PRs revealed inhibition of the proteasome and elevation of *GLA* gene expression as paramount effects. Further analysis of transcriptional patterns of the PRs exposed a variety of genes involved in proteostasis as potential modulators. We propose that addressing proteostasis is an effective approach to discover new therapeutic targets for diseases involving folding and trafficking-deficient protein mutants.

## Introduction

Fabry disease (FD, OMIM 301500) is one of more than 40 lysosomal storage diseases (LSD) [1]. According to recent data, FD is possibly the most common LSD with an incidence found to be 1 : 1 250 to 1 : 37 800 [2] depending on the severity of symptoms. FD is caused by mutations in the X-linked gene encoding the lysosomal enzyme α-Galactosidase A (gene symbol: *GLA*, protein: α-Gal A) leading to absent or diminished activity of the enzyme [3]. Many missense variants of the *GLA* gene lead to impaired protein processing within the endoplasmic reticulum (ER) and an altered conformation that results in ER retention and premature ER-associated degradation (ERAD) [4]. Deficient activity of α-Gal A, in turn, causes progressive accumulation of Globotriaosylceramide (Gb3) or its metabolite Globotriaosylsphingosine (lyso-Gb3) [3]. The measurement of lyso-Gb3 in plasma and whole blood is considered of diagnostic as well as of prognostic value for the assessment of the clinical outcome of *GLA* mutations [5–7].

The current therapeutic strategy involves enzyme replacement therapy (ERT) with intravenous infusions of α-Gal A. Different formulations are available from different sources and manufacturers. The benefit of ERT may be impaired by many limitations including an insufficient penetration in key tissues [8], an immune response leading to the formation of IgG antibodies that may hamper the effectiveness of the treatment [9], the patient burden of a life-long inconvenient intravenous therapy and high cost. The clinical approval of the orally available pharmacological chaperone (PC) therapy using the active-site specific sugar mimetic 1-deoxygalactonojirimycine (DGJ) represents a recent therapeutic advance for a fraction of FD patients [10]. These patients harbor missense variants, which are associated with a destabilized though catalytically active α-Gal A enzyme. The effectiveness of DGJ is based on its direct binding to the immature α-Gal A within the ER. The variant enzyme then attains a thermodynamically favored folding state, which leads to a reduced elimination by ERAD and, consequently, to a shift to a greater enzyme fraction being further transported along the secretory route to the lysosomes raising the level of available, active α-Gal A [11].

New therapeutic approaches include the use of small molecules, which have the capacity to modify proteostasis, including protein synthesis, folding and degradation. They either increase the folding capacity of the ER or enhance the degradation of misfolded proteins in order to resolve the protein overload [12]. Therefore, they are referred to as proteostasis regulators (PRs). Many of these have been proposed as potential candidate drugs in protein misfolding and aggregation diseases (e.g. Cystic Fibrosis, Alzheimer's disease, retinitis pigmentosa) [12–15] and particularly LSD [16–20]. Either the protein variants that have resulted in the diseases are to be removed from the system, since toxic gain-of-function variants have developed, or the functionality of the protein must be restored by preventing degradation, i.e. a rescue of loss-of-function. Depending on the goal to be pursued, the properties of an effective drug are determined. Proteostasis is maintained by a highly conserved cellular machinery that regulates protein folding in general, and specifically, the protein misfolding-induced unfolded protein response (UPR) which activates the ERAD [21–23]. Signal integration within the proteostasis network is associated with extensive gene regulation [24,25] and leads to cell type-specific transcriptional patterns in response to stress in order to restore homeostasis [26]. The relation between protein folding diseases and the expression of proteostasis genes is being examined by a growing research community [16,17,21,23,27–33]. Additionally, the role of gene expression regulation, particularly of genes involved in proteostasis processes, has been proposed to be part of the work mechanism of PRs besides their primary biochemical function [16,17,21,27–30,33]. This gene regulator function of PRs might have an impact on the rescue of misfolded proteins. First indications for a meaningful use of PRs in FD can be found in earlier studies [34,35].

The aim of this study was to screen for candidates able to increase variant α-Gal A activity in patient-derived fibroblasts harboring the PC amenable variants c.902G>A (p.R301Q) and c.901C>G (p.R301G), respectively, and to provide a profound characterization of the effects on the proteostasis network.

## Materials and methods

### Chemicals

Chemicals were purchased from Sigma–Aldrich (Steinheim, Germany) except for 17-AAG (Abcam, Cambridge, U.K.); Rosiglitazon, Clasto-Lactacystin β-lactone (CLC), Eeyarestatin I (EerI) and Ritonavir (Cayman Chemicals, Ann Arbor, MI, U.S.A.); Pifithrin-μ (Enzo Life Sciences, Lörrach, Germany); Lacidipine (Key Organics, Cornwall, U.K.); MG132 (Merck (Darmstadt, Germany); 15d-PGJ2 (Santa Cruz Biotechnology, Dallas, TX, U.S.A.); Kifunensine and 1-deoxygalactonojirimycine hydrochloride (Toronto Research Chemicals, Toronto, Canada) and Bortezomib (USBiological, Salem, MA, U.S.A.).

### Cell culture

Wild-type (WT) fibroblast cell lines GM01653 (wild-type 1, WT1), GM23249 (WT2), GM23250 (WT3), GM23968 (WT4) from healthy male donors and Fabry fibroblasts hemizygous for the c.901C>G (p.R301G) variant (GM00882, *GLA*^p.R301G/^°) were purchased from Coriell Institute cell repository (Camden, U.S.A.). Male Fabry fibroblasts hemizygous for the c.902G>A (p.R301Q) variant (*GLA*^p.R301Q/^°) were a kind gift of Amicus Therapeutics (Cranbury, NJ, U.S.A.). Both variants were reported to be amenable to PC treatment [5,35]. All lines were sequenced prior to use to verify the genotypes. Fabry disease was excluded for all healthy donors. However, GM23249 carried the intronic haplotype found to be associated with reduced mRNA expression [36]. Fibroblasts were cultured in Dulbecco's modified Eagle medium containing 4.5 g glucose/l (Gibco, Carlsbad, CA, U.S.A.) supplemented with 15% heat-inactivated fetal bovine serum (Gibco) and 1% Penicillin/Streptomycin (Invitrogen, Carlsbad, CA, U.S.A.) at 37°C in 5% CO_2_. Monolayers were passaged with 0.25% Trypsin-EDTA (Fisher Scientific, Schwerte, Germany) when reaching full confluency.

### Drug treatment

The patients’ fibroblasts were seeded one day prior to the treatment to give the cells time to adhere to the surface of the culture vessel. At the beginning of the treatment, the cells typically had ∼80% confluency. The cells were treated with PR, DGJ, and a combination of both. The exact duration of the treatment and the drug concentrations used can be seen in the figures. Typically, the treatment was followed by a 6-h off-treatment period (‘washout’) for enzyme activity measurement, because α-Gal A required this recovery phase for stable assessment after DGJ treatment (Supplementary Figure S1) and a 4 days washout for biomarker measurements as reported before [37]. The PR treatment was also discontinued for the washout period. After the treatment, the cells were processed according to the downstream application as described in the following paragraphs.

### α-Galactosidase A activity assay

After the treatment for the indicated time, the activity assay was run. The cells were harvested with 0.25% Trypsin-EDTA, washed with phosphate-buffered saline (PBS), resuspended in deionized water and lysed during five freezing and thawing cycles. The protein amount in the cell lysates was determined using the BCA protein assay kit (Thermo Scientific, Waltham, MA, U.S.A.). Five micrograms of total protein was used for enzyme activity measurement with the substrate analog 4-methylumbelliferyl-α-d-galactopyranoside. The reaction product 4-methylumbelliferone was recorded at 360 nm excitation and 460 nm emission in a fluorescence plate reader as described earlier [38].

### Lyso-Gb3 determination in patient-derived fibroblasts

We seeded 2 × 10^5^ fibroblast cells and treated them with PRs for the specified period of time. On the day of harvest, the cells were trypsinized then pelleted, washed with PBS, resuspended in 70 µl deionized water and vortexed for 3 min. The cell suspension was lysed during six cycles of freezing in liquid nitrogen and sonication for 5 min. Samples were centrifuged and the supernatant with the protein extract was transferred to a new tube. The protein amount in the cell lysates was determined using the BCA protein assay kit (Thermo Scientific). Sample preparation for lyso-Gb3 determination and the mass spectrometric analysis was performed as described [39]. For each batch of analyses, a calibration curve was added with a concentration range from 0 to 1 000 ng/ml in water. The concentration of the lyso-Gb3 was recorded in ng/mg protein extract.

### Lipid extraction

Lipids were extracted according to Bligh and Dyer [40] with slight modifications. 1 × 10^6^ human fibroblasts were seeded and cultured for the indicated time points with and without treatment. On the day of cell harvest, the cells were pelleted in non-adhesive wall glass tubes (Schott AG, Mainz, Germany) allowing for lipid extraction. A mixture of chloroform, methanol and hydrochloric acid (2 :4 : 0.1) was added to the samples together with 1% butylated hydroxytoluol, to prevent lipid oxidation. In addition, a fluorescent internal standard, TopFluor® Lyso PA was added at a concentration of 1 mg/ml to ensure the reproducibility of the lipid extraction. The compound is a synthetic lipid, and therefore absent from the real samples. The fluorescence was later measured by CAMAG visionCATS at 366 nm. Chloroform was added to the homogenized samples and vortexed three times with a 10 min break in between. Next, water was added to the samples and vortexed three times with a 10 min break in between, followed by 30 min incubation and centrifugation at 1260 ×***g*** for 10 min. A biphasic separation was visible, and the bottom phase containing a mix of chloroform and lipids was transferred into a new non-adhesive wall glass vessel. Finally, the chloroform was evaporated in an N_2_ chamber, fresh chloroform was added, and the bottles were stored at −20°C until use.

### Separation and analysis of Gb3 by high-performance thin-layer chromatography (HPTLC) and Far-Eastern blot

Samples were transferred to the HPTLC with an automatic TLC Sampler 4 (ATS 4) from CAMAG. The stationary phase was 10 × 10 cm silica gel (60 F254 Merck, KGaA, Darmstadt, Germany). For the mobile phase a chloroform (SupraSolv Merck KGaA, Darmstadt, Germany), methanol (LiChroSolv Merck KGaA, Darmstadt, Germany), ammonia 32% (HiPerSolv VWR Chemicals, Radnor, PA, U.S.A.), water (Rotisolv Carl Roth GmbH, Karlsruhe, Germany) solution at a ratio of 161 : 75 :5 : 10 was used. Lipids then developed on the HPTLC plate were sprayed with primuline reagent (Derivatizer, CAMAG) and visualized under ultraviolet light (366 nm, TLC Visualizer, CAMAG).

Far-Eastern blotting was made according to Taki et al. [41], with slight modifications (TLC blot (far-eastern blot)) and its applications. The plate was immersed in a mixture of isopropanol 0.2% CaCl_2_ : methanol (40 : 20 : 7, v/v/v) for 2 s, then covered with an activated polyvinylidene difluoride (PVDF) membrane (0.45 µm GE Healthcare Amersham Hybond, Fisher Scientific, Pittsburgh, PA, U.S.A.) and a glass microfiber filter (APFF, Merck, KGaA, Darmstadt, Germany). The transfer cassette was pressed for 30 s with an iron heated at 180°C, after which the PVDF membrane was separated from the plate and dried.

PVDF membrane was blocked over night at 4°C with 0.1% BSA diluted in PBS followed by antibody incubation with anti-Gb3 (1 : 1000, TCI, amsbio, Mainz, Germany) in 3% BSA/PBS for 2 h at RT. The secondary antibody used was Mouse Ig, HRP-Linked Whole Ab, Sheep (1 : 5000, ECL) conjugated to horseradish peroxidase. After incubation for 1 h at RT, Gb3 was detected using clarity western ECL Substrate (Bio-Rad 1705061 1 : 1) and analyzed by using ImageLab 6.0 software (Bio-Rad Laboratories, Hercules, CA, U.S.A.).

### Synergy analysis

All calculations were performed using R 3.3.0. The synergyfinder [42] tool was used in version 1.3.0 (with minor patches, see https://github.com/struckma/synergyfinder). For calculating the three-dimensional interaction surface over the dose matrix, the package synergyfinder was used as well. For synergy analysis, the enzyme activity after the treatment of *GLA*^p.R301Q/^° was calculated as the percentage of the maximal determined activity. The enzyme activity of the untreated control cells was subtracted from the non-control samples. Synergy values (*excess over bliss*, *eob*) were determined by calculating the difference between the actual enzyme activity after the combined treatment and the expected additive drug effect given by the BLISS independence model [43] and calculated with the following equation: *E*_D_ + *E*_B_ – *E*_D_ * *E*_B_. Here, *E*_D_ represents the enzyme activity after single treatment with DGJ and *E*_B_ describes the effect of the respective Bortezomib (BTZ) concentration. Synergy was called if the achieved enzyme activity after the combined treatment was higher than the expected additive effect. The BLISS synergy scores for each treatment were plotted as a function of the two drug concentrations.

### Western blot analysis

Cultured WT and *GLA*^p.R301Q/^° fibroblasts were pelleted, washed with PBS and resuspended in 45 µl RIPA buffer containing complete protease inhibitor cocktail (Roche Diagnostics, Mannheim, Germany) followed by a 20 min incubation on ice to complete the lysis. After centrifugation of the samples, the supernatants were used for protein measurement using the BCA protein assay kit (Thermo Scientific) according to the specifications by the manufacturer. The PNGase digestion was carried out using the PNGase F kit from New England Biolabs (Ipswich, MA, U.S.A.) according to the manufacturer's specifications. Hundred micrograms of protein per sample were mixed with Laemmli buffer and incubated for 5 min at 95°C. For the separation of the proteins, SDS–PAGE was performed using the precast 4–15% Criterion™ TGX Stain-Free™ Protein Gels (Bio-Rad Laboratories). Proteins were transferred to a nitrocellulose membrane (GE Healthcare, Braunschweig, Germany), using the Trans-Blot® Turbo™ Midi Nitrocellulose Transfer Packs and the Trans-Blot® Turbo^™^ Transfer System (Bio-Rad Laboratories). The membrane was blocked in 5% non-fat dried skim milk solution for 1 h at room temperature and incubated with primary rabbit polyclonal GAPDH antibody (Abcam, Cambridge, U.K.) at a 1 : 10 000 dilution in TBS-Tween 20 supplemented with 3% non-fat dried skim milk at 4°C overnight. Afterwards, the membrane was washed five times with TBS-Tween 20, incubated with mouse monoclonal α-Gal A antibody at a 1 : 500 dilution in TBS-Tween 20 supplemented with 3% non-fat dried skim milk (Abcam, Cambridge, U.K.) for 2 h at room temperature and washed again with TBS-Tween 20. Then, the membrane was treated with 1 : 20 000 diluted secondary goat anti-rabbit antibody (LI-COR Biosciences, Lincoln, NE, U.S.A.) and 1 : 10 000 diluted goat anti-mouse antibody (Rockland Immunochemicals, Limerick, PA, U.S.A.), both diluted in TBS-Tween 20 including 3% non-fat dried skim milk, for 2 h at room temperature protected from light. After a final washing step, the blots were visualized using an Odyssey® Infrared Imager (LI-COR Biosciences). The determination of the protein size and the quantification of the bands were done using the Odyssey Application Software version 1.2.

### Proteasomal activity assay

Specific proteasome inhibition was examined using the Cell-Based Proteasome-Glo™ Assays (Promega, Madison, WI, U.S.A.) according to the manufacturer's protocol. One day before the assay *GLA*^p.R301Q/^° fibroblasts were seeded in 24-well plates (Sarstedt, Nümbrecht, Germany) and cultured overnight. The experiment was initiated by the addition of the compound. The cells were incubated for 2 h. For the luminometric measurement, the cells were harvested by scraping and 20 000 cells were dissolved in 100 µl PBS + compound. An equal volume of Proteasome Proteasome™-Glo Reagent (Promega) specific for chymotrypsin-like activity determination was added [44] and the suspension was incubated for 10 min followed by a measurement with a Lumat 9507 instrument (Berthold Technologies, Bad Wildbad, Germany) with a measurement time of 2 s.

### Quantitative real-time PCR

*GLA*^p.R301Q/^° fibroblasts were treated for 24 h. The cells were harvested and 2 µl of the crude RNA extract was reverse transcribed using the FastLane Cell cDNA kit (Qiagen, Hilden, Germany) according to the manufactureŕs specification. PCR samples were prepared with the FastStart Essential DNA Green Master kit (Roche, Mannheim, Germany) according to the manufacturer's specification. Primer sequences were 5′-TTCAAAAGCCCAATTATACAGAAA-3′ (forward) and 5′-CTGGTCCAGCAACATCAACA-3′ (reverse) for *GLA* and 5′-TGCCCCCGACCGTCTAC-3′ (forward) and 5′-ATGCGGTTCCAGCCTATCTG-3′ (reverse) for *G6PD*, respectively. PCR was carried out with the LightCycler® Nano (Roche, Mannheim, Germany) in combination with the LightCycler® Nano SW 1.1 software. Changes of mRNA amounts were calculated using the efficiency corrected relative quantification model [45].

### Microarray analysis

1.2 × 10^6^ *GLA*^p.R301Q/^° fibroblasts were seeded in 10 cm dishes and treated with the indicated compound for 24 h (see Supplementary Table S1). Each condition was performed in quadruplicate. The cells were homogenized in Buffer RLT Plus of the RNeasy Plus Mini Kit (Qiagen, Hilden, Germany). Total RNA was purified utilizing the RNeasy Plus Mini Kit according to the manufacturer's specification. Microarray-based gene expression analysis was performed with GeneChip® Human Transcriptome Arrays 2.0 (Affymetrix, St. Clara, CA, U.S.A.). The RNA samples were amplified and labeled using the GeneChip® WT PLUS Reagent Kit (Thermo Fisher Scientific) according to the manufacturer's instructions. For the overnight hybridization, the GeneChip® Hybridization Oven (Affymetrix) was utilized and the visualization was done using the GeneChip Scanner 3000/7G (Affymetrix). The original data were subjected to quality control using the Expression Console Software (Version 1.4.1.46, Affymetrix). Background correction and normalization were performed using the Robust Multichip Average procedure [46]. Differentially expressed genes were identified by moderated *t-*test with Benjamini–Hochberg *P*-value adjustment. An absolute fold change ≥ 1.5 coupled with an adjusted *P*-value ≤ 0.05 were considered significant.

### Wikipathways analysis

Transcriptional signatures of MG132, BTZ, CLC and EerI were analyzed for pathway annotation. WikiPathways analysis was performed using the R software version 3.2.3 utilizing the Bioconductor package org.Hs.eg.db [47].

### Extended proteostasis gene signature

We compiled an extended ERAD/proteasome gene signature. We obtained a list of known ERAD/proteostasis genes [23]. We then constructed a gene-centric interaction network based on the STRING 10.0 interactome database. Querying this network with the candidate genes yielded multiple network subcomponents. We identified genes that could parsimoniously bridge these subcomponents, yielding a set of additional ERAD/proteostasis candidate genes. We subjected these candidate genes to a manual review to positively establish their involvement in ERAD/proteostasis, assessing whether they were annotated with GO terms related to ER protein folding, UPR or ERAD. Finally, the candidate gene list was augmented by adding ubiquitin associated E1/E2 and proteasome-associated genes utilizing the respective HGNC gene lists. Three hundred and fifty-seven genes were identified that are in the described context with proteostasis.

### Statistical analysis

Statistical data analysis was carried out using GraphPad Prism 5 (GraphPad Software Inc., U.S.A.), Excel software (Microsoft, U.S.A.) and R software (R Foundation). Experimental data are given as mean ± SD. Differences between treatment groups were analyzed using One-way ANOVA with post-hoc Dunnett test as indicated (*, **, ***, ****P-values of 0.05, 0.01, 0.001 and <0.0001). The number of independent experiments is indicated in the figure legends.

## Results

### Abnormal changes in Fabry patient-derived fibroblasts

**Figure 1.**
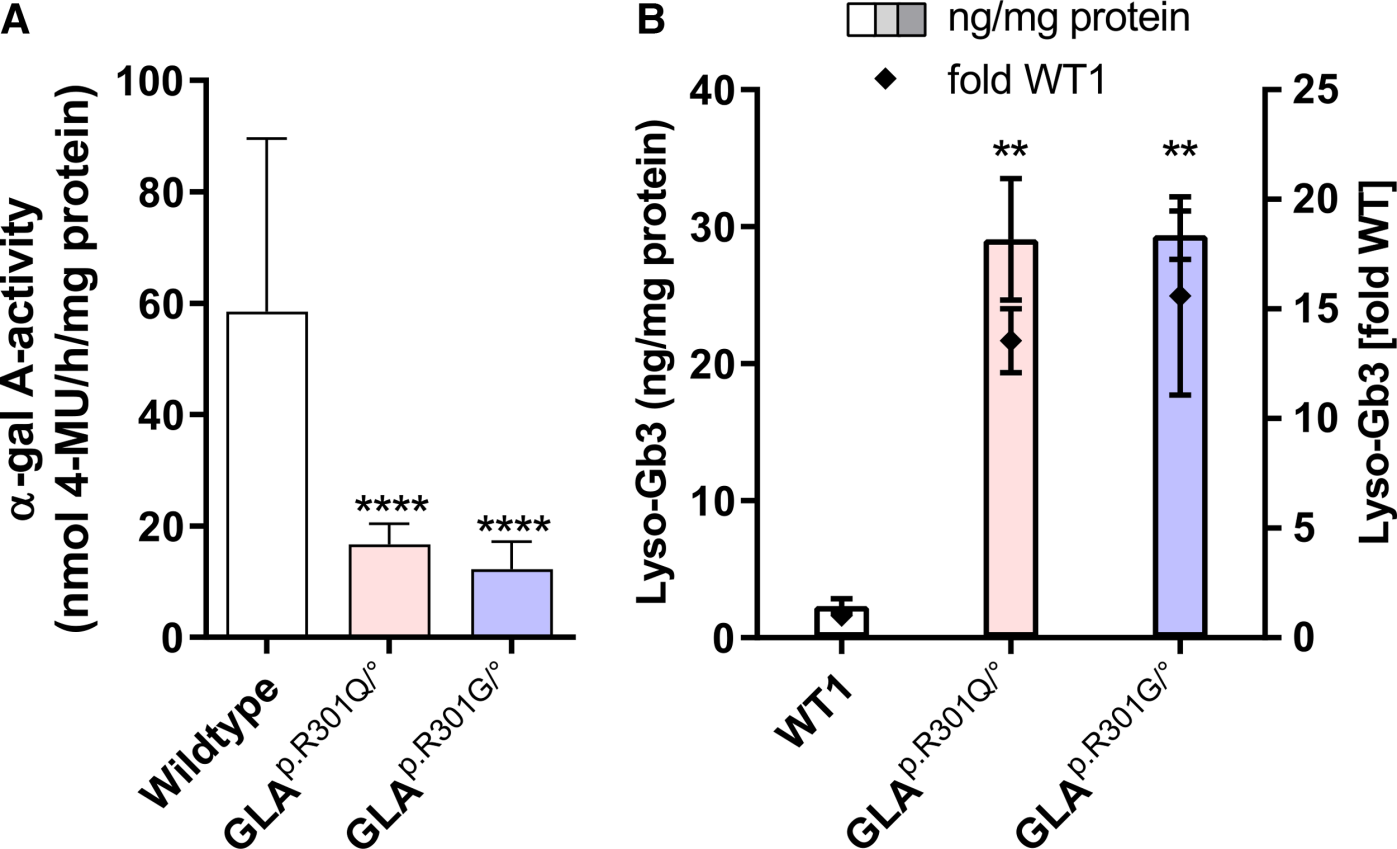
Determination of the pathophysiology of Fabry patient-derived fibroblasts. (**A**) WT and FD fibroblasts were cultured for 5 days. Cell homogenates were used to determine enzyme activity with 4-methylumbelliferyl-α-d-galactopyranoside as a substrate. Shown is the substrate turnover per hour per mg protein as the mean. The wild-type control bar represents the activity obtained from four different fibroblast cell lines. The data are reported as mean ± SD. Each individual wild-type cell line was tested at least four times. The FD cell activity was obtained from 16 independent experiments. (**B**) WT1, *GLA*^p.R301Q/^° and *GLA*^p.R301G/^° fibroblasts were cultured for 7 days followed by lyso-Gb3 determination, which was normalized to the protein amount in each sample (left *y*-axis). The data are reported as mean ± SD ng lyso-Gb3/mg protein (left *y*-axis) and fold change (right *y*-axis) from three independent experiments. Statistics: Differences between the groups were analyzed using One-way ANOVA with post-hoc Dunnett test (*, **, ***, ****P-values of 0.05, 0.01, 0.001 and <0.0001). For (**B**), the statistical evaluation refers to the left *y*-axis.

Enzymatic α-Gal A activity in patient-derived fibroblasts from adult male hemizygous Fabry patients harboring the variants p.R301Q (*GLA*^p.R301Q/^°) and p.R301G (*GLA*^p.R301G/^°) was initially compared with four fibroblast cell lines from healthy age and sex-matched donors (WT 1–4) (Figure 1A). The values for the enzyme activity in *GLA*^p.R301Q/^° (16.70 ± 3.70 nmol 4-MU/mg protein/h) and *GLA*^p.R301G/^° (12.28 (±4.93) nmol 4-MU/mg protein/h) fibroblasts, respectively, were reduced compared with the WT cells (58.5 (±31.1) nmol 4-MU/mg protein/h). Both common FD storage products lyso-Gb3 and Gb3 were also analyzed. Lyso-Gb3 showed a significant increase in both patient cell lines compared with WT1 cells (Figure 1B). The Gb3 level of *GLA*^p.R301Q/^° fibroblasts was also found to be elevated (Supplementary Figure S2).

### Proteostasis regulators as effective α-Gal A enhancers

**Figure 2.**
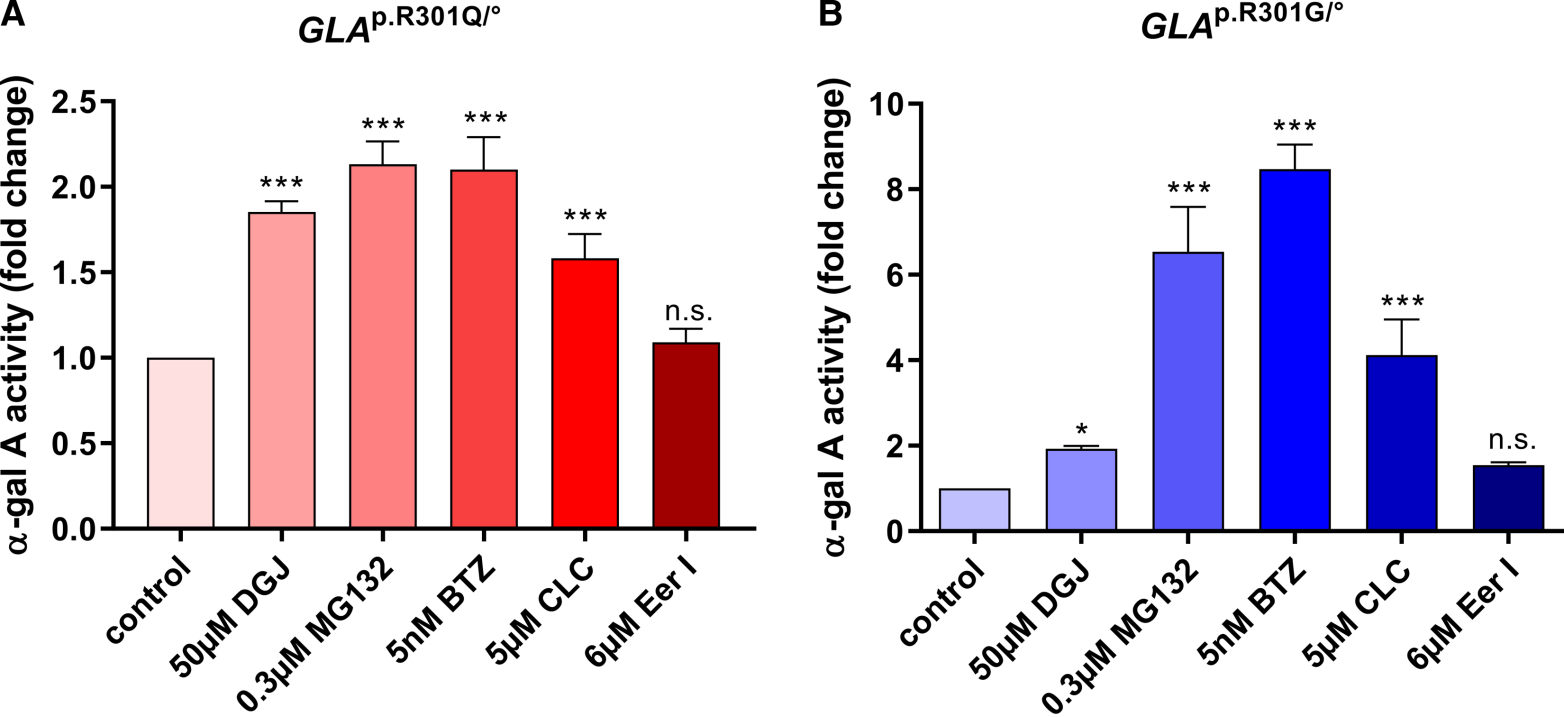
Enhancement of mutant α-Gal A activity by proteostasis regulators. *GLA*^p.R301Q/^° (**A**) and *GLA*^p.R301G/^° (**B**) fibroblasts were treated for 5 days with the pharmacological chaperone DGJ and different proteostasis regulating drugs (MG132, BTZ, CLC and EerI). Reported enzyme activity was normalized to the activity of untreated mutant control cells. Data are reported as mean ± SD of at least three independent experiments. One-way ANOVA with post-hoc Dunnett test was carried out to test statistical significance (*, ***P-values of 0.05 and 0.001); n.s., not significant.

In this study, we first screened 23 PRs as potential variant α-Gal A activity enhancers (Table 1). To this end, *GLA*^p.R301Q/^° fibroblasts were treated with proteostasis regulating substances of varying concentrations for 5 days. For the treatment with DGJ and all DGJ combinations, a 6-h non-treatment phase was applied before cells were harvested (see Materials and methods). A substance was considered effective if the mean value of enzyme activity increased by more than 1.2 times compared with untreated *GLA*^p.R301Q/^° fibroblasts and statistical significance was obtained from at least three independent measurements. The proteasome inhibitors MG132, BTZ and CLC were classified as effective activity enhancers of variant p.R301Q. Enzyme activity in *GLA*^p.R301Q/^° cells was elevated up to 2.1-fold by MG132 and BTZ, which is comparable to the 1.9-fold increase observed with 50 µM DGJ (Figure 2A). CLC increased the activity of α-Gal A up to 1.7-fold. The concentration-dependent effect of the active PRs is shown in Supplementary Figure S3. When testing the substances with *GLA*^p.R301G/^° fibroblasts, the concentrations most effective in the *GLA*^p.R301Q/^° cells showed a high efficacy, enhancing the variant p.R301G enzyme up to 6.5-fold (MG132), 8.5-fold (BTZ) and 4.1-fold (CLC), respectively (Figure 2B).

**Table 1:** Panel of proteostasis regulators used in this study

Molecular function	Small molecule	Disease examined	Proteostasis regulator reference
Ca^2+^ channel blocker	Lacidipine	Gaucher disease	Wang et al. *Chem Biol.* (2011)
	Dantrolene	Gaucher disease	Wang et al. *ACS Chem. Biol.* (2011) Ong et al. *Nat. Chem. Biol.* (2010)
	Diltiazem	Gaucher disease	Ong et al. *Nat. Chem*, *Biol*. (2010)
Coinducer of heat shock proteins (HSPs)	Arimoclomol	NPC1	Kirkegaard et al. *Sci. Transl. Med.* (2016)
Inhibitor of cyclooxygenase	Ibuprofen	Cystic fibrosis	Carlile et al. *J. Cyst Fibros.* (2015)
ERAD inhibitor	17-AAG (HSP90)	Glioblastoma multiforme	Sauvageot et al. *Neuro Oncol.* (2009)
	Bortezomib (proteasome)	Pompe disease	Shimada et al. *JIMD Rep.* (2015)
	Celastrol (proteasome)	Gaucher disease, Tay-Sachs disease	Mu et al. *Cell* (2008)
	Clasto-Lactacystin β-lactone (proteasome)	Fabry disease	Ishii et al. *Biochem. J.* (2007)
	Eeyarestatin I (VCP)	Gaucher disease	Wang et al. *J. Biol. Chem.* (2011)
	Kifunensine (MAN1B1)	Gaucher disease	Wang et al. *J. Biol. Chem.* (2011)
	MG132 (proteasome)	Gaucher disease, Tay-Sachs disease	Mu et al. *Cell* (2008)
	Pifithrin-µ (HSP70)	Cancer	Leu et al. *Mol. Cell* (2009)
	Pyr41 (ubiquitination)	Fabry disease	Lukas et al. *Mol. Ther.* (2015)
	Ritonavir (proteasome)	Solid malignancies	Kraus et al. *Mol. Cancer Ther.* (2008)
	SAHA (histone deacetylase)	NPC1, Gaucher disease	Pipalia et al. *Proc. Natl Acad. Sci. U.S.A.* (2011), Lu et al. *Proc. Natl Acad. Sci. U.S.A.* (2011)
	TSA (histone deacetylase)	NPC1	Pipalia et al. *Proc. Natl Acad. Sci. U.S.A.* (2011)
Na^+^ channel blocker/PC	Ambroxol	Fabry disease/Pompe disease, Gaucher disease	Lukas et al. *Mol. Ther.* (2015), McNeill et al. *Brain* (2014)
PC	DGJ	Fabry disease	Fan et al. *Nat. Med.* (1999)
Peroxisome Proliferator-Act-ivated Receptor agonist	Rosiglitazone (PPARγ)	Fabry disease	Lukas et al. *Mol. Ther.* (2015)
	Pioglitazone (PPARγ)	Alzheimer's disease	Papadopoulos et al. *PLoS One* (2013)
	15d-PGJ_2_ (PPARγ)	Multiple myeloma	Sperandio et al. *Exp. Mol. Pathol*. (2017)
	Bezafibrate (PPARα/δ/γ)	Fabry disease	Lukas et al. *Mol Ther.* (2015)

### Proteostasis regulators with synergistic effects in combination with DGJ

**Figure 3.**
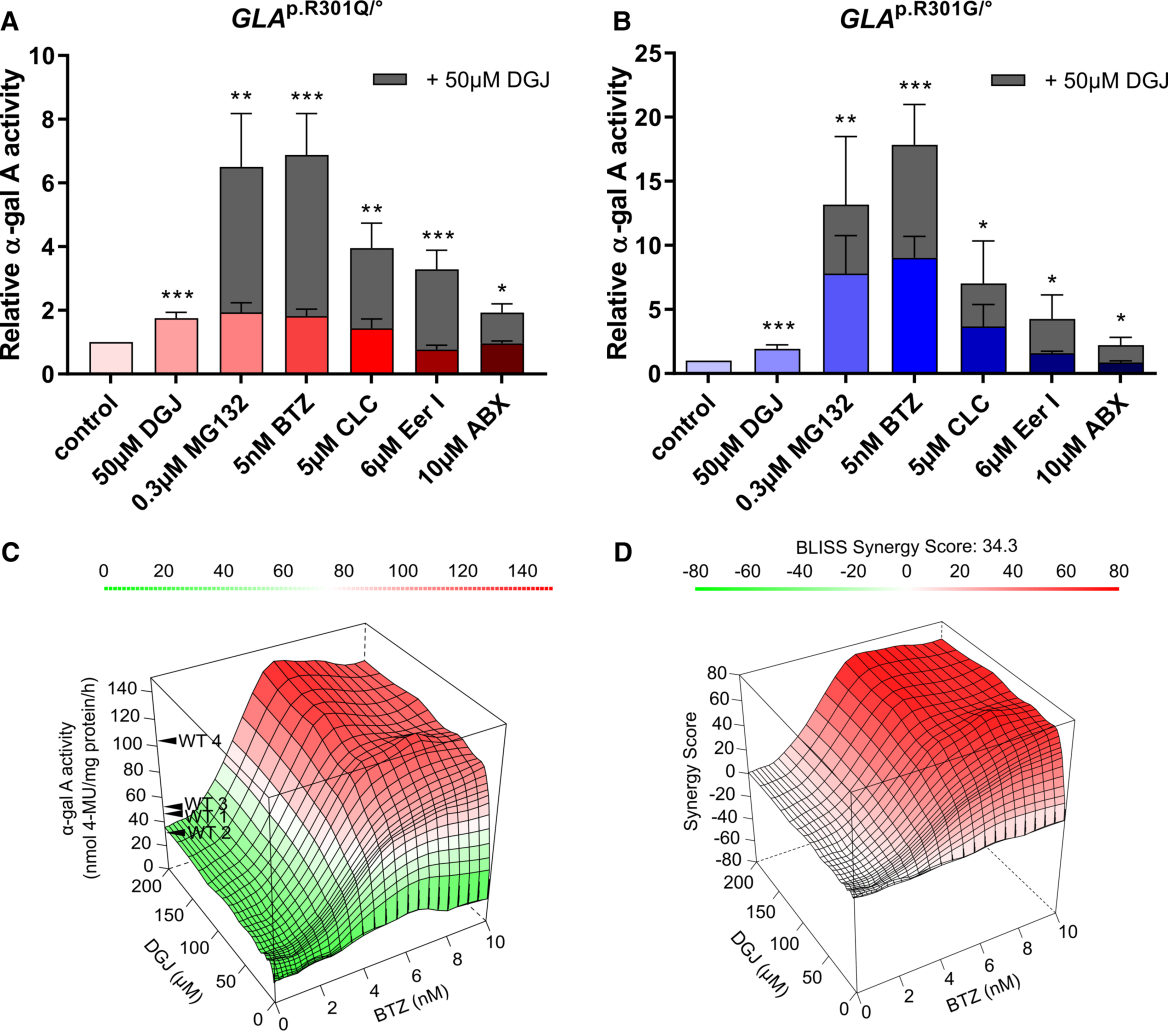
Drug synergy of PRs and the pharmacological chaperone DGJ on Fabry disease mutants. *GLA*^p.R301Q/^° (**A**) and *GLA*^p.R301G/^° (**B**) fibroblasts, respectively, were treated with combinations of DGJ and various PRs. Reported enzyme activity was normalized to the activity of untreated mutant control cells. For the statistical evaluation, DGJ treatment was compared with the untreated condition, the combinations were compared with DGJ single treatment. (**C**) Drug interaction landscape for enzyme activity in *GLA*^p.R301Q/^° fibroblasts was established for DGJ and BTZ. **(D)** Drug interaction landscape for enzyme activity in *GLA*^p.R301Q/^° fibroblasts based on the BLISS model revealed BLISS Synergy Score of 34.3 as the representative mean of all calculated single Synergy Scores. Data in (**A**) and (**B**) are representative of mean ± SD of at least three independent experiments. Data in (**C**) and (**D**) are reported as mean of 3 independent experiments. Statistics (**A**,**B**): One-way ANOVA with post-hoc Dunnett test (*, **, ***P-values of 0.05, 0.01 and 0.001).

Both the *GLA*^p.R301Q/^° and the *GLA*^p.R301G/^° cell lines were treated with a combination of the PRs and 50 µM DGJ. After 5 days of treatment, α-Gal A activity was significantly increased when compared with the single treatment with DGJ (Figure 3A,B). Evidently, MG132 and BTZ increased the DGJ effect from 2-fold up to 6.5-fold and 6.8-fold, respectively, in *GLA*^p.R301Q/^° cells, and up to 13.2-fold and 17.8-fold, respectively, in *GLA*^p.R301G/^° cells. The combination of DGJ and CLC resulted in a 4-fold and 7-fold increase in enzyme activity in *GLA*^p.R301Q/^° and *GLA*^p.R301G/^° fibroblasts, respectively. EerI, which inhibits the Sec61-mediated protein translocation from the ER into the cytosol, had no significant effect in *GLA*^p.R301Q/^° fibroblasts when used as a single substance (Figure 2A), but in combination with DGJ, a significant effect beyond that of DGJ single treatment was observed up to 3.3-fold of the untreated and 1.9-fold of the DGJ single treated state, respectively (Figure 3A). In *GLA*^p.R301G/^° cells a 4.3-fold increase above untreated and 2.2-fold above DGJ single treatment was achieved (Figure 3B). Hence, EerI was evaluated as an effective substance. The secretolytic and mucoactive agent Ambroxol (ABX) was formerly described as a potential PC for FD [35]. ABX at a concentration of 10 µM slightly elevated the effect obtained with DGJ single treatment by 1.1-fold in *GLA*^p.R301Q/^° (Figure 3A) and by 1.3-fold in *GLA*^p.R301G/^° fibroblasts (Figure 3B), respectively. Even though this was a bit less than the effect observed on these two variants in HEK293H cells in the previous study, ABX was included in the further analyses. The remaining PRs listed in Table 1 were inactive in both single and combination treatment with DGJ (data not shown).

Based on the BLISS independence model, the synergistic mechanism of action was determined for the combinations of DGJ plus BTZ. The BLISS model provides a formula for the investigation of the interaction of two simultaneously applied compounds to determine the efficacy of combination treatments. To examine the dynamics of the most effective combination treatment, different concentration ranges of DGJ (1–200 µM) and BTZ (1–10 nM) were combined for the treatment of *GLA*^p.R301Q/^° fibroblasts (Figure 3C). Exceeding the clinically achievable plasma concentration by 20 times, DGJ at a concentration of 200 µM triggered an increase in α-Gal A activity up to 60% of the mean normal activity obtained from four WT fibroblast lines (35.3 (±5.4) nmol 4-MU/mg protein/h). However, only 5 nM BTZ were sufficient to increase the activity of variant α-Gal A in combination with the clinically effective 10 µM DGJ up to the normal level (84.6 (±6.8) nmol 4-MU/mg protein/h). The maximum effect was achieved with the combination of 200 µM DGJ + 5 nM BTZ. To get a better overview of the synergy of DGJ and BTZ, an analysis based on the BLISS independence model was performed. Drug interaction of BTZ and DGJ in the *GLA*^p.R301Q/^° fibroblasts featured synergy as demonstrated by the BLISS synergy scores of the combined treatment (Figure 3D).

### Bortezomib corrects the Fabry-related cellular phenotype

**Figure 4.**
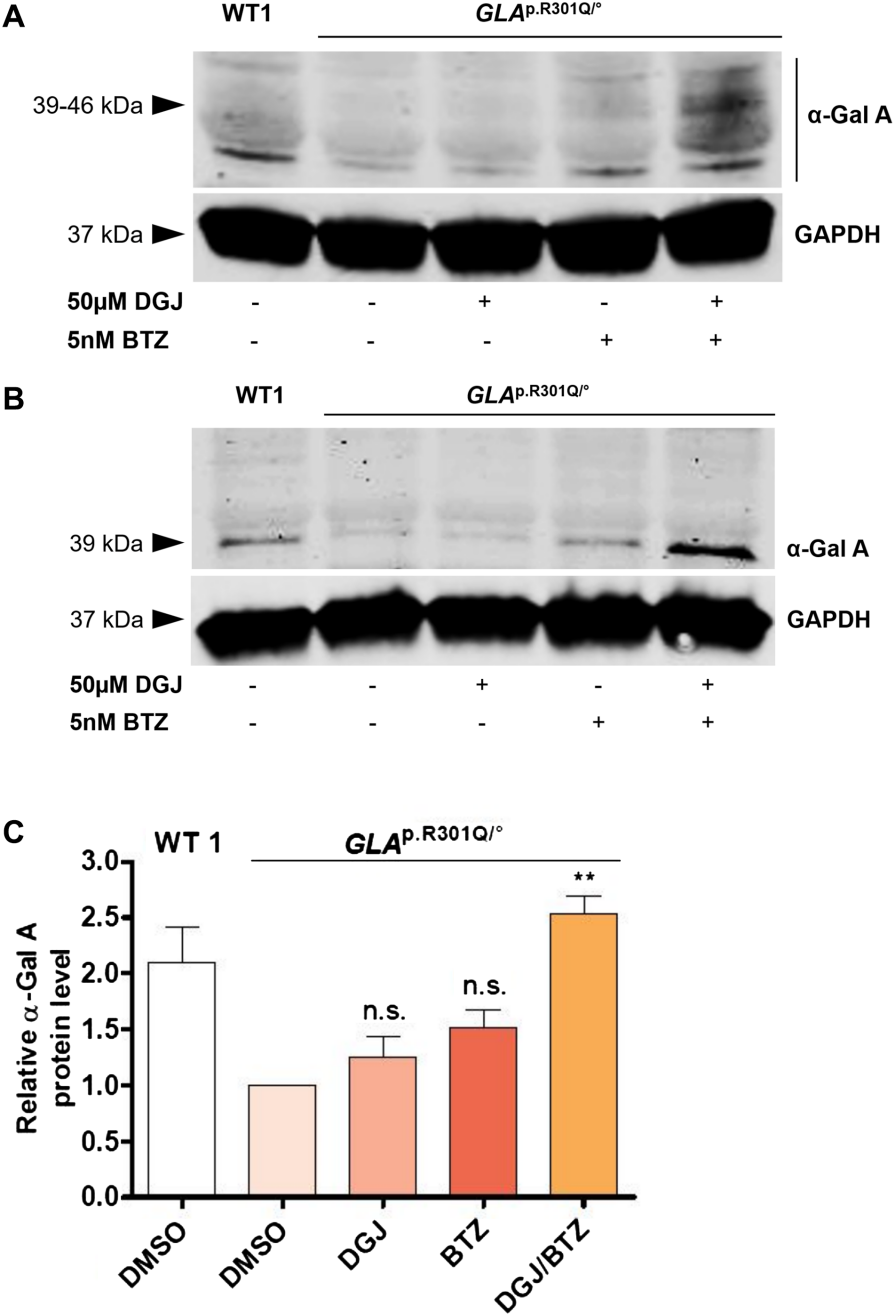
Protein level of α-Gal A in *GLA*^p.R301Q/^° fibroblasts after treatment with DGJ and BTZ. WT1 cells and *GLA*^p.R301Q/^° fibroblasts were treated for 5 days with 50 µM DGJ and 5 nM BTZ with subsequent washout for 6 h. (**A**) Protein level of α-Gal A was reduced in *GLA*^p.R301Q/^° fibroblasts in comparison with WT1 cells. Treatment with DGJ and BTZ increased the protein level of α-Gal A in *GLA*^p.R301Q/^° fibroblasts above the WT level. (**B**) Deglycosylation with PNGase F prior to western blot resulted in a condensation of all antibody-reactive material in a distinct band at ∼39 kDa. (**C**) Quantification of (**B**). Fluorescence intensity of the α-Gal A bands was normalized to the corresponding GAPDH band and to untreated *GLA*^p.R301Q/^° cells. Data represent three independent experiments and are plotted as mean ± SD. Statistics: Differences between the groups of treated *GLA*^p.R301Q/^° fibroblasts were analyzed using One-way ANOVA with post-hoc Dunnett test (**P-value of 0.01); n.s., not significant.

The α-Gal A protein undergoes a complicated process involving folding and transport in the cell en route to the lysosome. Higher enzyme activity should correlate with an improved processing of variant α-Gal A. For a quantitative assessment of α-Gal A, *GLA*^p.R301Q/^° fibroblasts were treated with DGJ and BTZ for 5 days with subsequent washout for 6 h. The occurrence of several N-glycosylated α-Gal A forms renders its quantification difficult (Figure 4A). *In vitro* deglycosylation of the enzyme prior to western blot analysis using PNGase F was applied to collect all cellular α-Gal A forms in a distinct band for a more comprehensive measurement (Figure 4B). Quantification of the indicated 39 kDa band revealed a reduced α-Gal A protein level by half in the *GLA*^p.R301Q/^° cell line in relation to WT1 fibroblasts (Figure 4C). α-Gal A level tended to be slightly elevated after the single treatment with 50 µM DGJ or 5 nM BTZ to 1.3-fold and 1.5-fold, respectively (Figure 4C). In accordance with the enzyme activity measurement (Figure 3A), the combined treatment with DGJ/BTZ increased the α-Gal A to supraphysiological levels.

**Figure 5.**
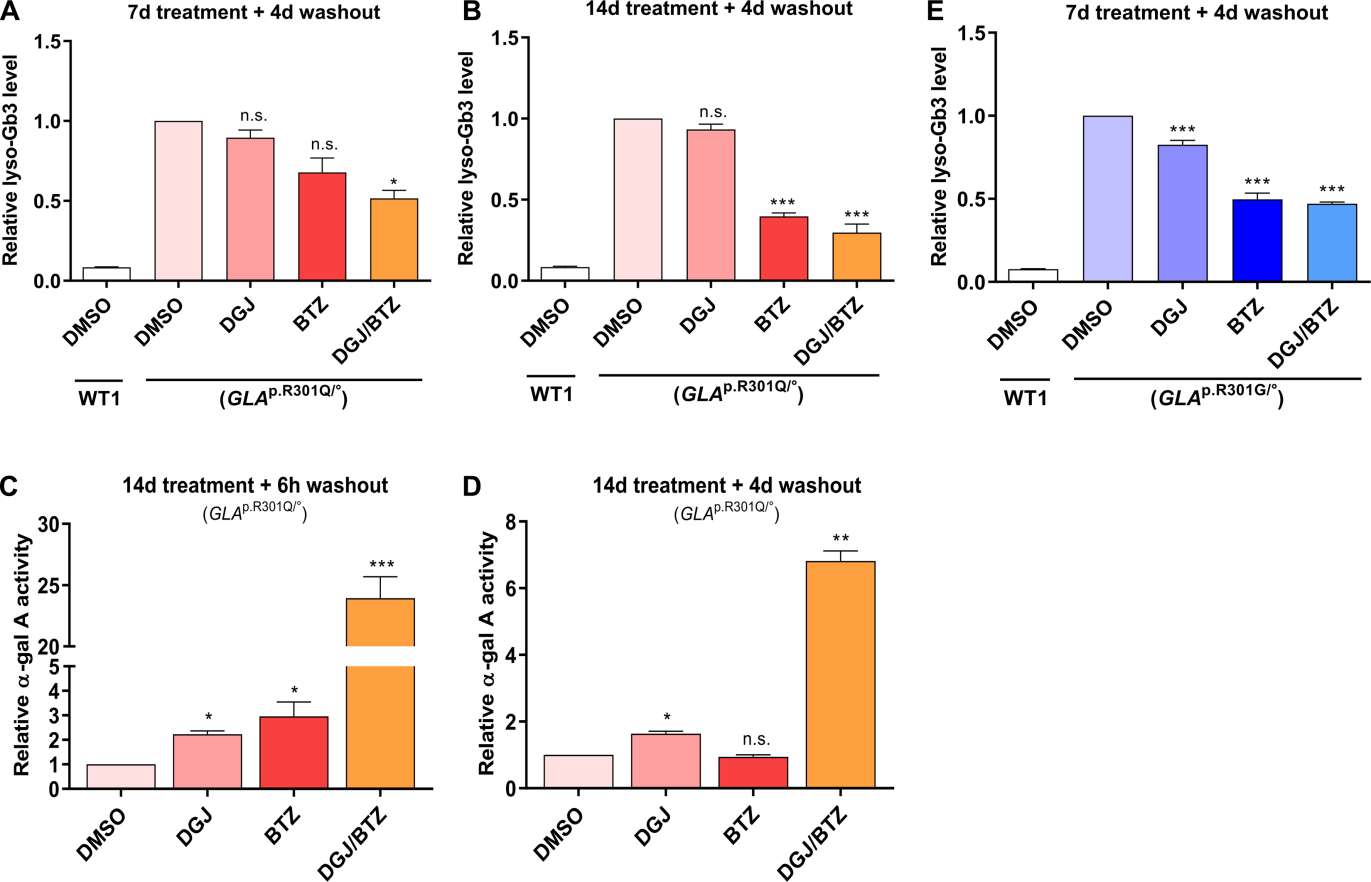
Lyso-Gb3 level and α-Gal A activity in *GLA*^p.R301Q/^° cells in response to prolonged treatment with DGJ and BTZ. *GLA*^p.R301Q/^° fibroblasts were treated for 7 days (**A**) or 14 days (**B**) with 50 µM DGJ, 5 nM BTZ and the DGJ/BTZ combination with subsequent washout for 4 days. Lyso-Gb3 values were normalized to DMSO-treated *GLA*^p.R301Q/^° cells. The white barshows the lyso-Gb3 level in DMSO-treated WT1 fibroblasts. (**C**) α-Gal A activity after the prolonged 14-day treatment and 6-h washout analogous to the described treatment scheme (Figure 2) and (**D**) an additional 4-day washout. Reported enzyme activity was normalized to the activity of untreated mutant control cells. For the statistical evaluation, DGJ and BTZ single treatments were compared with the untreated condition, the combination was compared with DGJ single treatment. (**E**) *GLA*^p.R301G/^° fibroblasts were treated for 7 days as in **(A)** and lyso-Gb3 was normalized to the DMSO-treated cells. All results are normalized to the untreated *GLA*^p.R301Q/^° cells. Data are plotted as mean ± SD from 4 (**A**) or 3 (**B–E**) independent experiments. Statistics: Differences between the groups were analyzed using One-way ANOVA with post-hoc Dunnett test (*, **, ***P-values of 0.05, 0.01 and 0.001); n.s., not significant.

It is not possible to prove with certainty how high a potential gain in protein stability or enzyme activity must be in order to restore normal cell physiology and, hence, provide a clinical benefit. Therefore, an important result of the treatment is a functional reduction in pathophysiological lyso-Gb3 levels. *GLA*^p.R301Q/^° fibroblasts were treated with 50 µM DGJ and 5 nM BTZ for 7 and 14 days with a subsequent washout for 4 days. After 7 (+4) days, DGJ (10%) and BTZ (32%) single treatment caused an insignificant reduction in lyso-Gb3 in the cells (Figure 5A). In contrast, the combination of DGJ and BTZ lowered the cellular lyso-Gb3 significantly by 49%. Increasing treatment time to 14 days did not improve the effect of DGJ but enhanced the BTZ effect after single treatment (60%) as well as in combination with DGJ (70%) (Figure 5B). However, the differences between the 7-day and 14-day treatment have not been significant being *P* = 0.2140 for the single and *P* = 0.4081 for the combination treatment (unpaired two-sample *t*-test). The lyso-Gb3 clearance after the single and combined treatment with DGJ and BTZ followed relatively slow kinetics. It behaves in an almost linear way within the monitored time of the experiment. Moreover, the lyso-Gb3 level is far from normal, being 3.8 times higher than in the control fibroblast cell line after the 14-day treatment. Enzyme activity, however, approached (in case of DGJ, BTZ single treatment) or even exceeded (in case of combined treatment) the normal level after 5 days (compare Figures 2A and 3A). Therefore, we wanted to determine how enzyme activity changes during prolonged exposure to treatment and whether the effect can become exhausted, or potentially, increase over time. At first, only the duration of treatment was adjusted to 14 days, but the washout time was left at 6 h specified for DGJ (Figure 5C). As expected, enzyme activity increase after DGJ and BTZ single treatments remained relatively stable compared with 5 days. A slight trend was observed indicating a stronger increase using longer incubation periods (DGJ: 1.9-fold (5 days) vs. 2.3-fold (14 days), *P* = 0.0173; BTZ: 2.1-fold (5 days) vs. 3.0-fold (14 days), *P* = 0.0921, unpaired two-sample *t*-test). The combined treatment was significantly more effective and achieved a 24-fold increase in the initial activity (DGJ/BTZ: 6.8-fold (5 days) vs. 24.0-fold (14 days), *P* < 0.0001, unpaired two-sample *t*-test). We then adjusted the washout period likewise to 4 days in accordance with the lyso-Gb3 experiments (Figure 5D). The DGJ effect resembled the level observed for the 5-day treatment and 6-h washout regimen while the beneficial effect of BTZ single treatment on the α-Gal A activity could not be observed after 4 days of washout. The combined treatment of DGJ and BTZ yielded an elevation identical with the level observed for 5-day treatment and 6-h washout (6.8-fold). Although the combination treatment after 4 days of washout still resulted in a significant increase in activity compared with the DGJ single treatment, the reduced activity compared with the 14-day treatment and 6-h washout (6.8-fold vs. 24.0-fold, *P* < 0.0001, unpaired two-sample *t*-test) indicates that a part of the BTZ effect was eliminated by the discontinuation of treatment.

Altogether, these data suggest that the DGJ effect on α-Gal A will persist for prolonged periods of time when treatment is discontinued. Although the effect produced by BTZ is temporary and decreased rapidly when cells were no longer exposed to treatment, the BTZ effect could reach very high levels through prolonged treatment time (Figure 5C). Moreover, the lyso-Gb3 reducing effect of BTZ also appears to be sustainable in the interrupted treatment regime (Figure 5A,B). The *GLA*^p.R301G/^° fibroblasts showed a significant reduction in lyso-Gb3 with all applied substances using the 7 (+4)-day washout treatment regimen (Figure 5E) and confirmed the principle trend in the *GLA*^p.R301Q/^° cells.

### Proteostasis regulators display different effects on the proteasome

**Figure 6.**
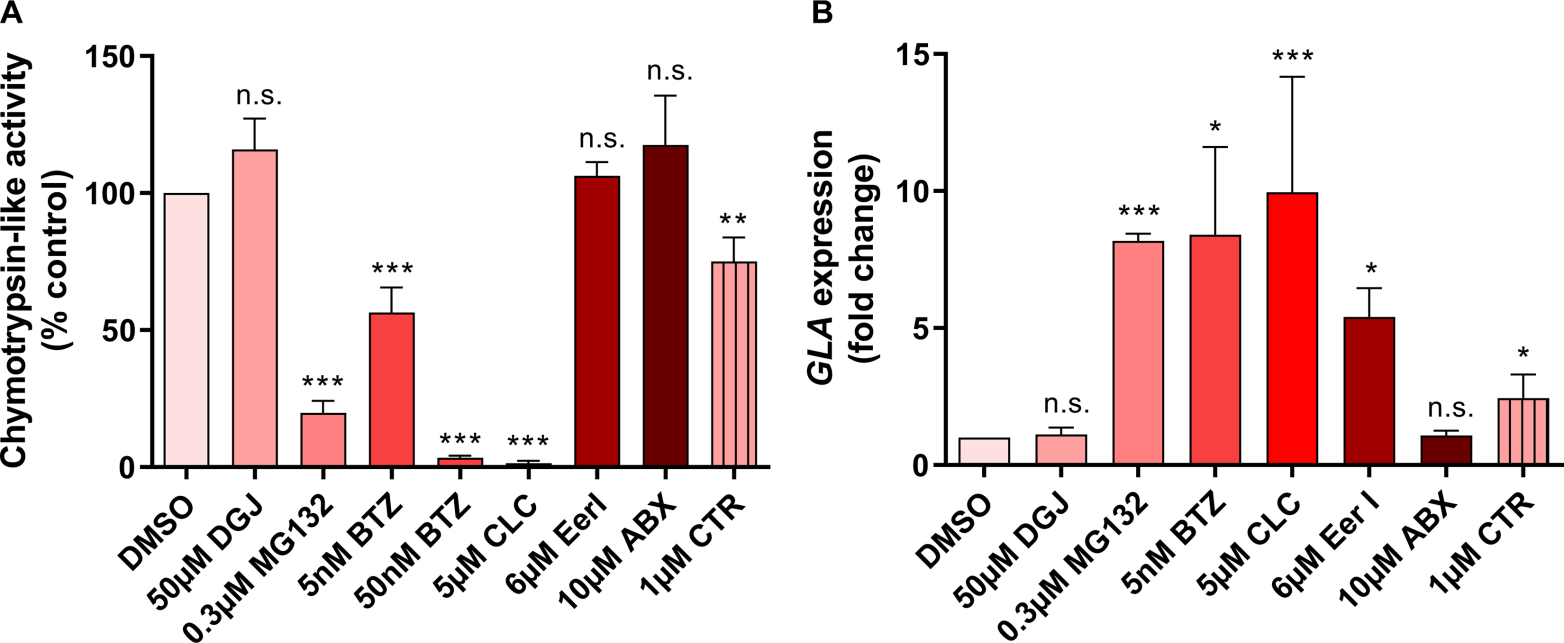
PRs show distinct effects on proteasomal activity and *GLA* gene expression in *GLA*^p.R301Q/^° fibroblasts. (**A**) Proteasomal activity under the influence of proteostasis regulators. *GLA*^p.R301Q/^° fibroblasts were PR-treated for 2 h and immediately harvested in PBS. The obtained cell suspensions were then used for the luminometric measurement of chymotrypsin-like proteasomal activity. Results were normalized to the DMSO-treated *GLA*^p.R301Q/^° cells. The data are reported as mean ± SD of five independent experiments. Statistics: One-way ANOVA with post-hoc Dunnett test (*, **, ***P-values of 0.05, 0.01 and 0.001); n.s., not significant. (**B**) PRs with different effects on *GLA* expression. Relative *GLA* mRNA expression was analyzed by quantitative RT-PCR. Ahead of RT-PCR, *GLA*^p.R301Q/^° fibroblasts were treated for 24 h with indicated concentrations of PRs. The data represent 3–5 independent experiments and are plotted as mean ± SD relative to DMSO treatment. Statistics: Differences between the groups were analyzed using One-way ANOVA with post-hoc Dunnett test (*, **, ***P-values of 0.05, 0.01 and 0.001); n.s., not significant.

The proteasome is a major effector of the PRs by definition, and MG132, BTZ and CLC are known inhibitors of the proteasomal activity. The decrease in proteasomal function in *GLA*^p.R301Q/^° fibroblasts was observed after the application of the effective concentrations of MG132, BTZ and CLC, but the level of reduction was obviously very different being 19.8%, 56.5% and 1.5% of the normal activity, respectively (Figure 6A). A concentration of 50 nM BTZ could further lower the proteasomal activity but had no additional effect on variant α-Gal A activity (Supplementary Figure S3C). Thus, BTZ was most effective in increasing the activity of α-Gal A at sub-IC50 concentrations related to proteasomal inhibition [48]. Application of DGJ, EerI and ABX had no effect on the proteasome. Celastrol (CTR) was included in this analysis as a proteasomal inhibitor that was unable to increase α-Gal A activity in the *GLA*^p.R301Q/^° cells in order to obtain a mechanistic explanation for this finding. Treatment with CTR resulted in an inhibition of the proteasomal activity down to 75.0%. It is crucial to investigate whether there is a critical threshold of proteasomal activity necessary for the rescue of variant α-Gal A or, more generally, whether regulation of the degradation of α-Gal A via the proteasome is a relevant factor at all.

### *GLA* gene expression elevation in *GLA*^p.R301Q/^° cells by proteostasis regulators

PRs have an impact on the transcriptome. An obvious suspicion is that the PRs investigated here up-regulate the mutated *GLA* gene itself. Thus, we tested whether the effective PRs could trigger *GLA* gene expression in the *GLA*^p.R301Q/^° fibroblasts. MG132, BTZ, CLC and EerI induced increases in expression levels of up to 7.9-fold, 8.4-fold, 9.2-fold and 5.4-fold (Figure 6B). The application of DGJ and ABX had no effect on *GLA* expression. CTR increased the expression level of *GLA* up to 2.6-fold which indicates that the ability to elevate *GLA* gene expression is an important, but not an exclusive attribute of a PR that acts as an enhancer of α-Gal A activity, and it appears that a critical limit must be exceeded to trigger a functional enzyme activity boost.

### Potential role of transcriptional regulation in α-Gal A activity restoration

Given the global impact of PRs on the transcriptome and the proteostasis interactome, in particular, it is of great relevance to identify genes whose expression level in the cell is significantly altered by the effective PRs. Unbiased transcriptional profiling could unveil deeper mechanistic insights into PR function and lead to the identification of new therapeutic targets. Whole transcriptome microarray analysis was therefore performed on *GLA*^p.R301Q/^° fibroblasts after treatment with DGJ and the effective PRs.

We performed principal component analysis (PCA) on the global gene expression profiles (29 799 probesets) (Supplementary Figure S4). Broadly, the samples can be grouped into three clusters, which are distinguished by their PC1 scores, having high inter- and low intra-cluster variance. One of the clusters comprises the untreated DMSO controls, single treatments of ABX and DGJ and the combination treatment thereof. This indicates that the global impact of those treatments on the transcriptome is rather limited. On the other hand, a cluster containing single treatments of BTZ and MG132 and the combination treatment of the latter with DGJ is located at a considerable distance from the cluster containing the controls, indicating a major impact on transcription. This is in line with our findings on the phenotypic effects of these proteostasis modifiers as shown above. Finally, a cluster containing CLC and EerI features PC1 scores close to zero; this suggests that the transcriptomic effect of those drugs is smaller than that of MG132 and BTZ. Furthermore, the underlying gene expression changes are likely to affect distinct sets of genes, implying different modes of action of the drugs.

The treatment with MG132 and BTZ regulated the expression of 1332 and 1060 genes, respectively (Supplementary Tables S2 and S3). CLC and EerI changed the expression of 471 and 512 genes, respectively. The intersection of those four sets of differentially expressed genes contained 235 genes. Treatment with DGJ and ABX caused no substantial change in gene expression, with zero and two differentially expressed genes, respectively. We identified 86 biological pathways by enrichment analysis using the WikiPathways collection [49] that show gene content overlap with the 235 consensus genes, and calculated the statistical significance thereof; Table 2 gives the most significant pathways (*P* ≤ 0.001, hypergeometric test) of which Hs_Proteasome_Degradation (WP183), Hs_Parkin-Ubiquitin_Proteasomal_System_pathway (WP2359) and Hs_NRF2_pathway (WP2884) stand out, as these are directly proteostasis-related pathways. Other pathways such as Hs_Histone_Modifications (WP2369), Hs_Cell_Cycle (WP179) and Hs_Nucleotide_Metabolism (WP404) may be indicative of involved gene regulatory mechanisms. An effect on various proteostasis-associated pathways can also be demonstrated using the differentially expressed genes of each individual treatment (Supplementary Tables S4–S7).

**Table 2:** Overrepresented signaling pathways within the intersection of global transcriptional signatures obtained from MG132, BTZ, CLC and EerI

No.	WIKI pathway
**1**	**Hs_Proteasome_Degradation_WP183**
2	Hs_Histone_Modifications_WP2369
3	Hs_Retinoblastoma_(RB)_in_Cancer_WP2446
**4**	**Hs_Parkin-Ubiquitin_Proteasomal_System_pathway_WP2359**
5	Hs_Gastric_Cancer_Network_1_WP2361
6	Hs_Pentose_Phosphate_Pathway_WP134
**7**	**Hs_NRF2_pathway_WP2884**
8	Hs_Benzo(a)pyrene_metabolism_WP696
9	Hs_Cell_Cycle_WP179
10	Hs_Polyol_Pathway_WP690
11	Hs_Nucleotide_Metabolism_WP404
12	Hs_Oxytocin_signaling_WP2889

Shown are the significantly overrepresented pathways with the 12 lowest corresponding *P*-values. bold: proteostasis-associated pathways.

**Figure 7.**
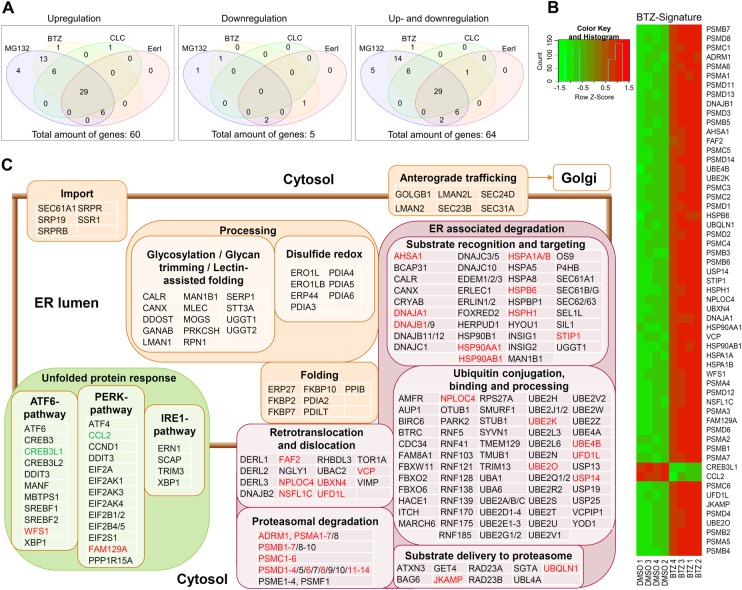
Proteostasis-associated transcriptional signature of PR-treated *GLA*^p.R301Q/^° fibroblasts. (**A**) VENN diagram of differentially expressed proteostasis genes after the treatment of *GLA*^p.R301Q/^° fibroblasts with PRs. An intersection of 29 proteostasis genes was differentially expressed in all four treatments. The majority of the proteostasis genes was up-regulated and only a few were down-regulated. For detail, please refer to Supplementary Tables S8 and S9 (**B**) Heatmap of the 57 BTZ-regulated genes. (**C**) Visualization of the proteostasis gene signature of BTZ on a functional chart of ERAD/proteostasis. The chart includes 254 of the 357 manually reviewed proteostasis components based on their biological function in the network. Differentially expressed genes are indicated in red (up-regulation) and green (down-regulation). Differential gene expression was defined by >1.5-fold difference with a *P*-value threshold of 0.05.

To obtain a more proteostasis/ERAD focused view, we intersected the list of differentially expressed genes with our proteostasis genes identified (see Materials and methods). Indeed, of the 357 proteostasis-related genes, 6 were not annotated on the microarray and, hence, excluded from the analysis, and 64 (18.2%), were differentially expressed in at least one of the treatments (Figure 7A, Tables 3, Supplementary Tables S8 and S9), significantly more than expected by chance (*P* ≤ 4.13 × 10^−20^, hypergeometric test). It is of note that of those 64 differentially expressed genes, 60 were up-regulated, while only 4 were down-regulated, in at least one treatment (Figure 7A). Altogether, 29 signature genes were common to all four treatments (BTZ, MG132, CLC and EerI). Fifty-seven genes underwent differential expression upon BTZ treatment (Figure 7B). Mapping these genes on the functional chart of proteostasis suggests their involvement in subcategories like ‘misfolded protein recognition’ and ‘proteasomal degradation’ (Figure 7C). To investigate whether there were already differences in baseline gene expression in the *GLA*^p.R301Q/^° cells, we compared the gene expression profiles of the FD cells with the 4 WT cell lines. PCA was applied to all 29 799 (Supplementary Figure S5A) and the 351 proteostasis related (Supplementary Figure S5B) probesets. The high inter-cluster variance was observed for all five cell lines indicating that the FD cells were no less different than the WT lines among themselves. A more detailed view at the proteostasis genes showed no signs of differential regulation or ER stress. Only 19 genes were different between the *GLA*^p.R301Q/^° cells and at least one WT line, but not a single proteostasis gene showed rectified regulation to all 4 WT lines (Supplementary Figure S5C).

**Table 3:** Number of differentially expressed proteostasis genes after the treatment with DGJ and PRs

Genes	Treatment								
	DGJ	MG132	BTZ	CLC	EerI	ABX	DGJ + MG132	DGJ + EerI	DGJ + ABX
Up-regulated genes	0	58	55	36	35	0	64	41	0
Down-regulated genes	0	4	2	0	3	0	3	2	0
Sum of regulated genes	0	62	57	36	38	0	67	43	0

## Discussion

The aim of the present study was to identify candidate small molecules able to increase mutant α-Gal A activity in patient-derived fibroblasts, and to provide a deeper understanding of the mechanisms initiated by the PRs that may be responsible for the effect on α-Gal A. We have demonstrated the efficacy of the PRs MG132, BTZ, CLC and EerI as potential drugs for FD by increasing enzyme activity of variant forms of the α-Gal A. We have further shown synergistic increase in mutant α-Gal A activity by combination of PRs with the clinically approved drug DGJ (trade name: Galafold [50]). The results of the current study can, therefore, be a valuable indication for a future clinical combination application of PRs with one of the approved treatments, e.g. the PC.

Due to a large number of variants leading to marginally stable α-Gal A protein, FD can be referred to as a protein misfolding disease for this portion of the variants. Misfolding of α-Gal A results in premature proteasomal degradation of the often still catalytically active enzyme [34]. Thus, an insufficient number of enzyme molecules are transported to the lysosomes, resulting in a diminished degradation of substrates and their accumulation within the cells and the extracellular space [6,51,52]. A first step towards the elimination of cellular dysfunction in protein misfolding diseases associated with loss-of-function mutations seems to be to increase the reduced protein amount of the damaged protein or enzyme in the cells. Proteostasis regulating drugs are interesting options to correct these particular phenotypes. In an approach to identify novel highly effective PRs and unravel their mode of action, we identified BTZ amongst others as being (i) highly effective and (ii) superior to the FDA-approved PC DGJ in attenuating hallmark pathology (increase in α-Gal A activity and decrease in biomarker lyso-Gb3 levels in patient fibroblast) by acting on several involved pathways (increasing gene expression, decreasing proteasomal activity). Of note, BTZ is an FDA-approved proteasome inhibitor for the treatment of plasmocytome (trade name: Velcade ® [53]) and is thus available for re-positioning approaches.

In the *GLA*^p.R301Q/^° fibroblasts DGJ was able to increase enzyme activity in a concentration-dependent manner. A 2-fold increase in enzyme activity restored ∼60% of wild-type activity in the *GLA*^p.R301Q/^° fibroblasts cells at a concentration of 200 µM (Supplementary Figure S3A). Nevertheless, a washout phase of 6 h after treatment had to elapse to unfold the full DGJ effect. The screening of different PRs revealed that MG132, BTZ and CLC showed the ability to increase the activity of α-Gal A as well, with concentrations of 0.3 µM (for a 2.1-fold increase), 0.005 µM (2.1-fold increase) and 5 µM (1.7-fold increase) for MG132, BTZ and CLC, respectively (Figure 2A). EerI was able to increase α-Gal A activity in combination with DGJ above the level of the single DGJ treatment and was, therefore, also regarded as an effective PR. The optimal concentration of BTZ to increase α-Gal A activity was 5 nM and, therefore, lower than BTZ concentrations used in other studies on NPC1 and Pompe disease [20,54].

The results for a second cell line, *GLA*^p.R301G/^° fibroblasts, could reproduce the efficacy of the individual PRs, using the most effective concentration established in the first cell line. It is noteworthy that the treatment with the PRs MG132, BTZ and CLC as a mono-therapy as well as in combination with DGJ showed a trend towards a stronger responsiveness of the p.R301G variant than observed in the *GLA*^p.R301Q/^° cells. EerI also tended to be effective as a mono-therapy in the *GLA*^p.R301G/^° fibroblasts (FC: 1.54 ± 0.14, not significant, Figure 2B) at a concentration of 6 µM, while no activity change was observed in *GLA*^p.R301Q/^° fibroblasts (FC: 1.09 ± 0.25, not significant, Figure 2A). DGJ, on the other hand, demonstrated a comparable efficacy in both fibroblast lines. It is still too early to conclude about the different responsiveness of the two *GLA* variants since only one cell line per genotype was tested. Although there is not much work on genetic and epigenetic factors as FD phenotype modifiers, it can be assumed that there are factors independent of the primary *GLA* gene mutation that have a direct influence on α-Gal A activity in cell culture and may thus also have an influence on the PR effect. Yet, it is not unlikely that the p.R301G variant may have a higher effect potential for the PRs used. Since p.R301Q and p.R301G are known DGJ amenable *GLA* gene variants [5,55], we can only speculate whether non-amenable variants also respond positively to the PRs. Due to the observed promising effects on variant enzyme activity and the biomarker lyso-Gb3, an extension of the mutation spectrum should be considered in future studies. Even though we only assessed cells from male hemizygous FD patients, PR treatment is a mutation-specific therapy such as the PC therapy. Therefore, the tested drugs have the potential for usage in both male and female patients likewise as is the case with PC therapy, provided the therapy is approved for the respective variant [56].

The approach to administer combinations of a chaperone and a compound with the capability to remodel cellular proteostasis is relatively new, but it has already been shown to be a promising approach to render future therapies more efficient [57]. In the present study, it was demonstrated for the first time that the use of DGJ in combination with the PR BTZ leads to a synergistic increase in variant α-Gal A activity. The combination of the clinically approved DGJ and BTZ even normalized α-Gal A activity in the patient cells. The combination of therapeutically used 10 µM DGJ [58] with 5 nM BTZ increased the enzyme activity in *GLA*^p.R301Q/^° fibroblasts up to the normal level (compare Figure 3C). It is worth mentioning that the maximum plasma concentration of BTZ during standard therapy of patients with multiple myeloma is ∼290 nM [53,59]. The most effective concentration of BTZ for increasing α-Gal A activity in this study was 5 nM. This high potency of BTZ with regard to enzyme activity increase is a good prerequisite for initiating long-term studies with BTZ. Since FD is a progressive genetic disorder and the therapy has to last a lifetime, the use of a low concentration formulation will likely reduce adverse effects.

It has already been described that lyso-Gb3 contributes to the pathophysiology of FD [3,60]. A recent study suggests plasma lyso-Gb3 as an appropriate clinical marker to measure the biochemical response to DGJ since the levels were found to reflect the disease course in the patients examined [61]. Lyso-Gb3 induces the proliferation of smooth muscle cells *in vitro* [3], inhibits cell growth and differentiation of fibroblasts [62] and contributes to the sensitization of peripheral nociceptive neurons [60]. Thus, therapeutic intervention is expected to reduce lyso-Gb3 in order to improve FD pathology. After a period of 7 days the BTZ treatment lowered lyso-Gb3 by 32% in *GLA*^p.R301Q/^° fibroblasts (Figure 5A), after 14 days, the cells demonstrated a 60% reduction (Figure 5B). Even though enzyme activity was close to normal (or even completely normalized in the DGJ/BTZ combination), lyso-Gb3 clearance progressed slowly. After 14 days the level was still markedly above the level within the wild-type cells. Of note, lyso-Gb3 was only marginally reduced by DGJ even though mono-therapy was as effective as the BTZ mono-therapy when considering enzyme activity. Since DGJ is a reversible competitive inhibitor of α-Gal A and acts as active-site-specific chaperone [63], its efficacy seems to depend very much on the washout period and not on the on-treatment period, as after 7 (+4)-day and 14 (+4)-day washout phases no significant difference was found. Another study has demonstrated that Gb3 degradation was inhibited by the structurally similar lyso-Gb3 *in vitro* and *in vivo*, likely due to direct inhibition of α-Gal A [3], which may jeopardize the usefulness of ERT or another inhibitor of the enzyme such as DGJ for certain advanced FD patients with very high lyso-Gb3 levels.

In contrast, the obtained reduction using BTZ seems to be linear to the ‘on-treatment’ period, as the doubled treatment duration led to a further ∼50% reduction in the lyso-Gb3 level. BTZ function could be influenced by its ability to increase autophagy [64,65] and lysosomal exocytosis [66], which could contribute to the better clearance effect in an α-Gal A independent manner. This argues for an extension of the range of applications to patients who carry variants that do not respond sufficiently to DGJ or who do not express any functional enzyme at all, for example in combination with ERT [67]. Despite therapeutic success in FD patients harboring the amenable p.N215S α*-*Gal A variant, in which it was also noted that lyso-Gb3 was reduced under treatment, chaperone treatment may not be sufficiently effective for all gene variants whose *in vitro* amenability is proven [61]. Combination therapy using DGJ/BTZ is highly likely to be a more effective option here.

Mechanistically, we were able to highlight that an important aspect of effective PRs in FD is the ability to induce *GLA* gene expression. It has already been shown in a previous study that *GLA* expression is particularly highly responsive to MG132 treatment compared with other lysosomal genes in Gaucher patient fibroblasts [28]. This conclusion can be extended to the effect that all tested proteasomal inhibitors as well as EerI cause a significant increase in *GLA* gene expression. However, the ineffective CTR also increased *GLA* gene expression significantly. In addition, we addressed the aspect of inhibition of proteasomal activity. It has already been shown that proteasome inhibitors reduce the degradation of misfolded lysosomal proteins, thus increasing their transport to the lysosomes [15,16,53]. Our data show that the most effective concentrations of MG132, BTZ and CLC were able to inhibit the proteasome. There are indications that proteasome inhibition plays a central role in the observed effects on the α-Gal A, but since there appears to be no correlation between the intensity of proteasome inhibition and the increase in α-Gal A activity, the need for a concomitant proteasome inhibition on the quality of the α-Gal A enhancing effect needs further investigation.

Several studies describe that the mechanism of action of PRs is, amongst others, based on the regulation of gene expression of proteostasis genes [15,16,27]. Furthermore, it is known that the proteasomal system crucially influences gene expression [68]. We took a detailed look at gene expression in *GLA*^p.R301Q/^° fibroblasts and demonstrated that the effective PRs have a strong impact on global and proteostasis-associated gene expression. The transcriptional signatures of MG132, BTZ, CLC and EerI exhibited regulation of numerous ERAD genes, most of which were up-regulated. MG132 and BTZ showed similar, only slightly different, patterns regarding the globally regulated genes. Of the 1332 (MG132) and 1060 (BTZ) regulated genes, 990 were found in both signatures. Both treatments were statistically indistinguishable (*P* < 2 × 10^−16^, hypergeometric test), indicating the mechanistic identity and equiefficacy at the applied concentrations that were most effective on variant α-Gal A (0.3 µM MG132; 5 nM BTZ). However, proteasomal inhibition was significantly different at the concentrations we used (Figure 6A), which suggests a subordinate role for the strength of the inhibitory effect. This is also supported by the fact that the application of 50 nM BTZ increased the inhibition of the proteasome but did not lead to an increased α-Gal A activity (Supplementary Figure S3C). Furthermore, many proteasomal genes were induced. One reason for this might be based on the transcription factors Nrf1 and Nrf2, whose degradation is reduced under treatment with proteasomal inhibitors [69]. Downstream genes of Nrf2 are related to oxidative stress [70] and this pathway was identified by our analysis of the signature genes (Table 2). However, even though EerI slightly elevated proteasomal activity, proteasomal genes were likewise elevated. EerI inhibits the retrograde transport of proteins from the ER into the cytosol, which are intended for degradation. We speculate that a feedback pathway between proteasome and nucleus leads to an increase in the expression of proteasomal subunits, as the proteasomes cannot perceive or degrade substrate material due to this blockade.

Transcriptional signatures also demonstrated the regulation of *PERK* and *ATF6* signaling pathways while the *IRE1* pathway was not affected (Figure 7C). The latter is mainly associated with ERAD while *PERK* and *ATF6* are associated with increased folding capacities in the ER [71]. Various ER-associated chaperones were up-regulated as well, indicating an increased cellular protein folding capacity. Therefore, the effect on transcriptional regulation may be beneficial to enhance variant α-Gal A. We assume that among the differentially regulated proteostasis components, some could potentially be suitable for more specific pharmacological targeting. Although the potential to identify new treatment targets these pronounced gene expression signatures, such phenotypes may raise the question of therapeutic versus adverse effects. This aspect should not be ignored when using proteasomal inhibitors as drug candidates. DGJ, on the other hand, appears to be in this aspect an extraordinarily neutral drug.

**Figure 8.**
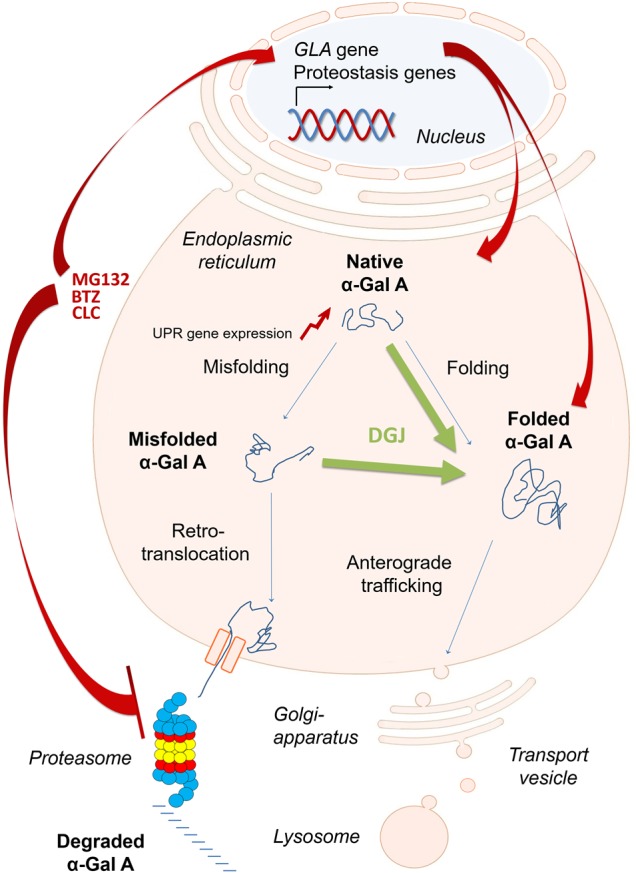
Model of the DGJ and BTZ mechanism leading to synergy in the recovery of α-Gal A function. α-Gal A level is determined by mRNA transcription and efficiency of synthesis and protein folding. In the state of falsely incorporated amino acids due to point mutations, there is a shift in the balance between folding and misfolding of the protein. The pharmacological chaperone DGJ binds to the active site of the enzyme causing the enzyme to fold into a thermodynamically more stable state and α-Gal A is then less efficiently retained by the ER quality control. BTZ inhibits proteasomal activity and may, therefore, make a higher amount of α-Gal A available for DGJ. An equally important aspect of the BTZ effect seems to be its influence on the induction of the *GLA* gene expression and, speculatively, also the influence on the expression of many other genes putatively involved in proteostasis. As a result, more α-Gal A leaves the ER and is transported to the lysosomes.

Low molecular mass compounds such as PC and PRs have proven to be promising approaches to overcoming protein folding diseases in recent years. In our study, the mechanisms of action complement each other in such a way that the synergy of the active substances can be accomplished. While DGJ increases the processing and transport of variant α-Gal A and therefore reduces its degradation [63], the studied PRs apparently mediate their effect via effective proteasomal degradation inhibition and a pronounced effect on cellular gene regulation (Figure 8). The positive-inducing effect on the *GLA* gene itself, which provides an increased amount of α-Gal A in the ER for folding and processing indicates the strong association between proteasomal and lysosomal systems. Indeed, 33 (7.6%, *P* ≤ 0.006, hypergeometric test) of the 432 annotated signature genes [72] were differentially expressed in any of the PRs treatments including genes deficient in other LSD (Supplementary Figure S6, Supplementary Table S10). In sum, the PRs had a vast effect on the cellular gene expression regulation of degradational pathways, more specifically on the proteostasis network, which is likely to support the action of the drugs.

It can be assumed that a positive effect can only be achieved if the properties of the PRs are combined in a proportion that does justice to the respective misfolded protein variant. In FD, the proteasomal inhibitors studied here seem to best fulfill this need and are therefore candidates for clinical application.

In conclusion, we identified PRs that effectively elevate mutant α-Gal A activity and reduce lyso-Gb3 in the cells. The mechanism of action of the effective PRs included marked inhibition of the proteasome and a pronounced elevation of *GLA* gene expression as two main effectors on α-Gal A enzyme activity. Additionally, we analyzed the transcriptional effects of the PRs and identified a panel of commonly regulated proteostasis genes. We suggest that these transcriptional effects describe important aspects that influence the efficacy of PRs.

## Availability of data, software and research materials

All materials used to conduct the research in this study will be made available to any researcher for purposes of reproducing the results or replicating the procedure. Affymetrix GeneChip® Human Transcriptome Arrays 2.0 CEL files are made available via Gene Expression Omnibus (GEO) database repository.

## Abbreviations

ABX: Ambroxol
BTZ: Bortezomib
CLC: Clasto-Lactacystin β-lactone
CTR: Celastrol
DGJ: 1-deoxygalactonojirimycin
EerI: Eeyarestatin I
ER: endoplasmic reticulum
ERAD: ER-associated degradation
ERT: enzyme replacement therapy
FC: fold change
FD: Fabry disease
Gb3: globotriaosylceramide
*GLA*: gene symbol of human α-Galactosidase A
HPTLC: high-performance thin-layer chromatography
LSD: lysosomal storage diseases
lyso-Gb3: globotriaosylsphingosine
n.s.: not significant
OMIM: Online Mendelian Inheritance in Man
PBS: phosphate-buffered saline
PC: pharmacological chaperone
PCA: principal component analysis
PRs: proteostasis regulators
PVDF: polyvinylidene difluoride
RT-PCR: Reverse transcription-polymerase chain reaction
UPR: unfolded protein response
WT: wild-type
α-Gal A: *GLA* gene product
