# Supplementary material for: Proteostasis regulators modulate proteasomal activity and gene expression to attenuate multiple phenotypes in Fabry disease
Source: Biochem J. 2020 Jan 30;477(2):359–80. doi: 10.1042/BCJ20190513 (PMC6993862; doi:10.1042/BCJ20190513)
Supplement: Supplementary Figures S1-S6 and Tables S1-S10 [file BCJ-477-359-s1.pdf]

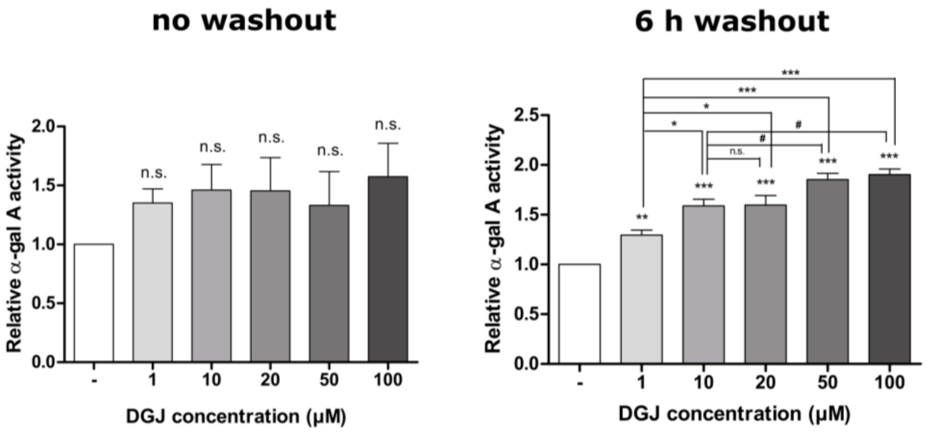

Figure S1. α-Gal A activity in *GLA*$^{p.R301Q/○}$ fibroblasts after DGJ treatment for 5 days with and without washout. When the *GLA*$^{p.R301Q/°}$ fibroblasts were treated with DGJ for 5 days, lysed directly in water after three washes with PBS and subjected to the α-Gal A activity measurement (no washout), no significant increase in enzyme activity was observed (left). If after the DGJ treatment a 6-hour phase was followed in which no DGJ was added to the cell culture medium (6 h washout), a concentration-dependent increase in α-Gal A activity was detected in the subsequent measurement (right).

Statistics: Differences between the groups were analyzed using One-way ANOVA with post-hoc Tukey test (*, **, ***P values of 0.05, 0.01, and 0.001).

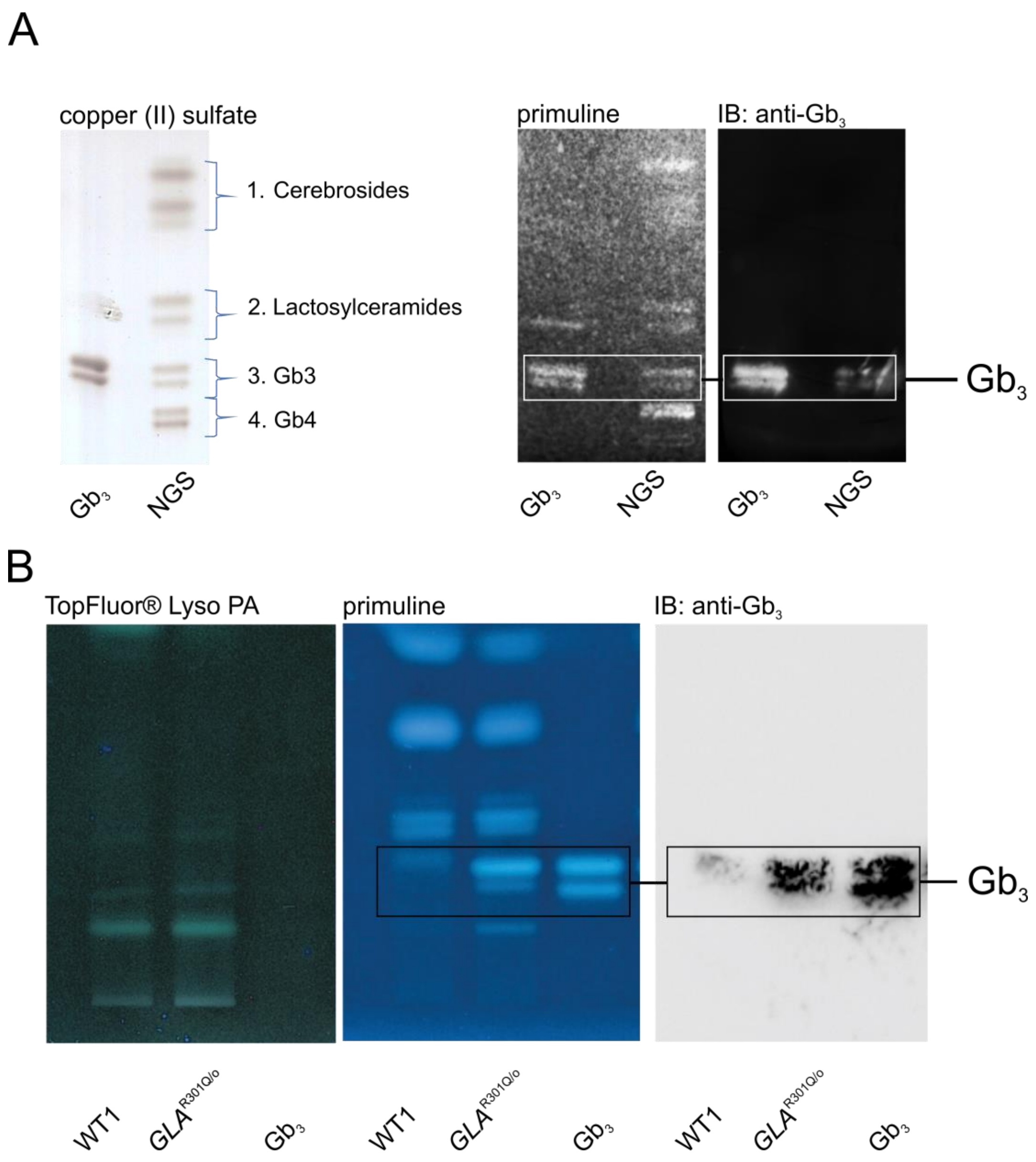

Figure S2. Far-Eastern blot shows an increased level of Gb_3 in *GLA*$^{R301Q/o}$ cells.

A. Gb_3 and a neutral glycosphingolipid mixture (NGS) were separated by TLC using a high performance thin layer chromatography (HPTLC) plate and visualized with copper (II) sulfate staining (left). The specificity of the anti-Gb_3 antibody is shown on the right side. Glycosphingolipids were sprayed with primuline reagent and made visible under UV (365nm) (middle). The same plate was used for the Far-Eastern blot. Gb_3 was specifically detected with the anti-Gb_3 antibody in both lanes (right).

B. Phospholipids were purified and separated by TLC from fibroblasts from an adult male hemizygous Fabry patients harboring the variant p.R301Q (*GLA*$^{p.R301Q/o}$) and a healthy age and sex matched donor harboring the wild type *GLA* gene (WT1). TopFluor® Lyso PA was used as an internal standard (left). With primuline reagent separated phospholipids were visualized (middle). Note that already on the primuline stained plate differences in the phospholipid profile between WT 1 and *GLA*$^{R301Q/o}$ are detectable. Far-Eastern blot

immunostained with anit-Gb_3 monoclonal antibody shows a clear increase in Gb_3 level in Fabry patient-derived fibroblasts compared to healthy control cells.

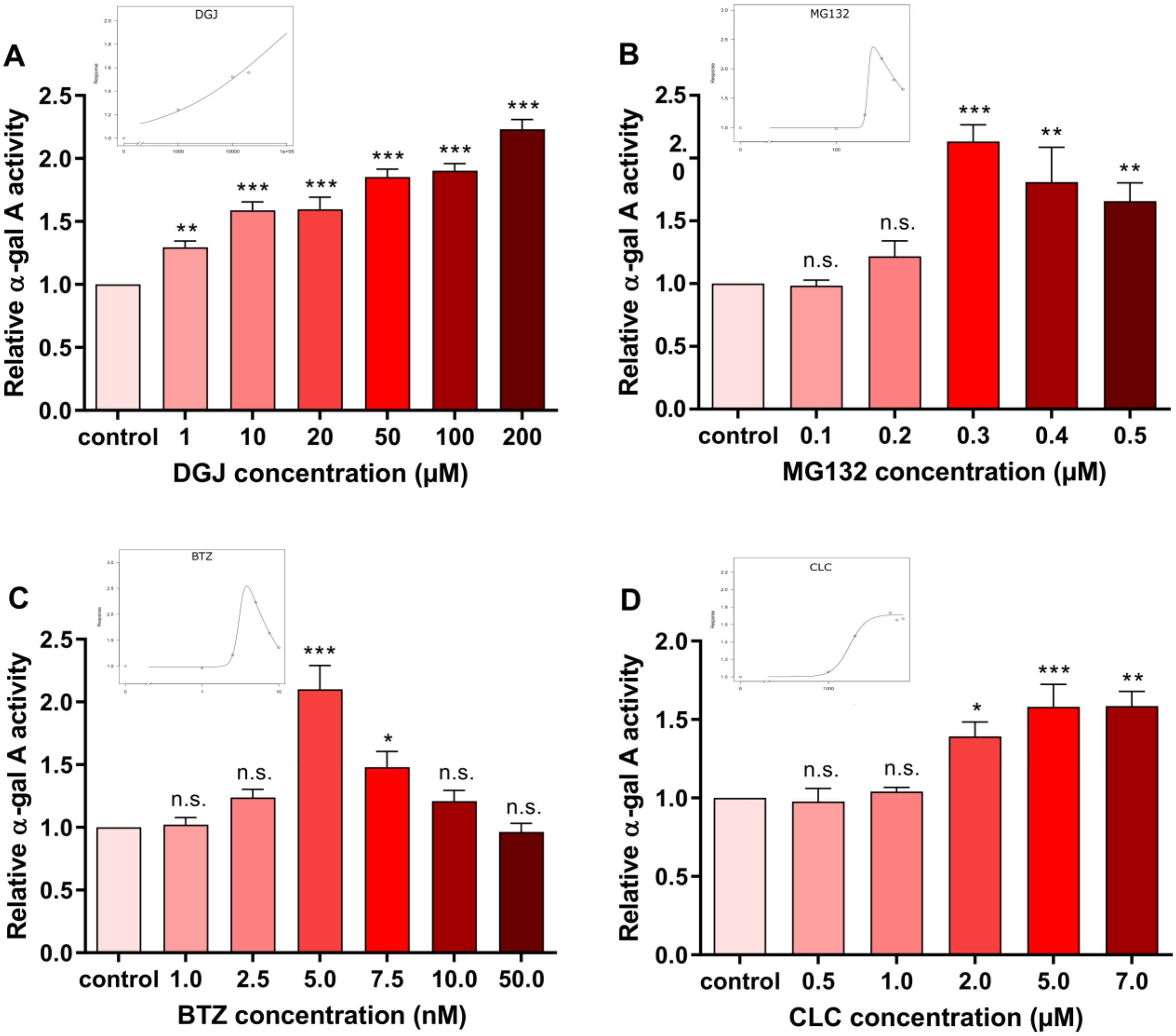

Figure S3. Enhancement of p.R301Q α-Gal A activity by proteostasis regulators. *GLA*$^{\text{p.R301Q}/\circ}$ fibroblasts were treated for 5 days with different concentrations of the pharmacological chaperone DGJ and proteostasis regulating drugs. **(A)** DGJ, **(B)** MG132, **(C)** Bortezomib **(**BTZ) and **(D)** Clasto-Lactacystin-β-lactone (CLC). The insets represent dose-response curves fitted to the fold change using non-linear regression.

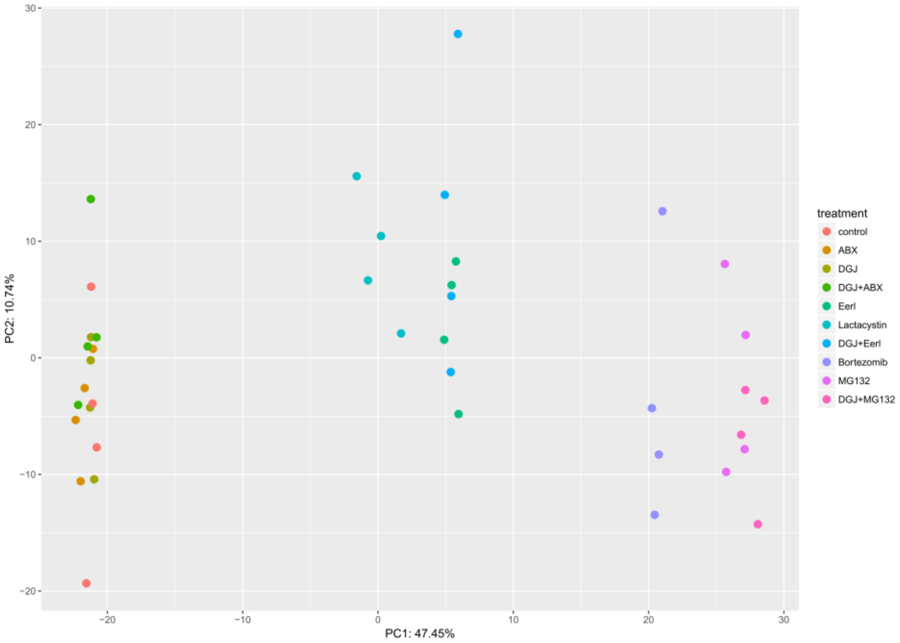

Figure S4. Principal Component Analysis on the global gene expression profiles after PR treatment of *GLA*$^{p.R301Q/\circ}$ fibroblast. PCA was performed on RMA-processed microarray samples (see Methods) using 29799 probesets. Three clusters of samples can be distinguished visually, separated along the first principal component. We conclude that MG132 and BTZ as well as the combinations of DGJ with MG132 and BTZ exert a prominent effect on the global transcriptome. DGJ applied as single treatment, however, is hardly distinguishable from untreated controls.

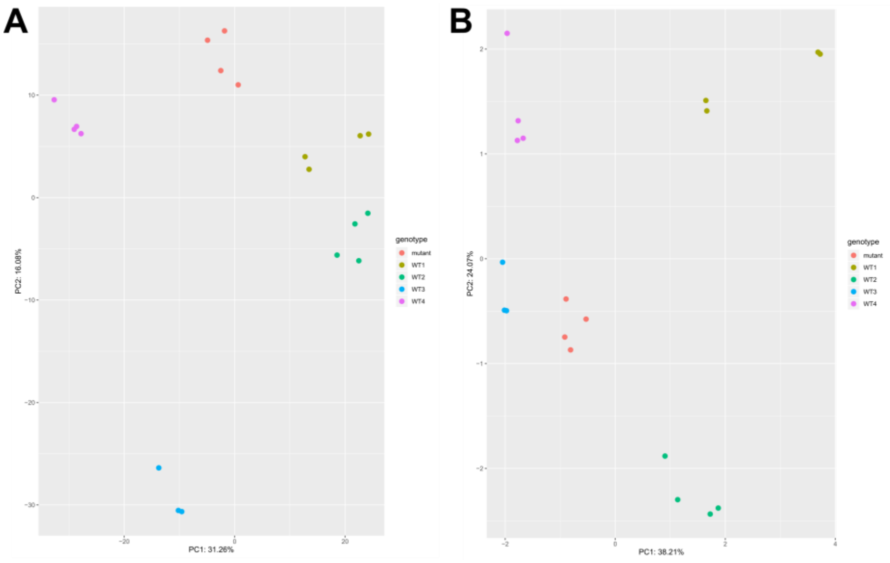

C

Gene ID	Absolute fold change
CCL2	-1.10
CREBRF	1.07
EIF2AK2	1.13
FAM129A	-1.39
FKBP5	-1.20
PPP1R15A	-1.45
PSMB8	1.12
PSMB9	1.16
PSMD2	-1.31
PSMD13	1.27
PSME1	1.25
PSME2	1.25
RNF128	-1.64
TPP1	-1.29
UBE2C	-1.45
UBE2L6	1.24
UBE2S	-1.18
UBE2T	-1.22
UGGT2	-1.24

Figure S5. Analysis on changed baseline gene expression in *GLA*$^{p.R301Q/\circ}$ fibroblasts. PCA was performed on RMA-processed microarray samples (see Methods) using (**A**) all 29799 or (**B**) the 351 proteostasis related probesets. The four replicates of the respective cell lines, WT1-4 and *GLA*$^{p.R301Q/\circ}$, clustered for both probesets. However, it could not be observed that the *GLA*$^{p.R301Q/\circ}$ FD cells stood out against the wild type lines. (**C**) Table of differentially expressed proteostasis genes. For each of the 351 proteostasis genes we determined whether it was significantly differentially expressed between the mutant *GLA*$^{p.R301Q/\circ}$ cells and any of four wild type cell lines. We computed a linear model using the genotype as categorical variable and the log2-transformed gene expression measurements as response, for each gene individually. We used Tukeys HSD method to estimate fold changes and corresponding adjusted p-values for all ten pairwise comparisons of genotypes. We selected the four comparisons involving the mutant cell line and one of the WTs. By applying a cutoff on the absolute fold change and the adjusted p-value of 1.5 and 0.05, respectively, we determined that there were 19 proteostasis genes differentially expressed in at least one WT as compared to the *GLA*$^{p.R301Q/\circ}$ cells. No gene was differentially expressed in all four WT vs. *GLA*$^{p.R301Q/\circ}$ comparisons. The colors indicate the number of WTs in which the gene was differentially expressed compared to *GLA*$^{p.R301Q/\circ}$ (1, 2 and 3).

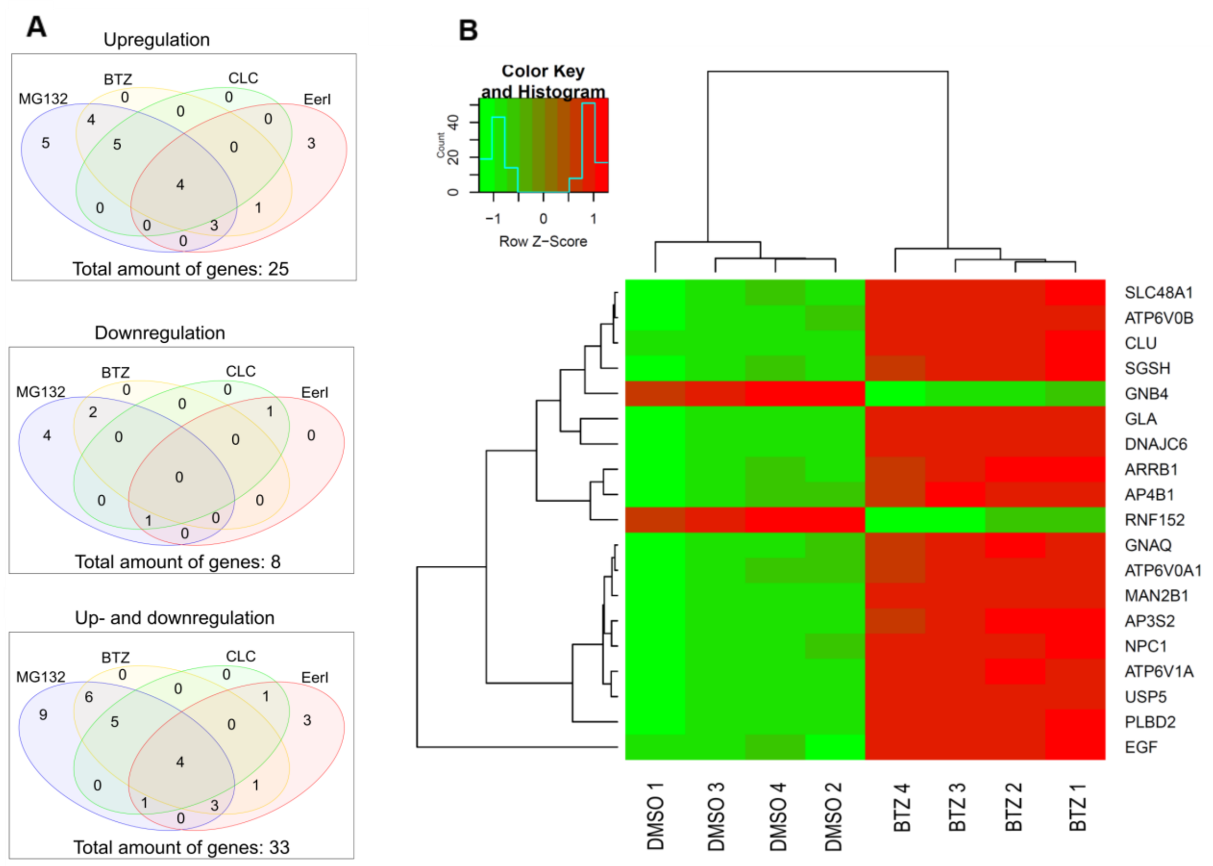

Figure S6. Transcriptional signature of lysosomal genes of PR-treated *GLA*$^{p.R301Q/o}$ fibroblasts. (A) VENN diagram of differentially expressed lysosomal genes after the treatment of *GLA*$^{p.R301Q/o}$ fibroblasts with ERAD inhibitors. A common set of 4 lysosomal genes was differentially expressed after each treatment. The majority of the genes was upregulated and only a few were downregulated. For detail, please refer to Table S10 **(B)** Heatmap of the 19 BTZ-regulated genes. Differential gene expression was defined by >1.5-fold difference with a p-value threshold of 0.05.

Table S1. Concentrations and classes of compounds used for transcriptional signatures.

Treatment	Molarity	Substance class
DMSO (control)	-	-
DGJ	50µM	PC
Bortezomib	5nM	PI
Lactacystin	5µM	PI
MG132	0,3µM	PI
Ambroxol (ABX)	40µM	PC
EerI	6µM	EI
EerI + DGJ	See above	EI +PC
MG132 + DGJ	See above	PI + PC
ABX + DGJ	See above	PC + PC

PC = pharmacological chaperone; PI = proteasomal inhibitor; EI = ERAD inhibitor

TABLE S2. Differential gene expression in *GLA* $^{p.R301Q/o}$ fibroblasts after PR treatment

Adjusted p-value < 0.05
Fold change (FC) > 1.5-fold (logFC > 0.58)

Treatment

MG132	Spalte1	Spalte2	BTZ	Spalte3	Spalte4	CLC	Spalte5	Spalte6
Gene ID	logFC	adj.P.Val	Gene ID	logFC	adj.P.Val	Gene ID	logFC	adj.P.Val
HSPA1B	3,65424298	4,96E-10	HSPA1B	3,23582349	2,28E-09	B4GALNT1	2,13607696	7,52E-08
HSPA1A	2,50429433	2,04E-09	HSPA1A	2,27139051	5,37E-09	MLLT11	2,68949274	7,52E-08
DYNC1I1	2,80099238	2,71E-09	DYNC1I1	2,74227841	5,37E-09	BLVRB	1,83030446	7,52E-08
B4GALNT1	2,30663535	7,18E-09	MLLT11	2,97611055	1,29E-08	HIST1H4H	1,50036754	7,52E-08
HSPB8	2,06858631	7,18E-09	HSPB8	1,96688972	1,29E-08	DYNC1I1	2,26668803	7,92E-08
MLLT11	3,04687904	7,18E-09	B4GALNT1	2,20963694	2,45E-08	CSRP2	-1,645875	9,07E-08
GCNT3	1,90576507	1,00E-08	GCNT3	1,85134747	3,73E-08	GLA	2,01326064	1,05E-07
ALDH2	2,01054827	1,24E-08	GLA	2,37373097	3,98E-08	ALDH2	1,91910465	1,05E-07
ZFAND2A	2,37867666	1,24E-08	ABHD4	1,93610771	4,68E-08	SEL1L3	1,91050332	1,35E-07
AKR1B10	2,13256734	1,24E-08	ALDH2	1,77287222	5,60E-08	TOP2A	-1,5785679	2,40E-07
DNAJB1	2,35632056	1,36E-08	ZFAND2A	2,04812425	6,59E-08	HSPB8	1,38989387	3,34E-07
HMOX1	1,50891736	1,36E-08	HMOX1	1,36561715	6,88E-08	ANLN	-1,4685859	4,72E-07
SEL1L3	1,95501979	2,06E-08	NRIP3	2,00835535	9,39E-08	BHMT2	1,72417205	4,89E-07
GLA	2,43571333	2,06E-08	AKR1B10	2,11947302	1,03E-07	PLK1	-1,3023222	5,32E-07
CYB5R1	1,60656619	2,74E-08	SEL1L3	1,84196832	1,03E-07	CYB5R1	1,27312232	5,48E-07
ABHD4	1,91067826	2,74E-08	CPEB1	1,49117431	1,06E-07	UBB	-0,9960317	5,55E-07
BAG3	1,61493837	2,88E-08	PSMC4	1,66308161	1,06E-07	GCNT3	1,10238886	5,55E-07
CSRP2	-1,7039181	2,88E-08	CYB5R1	1,53196323	1,08E-07	SCUBE3	-1,2031638	5,92E-07
BLVRB	1,74010584	4,56E-08	PITHD1	1,66511587	1,08E-07	NUSAP1	-1,2503049	5,92E-07
BRF2	1,38419577	4,84E-08	GSR	1,64870556	1,08E-07	SOD1	0,92733647	5,92E-07
LRP8	1,54438016	6,38E-08	ME1	1,8393325	1,08E-07	ME1	1,70051955	5,97E-07
NRIP3	2,21410392	7,63E-08	LRP8	1,66798756	1,09E-07	LCE2A	2,38343374	6,20E-07
PITHD1	1,68224695	8,37E-08	PCYT1B	1,2650418	1,51E-07	KIF20A	-1,3296188	6,20E-07
MKI67	-1,9590813	8,37E-08	BLVRB	1,50249017	1,51E-07	LRP8	1,28508072	7,18E-07
CPEB1	1,44652961	8,37E-08	FAM219A	1,85676837	1,52E-07	TIMP4	1,77010239	1,07E-06
TPX2	-1,6460198	8,37E-08	GK	2,10609631	1,72E-07	MXRA5	-1,0816444	1,40E-06
OSGIN1	1,18625929	8,37E-08	EPS15L1	1,56016139	1,78E-07	CASC5	-1,4980796	1,42E-06
GSR	1,50630339	8,37E-08	GPX3	1,49947243	1,78E-07	PBK	-1,2881615	1,42E-06
PSMC4	1,6043547	8,37E-08	BSDC1	1,35793621	1,78E-07	DLGAP5	-1,6404885	1,42E-06
PRLR	-1,8380984	8,37E-08	USP5	1,38514161	1,93E-07	KPRP	1,37118297	1,42E-06
CLU	1,17001922	8,37E-08	PSMD11	1,39322665	1,93E-07	PSMB6	0,93244183	1,42E-06
BSDC1	1,30726064	8,37E-08	SOX4	-1,3695	1,93E-07	USP5	1,17755059	1,42E-06
FAM219A	1,84834779	8,37E-08	MAPRE3	1,23353389	2,27E-07	SESN3	-1,4660266	1,43E-06
FST	-1,6448089	8,37E-08	DNAJB1	1,60644489	2,32E-07	PTGR1	1,00655292	1,53E-06
BHMT2	1,94042975	8,37E-08	BHMT2	1,80295787	2,68E-07	TMEM161A	0,94209605	1,55E-06
ME1	1,9242753	8,37E-08	CSRP2	-1,4881583	3,03E-07	GPX3	1,27449857	1,74E-06
TIMP4	2,17840897	8,37E-08	FGF10	-1,8958692	3,11E-07	PSMC4	1,28291486	1,82E-06
ABHD3	1,98501329	8,37E-08	DNAJC6	1,57108326	3,17E-07	MKI67	-1,7435296	1,82E-06
SOD1	1,27893169	8,37E-08	OSGIN1	1,34595156	3,17E-07	C16orf93	1,0172894	1,82E-06
USP5	1,39763456	8,78E-08	BRF2	1,38460217	3,23E-07	AKR1B10	1,4728539	1,82E-06
MAPRE3	1,36827324	8,78E-08	DAP3	1,3976089	3,30E-07	CDKN3	-0,9145611	1,82E-06
HSPH1	1,47852714	8,78E-08	PSMD13	1,26798242	3,30E-07	ANGPTL1	1,09170366	1,85E-06
LGALS8	1,28991799	8,78E-08	BAG3	1,17253243	3,46E-07	PSMD14	0,98389685	1,85E-06
BUB1	-1,7581382	8,80E-08	UFD1L	1,32187303	3,46E-07	AKR1B1	0,79318436	1,85E-06
DNAJC6	1,54905252	9,73E-08	GCLM	1,46215894	3,46E-07	CCRL1	-0,9436643	1,85E-06
TOP2A	-2,0183143	9,75E-08	PSMD3	1,35124139	3,46E-07	PRC1	-1,2278888	1,85E-06
EPS15L1	1,56745545	9,75E-08	LGALS8	1,260701	3,46E-07	PSMB3	1,36445695	2,02E-06
GPX3	1,52063517	1,04E-07	LOC344887	1,72701371	3,55E-07	ACOT13	0,95365538	2,08E-06
HMGB2	-1,2782299	1,04E-07	NQO2	1,0213516	3,59E-07	PRR11	-1,235916	2,08E-06
ANLN	-1,7387941	1,08E-07	MYBL1	-1,0087992	3,59E-07	PIR	1,00616922	2,08E-06
WFS1	1,32660457	1,09E-07	TIMP4	2,02671366	3,67E-07	UFD1L	0,9909193	2,17E-06
STOX2	1,57465707	1,09E-07	PSMD14	1,18496851	3,68E-07	NRIP3	1,53329125	2,17E-06
GK	2,10742736	1,13E-07	SNX10	1,42710332	3,68E-07	KIF11	-1,1471999	2,17E-06
KIAA1199	-1,2227278	1,13E-07	PSMA5	1,15272732	3,68E-07	PFN2	0,95035617	2,17E-06
DKK2	-1,6767558	1,13E-07	SOD1	1,05629859	3,68E-07	SCG5	-0,7570897	2,17E-06
KIF20A	-1,5147259	1,20E-07	WFS1	1,23099079	3,76E-07	PSMD13	0,94398493	2,22E-06
CCNA2	-2,0517681	1,27E-07	OSR2	-1,1367838	3,85E-07	ROBO2	-1,0782954	2,26E-06
PARM1	1,17466813	1,33E-07	FST	-1,2522026	3,98E-07	TPX2	-1,0972349	2,26E-06
UFD1L	1,36003643	1,46E-07	PSMD6	1,12966825	4,10E-07	AURKA	-0,8621577	2,27E-06
PCYT1B	1,17248837	1,62E-07	PSMB3	1,43976579	4,12E-07	SOX4	-0,8450949	2,34E-06
HSPD1	1,19251901	1,62E-07	ABHD3	1,82185545	4,20E-07	PSMB7	0,80376039	2,34E-06
KRTAP1-5	-1,8398897	1,68E-07	HSPH1	1,11056363	4,26E-07	BUB1	-1,1976495	2,34E-06
CASC5	-1,7968519	1,81E-07	DKK2	-1,4872625	4,50E-07	LCE2C	1,29008544	2,35E-06
ITGB8	-1,5130935	1,81E-07	KBTBD8	1,81000535	4,50E-07	UCHL1	0,93532232	2,35E-06
DAP3	1,37500703	1,86E-07	PSMB2	1,15788675	4,75E-07	BSCL2	0,73192659	2,35E-06
AURKA	-1,1913533	1,92E-07	PGD	0,97801325	4,75E-07	GPC4	-1,211417	2,37E-06
PTGR1	1,29115264	1,92E-07	LMCD1	-1,0491532	4,75E-07	HIST1H2BM	-1,0631064	2,37E-06
MAP1A	1,24111893	1,97E-07	PSMB4	1,12228999	5,11E-07	HIST1H3J	-1,1583443	2,37E-06
SNX10	1,37824883	1,97E-07	PTGR1	1,15461334	5,69E-07	SEMA3D	-0,9183021	2,37E-06
PSMD14	1,14025606	1,97E-07	NSFL1C	0,97885067	5,69E-07	NIPAL3	0,98373483	2,48E-06
DLGAP5	-1,9630087	2,11E-07	LOC1001290	-1,6976869	5,96E-07	NDRG3	0,90311815	2,55E-06
CDCA8	-1,0980019	2,12E-07	TNFSF9	1,49186138	6,38E-07	HIST1H3B	-1,2029478	2,55E-06
AKR1B1	1,04669248	2,12E-07	PSMA3	1,28186624	6,38E-07	RDH10	-0,803647	2,55E-06
FKBP4	1,11004295	2,12E-07	DESI1	1,19910377	6,55E-07	NIT2	1,04944536	2,61E-06
NQO2	1,04551048	2,12E-07	CTH	1,53429324	6,55E-07	PSMD11	0,9472431	2,61E-06

EerI	Spalte7	Spalte8	ABX	Spalte9	Spalte10	DGJ	Spalte11	Spalte12
Gene ID	logFC	adj.P.Val	Gene ID	logFC	adj.P.Val	Gene ID	logFC	adj.P.Val
HMOX1	2,12435808	1,18E-08	EGR1	0,93629396	0,00040282			
MKI67	-2,7557713	3,75E-08	SNORA38B	-0,744975	0,04422907			
TOP2A	-2,3238413	4,39E-08						
ANLN	-2,237975	4,86E-08						
DYNC1I1	1,76737246	4,86E-08						
HSPB8	1,65344974	5,13E-08						
MLLT11	2,36407209	6,02E-08						
DKK2	-1,4220253	1,01E-07						
KIF20A	-1,6094215	1,01E-07						
B4GALNT1	1,71920002	1,16E-07						
NUSAP1	-1,8937576	1,16E-07						
SEL1L3	1,56071064	1,24E-07						
CASC5	-2,1002283	1,24E-07						
CCNA2	-1,4641164	1,24E-07						
BUB1	-1,8611244	1,24E-07						
PRC1	-1,6651997	1,24E-07						
TPX2	-1,7242588	1,24E-07						
NDC80	-1,9921873	1,24E-07						
ROBO2	-1,5812461	1,54E-07						
TTK	-1,4590202	1,54E-07						
RRM2	-1,7929437	1,93E-07						
CKAP2L	-1,8290832	2,01E-07						
GCNT3	1,155304	2,01E-07						
HIST1H3J	-1,4373119	2,01E-07						
NCAPG	-2,020851	2,01E-07						
GCLM	1,59734496	2,01E-07						
PBK	-1,8926056	2,04E-07						
HJURP	-1,2906918	2,42E-07						
GK	1,58607196	2,46E-07						
TYMS	-1,443109	2,47E-07						
IQGAP3	-1,236191	3,11E-07						
PLK1	-1,484978	3,21E-07						
AURKA	-1,0825353	3,43E-07						
HIST1H3F	-1,6323046	3,43E-07						
DLGAP5	-2,0176102	3,43E-07						
KIF23	-1,2739728	3,83E-07						
ALDH2	1,21190133	3,83E-07						
KIF11	-1,6959299	3,83E-07						
LRP8	1,25361136	3,83E-07						
PGD	0,97335141	3,83E-07						
HSPA1B	1,22429501	3,83E-07						
HIST1H3B	-1,8237413	3,83E-07						
HSPA1A	0,9542073	3,83E-07						
SGOL1	-1,3808209	3,83E-07						
CDC20	-1,0133491	3,83E-07						
GLA	1,48557181	3,83E-07						
ESCO2	-2,0721876	3,83E-07						
CYB5R1	1,0550481	4,42E-07						
KIF2C	-1,3175257	4,52E-07						
CCNB2	-1,4003047	4,52E-07						
AKR1C2	1,07730499	4,52E-07						
CDCA8	-1,08602	4,55E-07						
ABHD4	1,26740254	4,55E-07						
HIST1H2AH	-1,1127528	4,55E-07						
ME1	1,48607997	4,55E-07						
ASPM	-2,2454409	4,55E-07						
KIF15	-1,3148843	4,58E-07						
BRIP1	-1,8624712	4,88E-07						
OXTR	-0,8832359	4,90E-07						
BHMT2	1,37905035	5,14E-07						
MXRA5	-1,2303091	5,18E-07						
CDKN3	-1,0053735	5,18E-07						
DHCR24	-0,9427151	5,18E-07						
FAM111B	-2,0367529	5,18E-07						
PRR11	-1,8143328	5,18E-07						
IGFBP5	-0,8805337	5,29E-07						
SHCBP1	-1,8409842	5,31E-07						
PSMD11	1,03489784	5,55E-07						
PSMD14	0,96213423	6,27E-07						
BLVRB	1,0835166	6,27E-07						
MCM5	-0,8920672	6,82E-07						
NCAPH	-1,23675	6,90E-07						
POPDC3	1,31212926	6,90E-07						
TIMP4	1,23383238	6,99E-07						
MSC	0,88528043	7,10E-07						

MG132+DGJ	Spalte13	Spalte14	EerI+DGJ			ABX+DGJ		
Gene ID	logFC	adj.P.Val	Gene ID	logFC	adj.P.Val	Gene ID	logFC	adj.P.Val
HSPA1B	3,66452703	4,58E-10	HMOX1	2,11303292	1,55E-08	EGR1	0,63034643	0,00382659
HSPA1A	2,50841077	1,42E-09	MLLT11	2,32981803	9,37E-08	SNORD113-3	-0,8991384	0,01358631
DYNC1I1	2,91224102	2,45E-09	DYNC1I1	1,73405533	9,91E-08	RNA5SP268	-0,7417025	0,02565024
MLLT11	3,0209789	8,02E-09	HSPB8	1,628001	1,93E-07	SNORA38B	-0,90658	0,0294883
SEL1L3	2,10430232	9,84E-09	ANLN	-2,1564473	1,93E-07	OTTHUMG0	-0,7497869	0,0294883
HSPB8	2,13070726	9,84E-09	RRM2	-1,7142601	2,73E-07	MIR604	-0,6536757	0,0294883
GCNT3	2,12776228	1,02E-08	B4GALNT1	1,64814885	2,73E-07	MIR21	-0,7419533	0,03487452
ZFAND2A	2,34214173	1,07E-08	DKK2	-1,3415596	2,73E-07	LOC1001909	-0,7290114	0,03824131
CCNA2	-1,9021061	1,90E-08	TOP2A	-2,2964605	2,73E-07	OTTHUMG0	-0,8130533	0,04657497
GLA	2,51420861	1,90E-08	KIF20A	-1,4945474	2,73E-07	OTTHUMG0	-0,589538	0,04657497
ANGPTL1	1,66491904	1,90E-08	HIST1H3B	-1,6534346	2,73E-07			
PRLR	-2,0156106	1,90E-08	ROBO2	-1,5814657	3,36E-07			
B4GALNT1	2,38163442	1,90E-08	PBK	-1,8049616	3,38E-07			
CPEB1	1,53287327	2,56E-08	SEL1L3	1,52258688	5,06E-07			
KBTBD8	1,97790744	2,65E-08	GCLM	1,54188048	5,09E-07			
AKR1B10	2,29743978	2,65E-08	PLK1	-1,3568188	5,33E-07			
ABHD4	1,95439391	2,65E-08	MKI67	-2,9998718	5,40E-07			
GK	2,23566026	2,65E-08	HJURP	-1,2178554	5,40E-07			
NRIP3	2,30942487	2,89E-08	ALDH2	1,25033965	5,77E-07			
DNAJB1	2,31279587	2,89E-08	GK	1,59758779	6,34E-07			
KRTAP1-5	-1,7273775	3,27E-08	CKAP2L	-1,8271304	6,34E-07			
BAG3	1,62867985	3,36E-08	KIF2C	-1,2489247	6,34E-07			
CSRP2	-1,6423849	3,39E-08	TTK	-1,3851517	6,36E-07			
CYB5R1	1,73263999	3,42E-08	PRR11	-1,7770819	6,48E-07			
BHMT2	2,01624423	3,44E-08	ME1	1,58957313	6,69E-07			
FST	-1,5835759	3,56E-08	BUB1	-1,7441783	6,69E-07			
KIAA1199	-1,3104404	3,56E-08	NCAPG	-1,984945	6,69E-07			
TIMP4	2,1554148	3,56E-08	IQGAP3	-1,2666446	6,94E-07			
HSPH1	1,49110456	3,78E-08	PGD	1,03825016	6,94E-07			
TTK	-1,4394349	3,92E-08	GLA	1,59177083	7,65E-07			
ALDH2	2,08376594	4,42E-08	NUSAP1	-1,7336184	7,69E-07			
ME1	1,97701921	4,42E-08	TYMS	-1,3478354	7,69E-07			
ANLN	-1,6286747	4,94E-08	PRC1	-1,7088466	7,69E-07			
ABHD3	2,12363727	4,94E-08	ESCO2	-1,9837348	7,92E-07			
SNX10	1,44522828	4,94E-08	PFN2	1,02403767	8,49E-07			
PSMD6	1,22777178	4,94E-08	ABHD4	1,21548051	8,49E-07			
TOP2A	-1,8572581	5,62E-08	CYB5R1	1,1186467	8,70E-07			
PITHD1	1,74503345	5,62E-08	CASC5	-2,0755564	9,77E-07			
BLVRB	1,82888318	5,62E-08	GSR	1,15681523	1,04E-06			
SOX4	-1,4925342	5,63E-08	POPDC3	1,42313749	1,04E-06			
SOD1	1,32888832	5,82E-08	TPX2	-1,722612	1,04E-06			
MAPRE3	1,36894589	6,01E-08	PSMD11	1,05327616	1,05E-06			
NQO2	1,11920769	6,10E-08	ASPM	-2,3713788	1,08E-06			
STOX2	1,71165007	6,10E-08	TIMP4	1,22829882	1,10E-06			
DKK2	-1,5899941	6,20E-08	FOXM1	-1,2558833	1,16E-06			
TPX2	-1,5624024	6,29E-08	GCNT3	1,13814992	1,23E-06			
LRP8	1,48733469	6,29E-08	SLC7A11	1,33890299	1,23E-06			
STC1	-1,3779474	6,29E-08	BHMT2	1,34687422	1,29E-06			
AHSA1	1,21327262	6,29E-08	E2F8	-1,3592471	1,29E-06			
HAS2	-1,201223	6,29E-08	OXTR	-0,9896266	1,29E-06			
DAP3	1,432449	6,29E-08	DIAPH3	-1,5883121	1,29E-06			
PTGR1	1,36371401	6,29E-08	NDC80	-1,8396954	1,31E-06			
DMGDH	1,32926034	6,29E-08	FAM111B	-2,0032556	1,31E-06			
PLK1	-1,5044185	6,29E-08	DLGAP5	-1,8638335	1,31E-06			
PCYT1B	1,30217954	6,29E-08	SGOL1	-1,3322952	1,32E-06			
UFD1L	1,44062247	6,29E-08	NCAPH	-1,2569162	1,41E-06			
CLU	1,25668727	6,41E-08	CPEB1	0,93829106	1,47E-06			
BUB1	-1,6201191	6,52E-08	PSMC4	1,12664966	1,56E-06			
USP5	1,45199984	6,65E-08	SHCBP1	-1,8266106	1,57E-06			
CTH	1,44525444	6,65E-08	LMNB1	-1,4282154	1,60E-06			
EPHX4	1,63753103	7,00E-08	LOC344887	1,32527669	1,60E-06			
LGALS8	1,29088478	7,20E-08	ENPP1	-1,0259816	1,62E-06			
DNAJC6	1,54459555	7,33E-08	AKR1C1	1,08442394	1,62E-06			
LBH	-1,1106176	7,33E-08	MCM5	-0,9065454	1,62E-06			
C9orf72	1,69271187	7,53E-08	TXNRD1	0,89099819	1,71E-06			
FAM219A	1,83773954	7,64E-08	AKR1C2	1,08814567	1,71E-06			
PARM1	1,39488574	7,66E-08	SPP1	1,31789885	1,75E-06			
HMOX1	1,42714046	8,00E-08	HTRA3	1,03603431	1,75E-06			
GPX3	1,53230856	8,10E-08	CLSPN	-1,8597764	1,75E-06			
LOC100129034	-1,6455331	8,37E-08	ITGB8	-1,1333157	1,75E-06			
ASPM	-2,1152381	8,37E-08	CDCA8	-1,039521	1,86E-06			
HMGB2	-1,2317829	8,43E-08	PSMD3	1,04104981	1,87E-06			
PSMA3	1,34932385	8,97E-08	HIST1H1B	-1,5509892	1,94E-06			
KIF20A	-1,3452297	9,17E-08	HMMR	-1,3729014	1,94E-06			
PSMD14	1,19653617	9,53E-08	LRP8	1,13420939	1,94E-06			

TTK	-1,540505	2,12E-07	MAP1A	1,13190011	6,66E-07	NDC80	-1,2930126	2,61E-06	CLSPN	-1,7579863	7,34E-07
PSMD6	1,13538261	2,12E-07	TTLL7	1,53569402	6,73E-07	SERPINB10	0,74872806	2,65E-06	GSR	1,09729847	7,34E-07
SGOL1	-1,4522012	2,12E-07	ACO2	0,98368418	7,16E-07	EML5	-1,3115988	2,74E-06	CPEB1	0,96610363	7,60E-07
PSMB4	1,13886853	2,12E-07	ATL1	1,34838635	7,38E-07	LBH	-0,7015968	2,81E-06	LMNB1	-1,456936	7,60E-07
NSFL1C	0,97641101	2,12E-07	ITGB8	-1,3514746	7,38E-07	APOBEC3B	-0,7242695	3,08E-06	CENPF	-1,5267974	7,91E-07
SOX4	-1,3785464	2,12E-07	NOG	-1,0761195	7,38E-07	GSTM5	0,97097642	3,08E-06	NUF2	-1,5673085	7,92E-07
AHSA1	1,13268456	2,14E-07	HTR2B	-1,1186869	7,38E-07	ASPM	-1,3274379	3,08E-06	AKR1C1	1,05721141	7,92E-07
ASPM	-2,2737279	2,14E-07	ADRM1	1,08262797	7,38E-07	FGF10	-1,3093031	3,14E-06	DEPDC1	-1,4492223	7,95E-07
CDC20	-1,0420267	2,16E-07	TMEM161A	0,91562625	7,45E-07	CCNB2	-1,0405341	3,35E-06	PFN2	0,91068257	8,23E-07
PRR11	-1,7091485	2,18E-07	PRLR	-1,4848689	7,58E-07	EGR1	1,25606773	3,43E-06	CDCA2	-1,2811088	8,23E-07
NDC80	-1,8812299	2,21E-07	SORD	1,09338815	7,88E-07	DMGDH	1,06979372	3,43E-06	TXNRD1	0,88626889	8,23E-07
KBTBD8	1,80607799	2,38E-07	ATP6V1A	1,12801247	7,88E-07	PSMC5	0,83512453	3,44E-06	CEP55	-1,6047527	8,51E-07
PSMD13	1,22221467	2,38E-07	IFI44	2,01225108	8,24E-07	PSMD3	0,99139399	3,44E-06	HMMR	-1,4456956	8,51E-07
LOC1001290	-1,7014882	2,40E-07	PSMA6	0,94014064	8,86E-07	SPP1	1,20447357	3,44E-06	CDK1	-1,8834183	8,51E-07
ACO2	1,07218448	2,42E-07	USP14	1,09024547	9,20E-07	PSMB4	0,93932022	3,44E-06	DIAPH3	-1,5443645	8,89E-07
STIP1	1,17159354	2,42E-07	GSTM5	1,15297788	9,26E-07	HAS2	0,80377071	3,44E-06	HTRA3	1,08263882	8,89E-07
PSMB6	0,97120387	2,57E-07	NXPE3	1,29542135	9,38E-07	FAM65B	-0,8597706	3,61E-06	ITGB3	1,26443341	9,03E-07
FGF10	-2,0042563	2,61E-07	PSMB6	0,97302192	9,61E-07	FABP3	-1,1086145	3,64E-06	TCF19	-1,1094757	9,03E-07
PRC1	-1,501456	2,65E-07	BLOC1S2	1,01701868	9,67E-07	TNS3	-0,7878729	3,67E-06	FANCD2	-1,2171185	9,71E-07
HAS2	-1,1585395	2,66E-07	UBR3	1,23834552	9,74E-07	RIPK3	1,13912768	3,67E-06	FANCI	-1,3012214	9,71E-07
PSMD11	1,29427652	2,84E-07	MKI67	-1,5137884	1,00E-06	ACO2	0,76936091	3,67E-06	MCM10	-1,0219783	1,04E-06
MYBL1	-1,0657042	2,84E-07	FAM129A	1,28610325	1,05E-06	KIAA1199	-0,6627488	3,67E-06	ENPP1	-0,9318672	1,04E-06
SORD	1,1288371	2,89E-07	PSMD1	1,02997618	1,07E-06	SNX10	0,69894487	3,67E-06	KIF4A	-1,4895524	1,04E-06
TMEM161A	1,06135328	2,95E-07	PSMD4	0,89880047	1,07E-06	FYB	0,88619939	3,67E-06	APOBEC3B	-0,8354508	1,05E-06
LMO7	-1,0803191	2,95E-07	STOX2	1,49252538	1,09E-06	HIST1H2AE	-1,5208465	3,67E-06	PIR	1,14892459	1,05E-06
CLPB	1,07309933	2,96E-07	HMGB2	-1,1720727	1,11E-06	ADRM1	0,95972706	3,67E-06	MMP3	0,75549717	1,11E-06
MEOX2	-1,5160737	2,96E-07	PIR	1,12725485	1,15E-06	MAPRE3	0,713955	3,67E-06	PAK6	-1,0388635	1,11E-06
CENPF	-1,6248911	3,05E-07	CLU	0,8471477	1,18E-06	AIFM2	0,66885744	3,67E-06	PSMA3	0,99186446	1,11E-06
CAMK2G	0,9137442	3,18E-07	KIAA1199	-0,9328525	1,21E-06	IQGAP3	-0,957173	3,67E-06	FAM219A	1,25761306	1,15E-06
DMGDH	1,25802975	3,23E-07	CLPB	1,11928829	1,22E-06	PLBD2	0,73221781	3,67E-06	DHCR7	-1,0085956	1,21E-06
PSMD3	1,24972192	3,24E-07	KRTAP1-5	-1,6213703	1,24E-06	PLXDC2	-0,6949006	3,67E-06	TRIM16L	0,80119172	1,21E-06
IQGAP3	-1,0547689	3,28E-07	CAMK2G	0,91954127	1,28E-06	ANKRD1	-0,964599	3,67E-06	HIST1H2AB	-1,8949946	1,21E-06
DYNLL2	1,14440627	3,28E-07	PLAA	1,201214	1,29E-06	ENPP1	-0,8008555	3,67E-06	PSMC2	0,81426451	1,23E-06
FAM65B	-1,1622043	3,35E-07	DOLPP1	1,14184509	1,34E-06	ABHD4	0,98258249	3,67E-06	ADRM1	0,98004656	1,26E-06
NUSAP1	-1,3644564	3,41E-07	CDC20	-0,862536	1,35E-06	DAP3	0,92387839	3,67E-06	NSFL1C	0,78013163	1,27E-06
LMCD1	-1,2072611	3,45E-07	PSMA4	1,05979716	1,42E-06	SMIM10	0,7880705	3,71E-06	HIST1H2BM	-1,431623	1,27E-06
TTLL7	1,46438853	3,45E-07	PSMB7	0,92073012	1,45E-06	NTM	-0,6218455	3,73E-06	CCDC15	-0,827614	1,27E-06
TTC4	0,93690002	3,49E-07	EPHX4	1,4278698	1,45E-06	RNF150	-0,8880798	3,73E-06	SQRDL	0,88275186	1,29E-06
PSMA3	1,25880447	3,49E-07	SNX21	0,82725158	1,46E-06	FAM129A	0,99899262	3,97E-06	TRIM16	0,78417223	1,29E-06
MSTN	-1,0505173	3,49E-07	CCDC134	0,84267992	1,51E-06	HIST1H2AH	-0,8730311	3,97E-06	SKA1	-1,4738726	1,33E-06
CKAP2L	-1,4398826	3,49E-07	PSMD12	1,22223891	1,51E-06	PLXNC1	-0,6348808	4,10E-06	SLC7A11	1,3287351	1,36E-06
HJURP	-1,0950275	3,49E-07	KLHL21	0,79726093	1,52E-06	TNFSF13B	0,86116372	4,10E-06	HIST1H2BI	-1,6903068	1,37E-06
BMPER	-1,2103338	3,50E-07	AIFM2	0,9085767	1,52E-06	LCE1F	0,98813821	4,27E-06	TMEM57	1,0794808	1,42E-06
ESCO2	-1,53133	3,50E-07	NEK6	-0,789492	1,52E-06	CLEC2A	-0,7089374	4,42E-06	PSMC4	1,05607111	1,42E-06
KIF18A	-1,6216917	3,50E-07	TXNL1	0,83715063	1,56E-06	TM4SF1	-0,7127884	4,62E-06	PSMD13	0,87309783	1,49E-06
WEE1	-1,4809891	3,56E-07	OTTHUMG0	0,90492452	1,56E-06	CCNB1	-0,7330322	4,63E-06	C14orf1	-0,7859538	1,49E-06
TNFRSF19	-1,3545759	3,56E-07	HSP90AA1	0,7440069	1,57E-06	ITGA11	-0,6826449	4,91E-06	PSMD3	1,03196398	1,50E-06
KIF15	-1,2170423	3,72E-07	PCYT1A	1,04281041	1,58E-06	CTHRC1	-0,627129	4,94E-06	TENM2	-0,9068875	1,51E-06
ATL1	1,2487395	3,72E-07	TXNIP	-1,2721076	1,58E-06	ADAMTS1	0,79825432	5,20E-06	TTC4	0,89048302	1,51E-06
TNFSF13B	1,07093607	3,72E-07	TALDO1	0,98398571	1,62E-06	RRM2	-0,8436323	5,43E-06	KIAA0101	-1,3675203	1,53E-06
KIF2C	-1,2074727	3,72E-07	POPDC3	1,11890958	1,62E-06	DEPDC1	-1,0323959	5,44E-06	E2F8	-1,3638516	1,53E-06
FAM111B	-1,9879002	3,72E-07	OTTHUMG0	1,96664722	1,64E-06	WFS1	0,82226522	5,44E-06	UFD1L	0,88477395	1,55E-06
STC1	-1,2666847	3,72E-07	PPP2R2C	1,04866277	1,64E-06	OTTHUMG0	0,94420755	5,44E-06	MLF1IP	-1,4282989	1,55E-06
DNTTIP2	0,96701614	3,77E-07	MX1	1,14445889	1,64E-06	HMMR	-1,1369404	5,44E-06	AIFM2	0,74162051	1,57E-06
FAM129A	1,3429708	3,77E-07	AKR1B15	0,935149	1,65E-06	AKR1C1	0,79226443	5,44E-06	PTGR1	0,86365988	1,57E-06
PSMB7	0,97718696	3,77E-07	CCNA2	-1,7134732	1,72E-06	GPI	0,68893953	5,44E-06	HMGB2	-0,9660285	1,60E-06
LBH	-1,0903901	3,77E-07	KIAA0930	0,75469358	1,73E-06	BABAM1	0,7926155	5,44E-06	BSDC1	0,81984627	1,68E-06
PLK1	-1,6394167	3,82E-07	DNAJC2	1,17944633	1,73E-06	PSMA7	0,63566048	5,44E-06	SEMA3D	-0,9706628	1,69E-06
LMNB1	-1,5018946	3,85E-07	STIP1	0,98027732	1,73E-06	CDK1	-1,2493005	5,51E-06	NQO1	0,7065659	1,75E-06
SCG5	-0,9058672	3,88E-07	NDUFAB1	0,86164219	1,76E-06	KIAA0930	0,63932655	5,58E-06	ACAT2	-0,958859	1,77E-06
KCNQ5	-1,2627883	3,95E-07	SDE2	1,29857843	1,76E-06	PSMA1	0,79800445	5,82E-06	PSMA5	0,82016286	1,77E-06
PSMD4	0,9081247	4,00E-07	VEZT	1,02076548	1,76E-06	ARHGAP11A	-1,0204947	5,94E-06	COL12A1	-0,7666736	1,77E-06
PPAP2B	-1,0618342	4,00E-07	UBE2O	0,87356218	1,86E-06	HIST1H3F	-1,398786	5,95E-06	DAP3	0,84714584	1,77E-06
HIST1H3F	-1,5144203	4,00E-07	E2F8	-1,1045033	1,86E-06	PSMB2	0,88991465	5,95E-06	AKR1B10	1,11814953	1,84E-06
DESI1	1,2384314	4,10E-07	IDE	0,9739112	1,86E-06	SQRDL	0,79672839	5,95E-06	ITGB8	-1,0344941	1,95E-06
FAM102A	1,00612134	4,14E-07	ACOT13	0,96558969	1,86E-06	NSFL1C	0,63710717	5,95E-06	ABCB6	0,67785437	2,01E-06
NPC1	1,19398574	4,23E-07	PSMC2	0,88742509	1,88E-06	TP53INP1	-1,0501303	5,99E-06	SPP1	1,23139469	2,06E-06
KIF23	-1,3675958	4,25E-07	TM2D2	1,14245433	1,89E-06	PSMA3	0,8669182	6,07E-06	SLC3A2	0,77252836	2,09E-06
DEPDC1	-1,540696	4,39E-07	FHOD1	0,81202727	1,90E-06	NUF2	-1,0675134	6,27E-06	GTSE1	-0,9160531	2,09E-06
AIMP1	-0,9888704	4,42E-07	PSMC5	1,0426802	1,97E-06	HIST1H2BI	-1,4818175	6,32E-06	RAD54L	-0,7013837	2,09E-06
MYLK	-0,9724888	4,66E-07	MXRA5	-0,7870018	1,97E-06	STEAP1B	0,65466552	6,38E-06	DUSP5	0,77060549	2,10E-06
PGD	0,91962703	4,73E-07	AKR1C1	0,90652717	1,98E-06	CADM1	-0,6959834	6,46E-06	ATAD2	-1,1869104	2,10E-06
E2F8	-1,2456537	4,74E-07	INTS10	1,1573035	1,98E-06	PGD	0,64834854	6,49E-06	MYBL2	-0,7631922	2,10E-06
BLOC1S2	0,9262807	4,91E-07	HSP90AB3P	0,90636423	1,98E-06	TMEM97	-0,6606179	6,62E-06	CDC20P1	-1,1729823	2,10E-06
ATP6V1A	1,16471418	4,97E-07	RTN3	0,97577552	1,98E-06	HIST1H2BF	-1,2292416	6,69E-06	SOD1	0,6751669	2,10E-06
AIFM2	0,84225522	5,02E-07	NIT2	1,05594154	1,98E-06	PPP2R2C	0,93029921	6,85E-06	AKR1C4	0,7732344	2,13E-06
GCLM	1,20467101	5,07E-07	UBQLN1	0,77165142	1,98E-06	KIF2C	-0,8865424	7,01E-06	LOXL4	-0,8868544	2,13E-06
KIF21A	1,52612401	5,07E-07	EAF1	0,8657232	1,98E-06	ANKEF1	0,74938304	7,07E-06	PSMB4	0,82970764	2,13E-06
PSMD1	1,01599132	5,21E-07	SOX9	-0,8302291	1,98E-06	PSMD2	0,64042304	7,08E-06	RAD51AP1	-1,194497	2,13E-06
RTN3	1,0299252	5,21E-07	UBXN4	0,84899562	1,98E-06	EGF	0,92929712	7,08E-06	SLC48A1	0,88460389	2,13E-06
TYMS	-1,3600998	5,35E-07	ATG4A	1,07825919	1,98E-06	PGRMC1	0,66779095	7,12E-06	PSMA6	0,74425859	2,21E-06
MAGI1	-0,8562026	5,35E-07	HSP90AB1	0,75100776	1,98E-06	OSBPL8	-0,5875604	7,12E-06	FOXM1	-1,1868917	2,22E-06
RUNX1T1	-0,9133855	5,52E-07	NPLOC4	0,97552604	1,98E-06	PSMC2	0,69333032	7,33E-06	DHRS7	0,77069755	2,36E-06
PSMB3	1,4196582	5,52E-07	MXD1	0,95064598	2,02E-06	SSTR1	0,65678306	7,33E-06	LGALS8	0,7581179	2,36E-06
ANKRD39	0,93985917	5,59E-07	LBH	-0,8315034	2,08E-06	PCYT1B	0,70035341	7,33E-06	BIRC5	-0,7467025	2,39E-06

WFS1	1,37948121	1,00E-07	HIST1H2BI	-1,537411	1,94E-06
WEE1	-1,4329492	1,01E-07	SKA1	-1,4317151	1,94E-06
KRT34	-1,4956193	1,01E-07	BRIP1	-1,9625695	1,94E-06
GSR	1,54429578	1,04E-07	KIF15	-1,2789338	1,99E-06
EPS15L1	1,6005944	1,04E-07	PAK6	-0,9998501	2,00E-06
PSMD13	1,28855579	1,14E-07	FANCD2	-1,1983831	2,05E-06
TNFRSF19	-1,2593951	1,14E-07	CCNA2	-1,2780962	2,07E-06
HSPD1	1,19181868	1,14E-07	NRIP3	1,14802523	2,07E-06
IFI44	2,0673846	1,15E-07	DHCR24	-0,9163407	2,07E-06
PSMD11	1,3039617	1,16E-07	MCM10	-0,9800962	2,07E-06
ROBO2	-1,4149568	1,17E-07	ABHD3	1,07666659	2,07E-06
LOC344887	1,60439779	1,17E-07	KIF4A	-1,4151152	2,07E-06
KIF11	-1,4808143	1,18E-07	DEPDC1	-1,3419874	2,07E-06
ITGB8	-1,5071423	1,22E-07	APOBEC3B	-0,7953	2,20E-06
SGOL1	-1,3564159	1,23E-07	HIST1H3F	-1,5420979	2,22E-06
KIF23	-1,3015438	1,31E-07	CEP55	-1,6288188	2,23E-06
LMCD1	-1,1854921	1,31E-07	BRF2	0,87628722	2,29E-06
AURKA	-1,105362	1,33E-07	MSC	0,90282271	2,29E-06
NUSAP1	-1,2755745	1,33E-07	CDC20	-0,9674186	2,33E-06
FGF10	-1,8880269	1,33E-07	KIF23	-1,2598603	2,42E-06
TYMS	-1,2382578	1,33E-07	TTC4	0,93886283	2,48E-06
ATP6V1A	1,20924183	1,33E-07	TALDO1	0,97834319	2,52E-06
CASC5	-1,6915055	1,37E-07	AURKA	-0,9818118	2,52E-06
KLHL21	0,98753826	1,38E-07	HIST1H2AB	-1,7527868	2,67E-06
NSFL1C	0,98355242	1,40E-07	NUF2	-1,5219473	2,67E-06
TTLL7	1,50880529	1,46E-07	DHCR7	-0,9965207	2,67E-06
MKI67	-1,8786518	1,47E-07	TMEM57	1,04260828	2,67E-06
NDC80	-1,6649477	1,50E-07	MXRA5	-1,532482	2,80E-06
FAM65B	-1,2232072	1,50E-07	KIAA0101	-1,3752377	2,80E-06
FKBP4	1,15336556	1,50E-07	EXO1	-1,4626285	2,85E-06
KIF18A	-1,4858996	1,50E-07	TRIM16	0,81216808	2,85E-06
TTC4	1,03829523	1,55E-07	ADRM1	0,99674674	2,97E-06
PPAP2B	-1,0779432	1,55E-07	AKR1B10	1,20581058	3,00E-06
CLPB	1,18259822	1,57E-07	NSFL1C	0,79075179	3,07E-06
MED31	1,44503044	1,69E-07	BSDC1	0,85982972	3,09E-06
STIP1	1,2186407	1,72E-07	EPGN	1,65044493	3,11E-06
CKAP2L	-1,3355587	1,79E-07	KCNA4	-1,1190482	3,25E-06
ESCO2	-1,4529965	1,79E-07	AGTRAP	0,79226397	3,27E-06
BMPER	-1,1826355	1,79E-07	HIST1H3C	-1,2789199	3,31E-06
OSGIN1	1,22295901	1,81E-07	TK1	-0,9426626	3,31E-06
PRR11	-1,5790144	1,83E-07	TRIP13	-1,0025865	3,31E-06
ATL1	1,32082014	1,85E-07	ZWINT	-0,8272993	3,37E-06
MXD1	1,11145661	1,85E-07	NQO1	0,80364317	3,37E-06
CCRL1	-1,1744016	1,91E-07	HMGB2	-0,8992334	3,66E-06
TXNIP	-1,3244328	1,91E-07	FGF10	-1,4346541	3,83E-06
PSMB7	1,05170749	1,91E-07	PSMB4	0,89143226	3,86E-06
DOLPP1	1,1336423	2,00E-07	FANCI	-1,3137629	3,89E-06
DNAJA1	0,99986471	2,05E-07	PTGR1	0,94123625	3,92E-06
LIMA1	-0,8848128	2,05E-07	DAP3	0,83189232	4,04E-06
KIT	-1,5212102	2,05E-07	UFD1L	0,95642097	4,06E-06
PSMC6	1,35733159	2,05E-07	AIFM2	0,74464827	4,06E-06
KATNB1	0,88193422	2,14E-07	PSMD14	1,08083341	4,12E-06
HSPE1	1,13316556	2,20E-07	GFRA1	-0,8113755	4,29E-06
FAM129A	1,34350373	2,20E-07	IGFBP5	-1,057338	4,36E-06
OTTHUMG0000	1,38080428	2,20E-07	CDK1	-1,7153983	4,36E-06
GSTM5	1,30970431	2,21E-07	TDP2	0,85659481	4,64E-06
DLGAP5	-1,7635557	2,26E-07	MMP3	0,7408112	4,64E-06
CCDC134	0,90073897	2,27E-07	FAM219A	1,13679263	4,64E-06
ATG4A	1,21385484	2,34E-07	SEMA3D	-1,0253953	4,64E-06
EDN1	-0,8658169	2,34E-07	SQRDL	0,92310386	4,86E-06
PSMD1	1,05107669	2,34E-07	PITHD1	0,90701997	4,89E-06
MAGI1	-0,9387428	2,34E-07	CENPI	-1,346978	5,01E-06
MEOX2	-1,5949708	2,34E-07	PSMB2	0,83231712	5,04E-06
MYLK	-1,0224177	2,34E-07	TNFSF9	1,09415731	5,13E-06
PSMC4	1,65527202	2,34E-07	PSMC2	0,94157853	5,32E-06
DESI1	1,2684932	2,34E-07	FANCA	-0,806133	5,57E-06
EMP2	-0,9670734	2,35E-07	PIR	1,22260988	5,57E-06
LMNB1	-1,390928	2,37E-07	HIST1H2BM	-1,3332641	5,58E-06
MAP1A	1,21091566	2,37E-07	ZNF367	-0,8081469	5,58E-06
KIF21A	1,56127048	2,42E-07	ITGB3	1,17268882	5,58E-06
BLOC1S2	1,03643099	2,43E-07	BLVRB	1,15236805	5,58E-06
C20orf24	0,9907077	2,43E-07	CCR1	0,79483456	5,58E-06
PSMD3	1,29364769	2,43E-07	HIST1H3J	-1,2571755	5,73E-06
PRC1	-1,4112728	2,44E-07	TENM2	-1,1690467	5,76E-06
GCLM	1,25402407	2,44E-07	MYBL2	-0,7644091	5,76E-06
NPC1	1,23653229	2,44E-07	CDCA2	-1,3430575	5,76E-06
SCG5	-0,9219831	2,48E-07	PSMC1	0,79155677	5,79E-06
UACA	-1,2437794	2,53E-07	AURKB	-0,9835917	5,79E-06
MYBL1	-1,0853371	2,60E-07	TCF19	-1,0840451	5,93E-06
IPO13	1,01802038	2,60E-07	SLC3A2	0,76178012	5,93E-06
PSMB3	1,52619852	2,67E-07	ELN	-0,8830192	5,93E-06
ATP8A1	1,19765103	2,67E-07	DTL	-1,1656558	5,93E-06
PBK	-1,4171287	2,70E-07	RAD51AP1	-1,1241359	6,00E-06
CENPF	-1,5872568	2,78E-07	IFI44	1,1262126	6,25E-06
PLA2G16	1,4267808	2,78E-07	LOC1005060	-1,0791886	6,25E-06

PSMD8	0,7855204	5,59E-07	FAXDC2	-1,0151492	2,08E-06	TYMS	-0,850366	7,33E-06	ZWINT	-0,8335596	2,39E-06
CTH	1,31403706	5,59E-07	PSMD8	0,77019533	2,08E-06	C3orf72	1,05579978	7,47E-06	PSMD6	0,65944384	2,39E-06
GSTM5	1,30000783	5,71E-07	UACA	-1,1533965	2,14E-06	HMCN1	-0,7829793	7,53E-06	NRIP3	1,11495905	2,44E-06
NDUFAB1	0,90218971	5,95E-07	VAMP1	0,73202864	2,17E-06	TALDO1	0,82778901	7,59E-06	MELK	-0,9699532	2,46E-06
NCAPG	-1,6697226	5,95E-07	MCM5	-0,7751938	2,18E-06	WNT16	0,68138514	7,62E-06	PITHD1	0,93118963	2,57E-06
UACA	-1,2092659	5,95E-07	PSMC6	1,2469455	2,20E-06	PSMD8	0,65174296	7,65E-06	EXO1	-1,5058211	2,78E-06
PSMB2	1,09745766	6,03E-07	C16orf93	0,92496172	2,23E-06	PAQR5	0,99199177	7,65E-06	TALDO1	0,92364068	2,79E-06
NXPE3	1,27825495	6,03E-07	RUNX1T1	-0,878879	2,31E-06	PSMC1	0,70242974	7,65E-06	SGK494	-0,6746236	2,79E-06
PPP2R2C	1,15595323	6,03E-07	AURKA	-1,0251744	2,32E-06	PDCD4	-0,7116524	7,72E-06	UACA	-0,8737511	2,80E-06
BABAM1	0,96004332	6,03E-07	BABAM1	0,91703534	2,35E-06	HUNK	-0,7497716	7,73E-06	DMGDH	0,68222734	2,80E-06
PIR	1,0064169	6,14E-07	HTATIP2	0,9257467	2,35E-06	NEGR1	-0,9285626	7,74E-06	PSMB7	0,71301791	2,80E-06
SH2D4A	-1,0552279	6,14E-07	HSPD1	0,75003162	2,35E-06	ADHFE1	0,66518928	7,74E-06	ABHD3	1,05229543	2,83E-06
KATNB1	0,82500882	6,26E-07	POMP	0,773422	2,38E-06	PSMC3	0,68701919	7,91E-06	GFRA1	-0,732201	2,85E-06
TRIM59	-0,9832857	6,26E-07	CDCA8	-0,8983061	2,38E-06	CKAP2	-0,7985017	7,92E-06	NCAPG2	-1,1954949	2,91E-06
SNX21	0,89255019	6,32E-07	POLR2C	0,80610277	2,43E-06	AKR1C2	0,71714152	7,92E-06	FAM129A	1,05335653	2,91E-06
RTN3P1	1,00577982	6,41E-07	MAGI1	-0,7951579	2,43E-06	GSR	0,91115777	8,01E-06	KIF20B	-1,3616943	2,95E-06
MPHOSPH1(	0,91349705	6,41E-07	SLC45A4	0,80579269	2,43E-06	FOLR3	0,71797914	8,01E-06	BRF2	0,81046597	2,96E-06
POPDC3	1,1581519	6,41E-07	PLEKHB2	0,87223911	2,43E-06	FOXM1	-0,8664973	8,05E-06	CKAP2	-0,9026209	2,97E-06
KRT34	-1,4073982	6,61E-07	RTN3P1	0,949484	2,49E-06	KIF23	-0,7419653	8,08E-06	SKA3	-1,3522408	2,97E-06
IPO13	0,93445913	6,61E-07	DMGDH	1,22169662	2,55E-06	TSPAN2	-1,0560474	8,10E-06	FGF10	-1,4475176	3,04E-06
CLN8	0,93632087	6,67E-07	POP1	1,1894398	2,58E-06	CDCP1	0,83439496	8,10E-06	CDCA3	-0,817572	3,04E-06
DEDD2	0,86327616	6,68E-07	AKR1C2	0,8308734	2,59E-06	RAB27B	1,1837657	8,10E-06	ARHGAP11A	-1,1876842	3,09E-06
UBE2O	0,88129333	6,68E-07	NAA25	1,46472948	2,62E-06	CCBE1	0,71278392	8,10E-06	DTL	-1,2154845	3,09E-06
OXTR	-0,8135353	6,68E-07	EGF	1,08667192	2,62E-06	HSD17B4	0,71970535	8,23E-06	CDC25C	-0,8205129	3,16E-06
ROBO2	-1,4625334	6,68E-07	DNTTIP2	0,92160331	2,62E-06	CENPF	-0,9426216	8,23E-06	USP5	0,8175276	3,17E-06
KIF11	-1,6159022	6,68E-07	ZNF608	-0,9981369	2,62E-06	SFRP4	-0,665523	8,23E-06	TXNL1	0,66691469	3,23E-06
ADRM1	0,97758943	6,68E-07	TYMS	-0,9938029	2,69E-06	SMC2	-0,7077563	8,23E-06	PSMB2	0,83477006	3,23E-06
ABCB6	0,80096379	6,69E-07	PQLC2	0,81521364	2,69E-06	TTC4	0,61274953	8,23E-06	SPC25	-1,131268	3,24E-06
ADAMTS12	0,88961038	6,69E-07	INO80B	0,73057835	2,69E-06	HFE	0,83674994	8,23E-06	NEGR1	-0,92811	3,24E-06
FRMD6	-0,8537814	6,69E-07	UCHL1	0,84184884	2,70E-06	KCTD4	0,84075784	8,23E-06	TMEM97	-0,8495963	3,31E-06
MX1	1,20820386	6,85E-07	C20orf24	0,85547981	2,70E-06	UACA	-0,6888109	8,23E-06	AURKB	-1,06536	3,35E-06
CDH11	-0,8837015	6,94E-07	PSMA1	0,91292892	2,70E-06	PSMD1	0,78158532	8,23E-06	NQO2	0,6627986	3,37E-06
HIST1H2BF	-1,2275965	6,98E-07	TTC4	1,01429573	2,70E-06	POPDC3	0,90781847	8,23E-06	FANCA	-0,7803198	3,43E-06
IFI44	1,92564791	6,98E-07	MYLK	-0,7839424	2,71E-06	DIAPH3	-1,0941355	8,23E-06	HELLS	-1,6503748	3,44E-06
HIST1H2AB	-1,543179	6,98E-07	HAS2	-1,1282876	2,71E-06	PRDX2	0,67987361	8,23E-06	SMC2	-0,8277895	3,46E-06
IL27RA	0,99957042	6,98E-07	ZNF367	-0,8827038	2,76E-06	PSMB1	0,65813515	8,23E-06	AGTRAP	0,69018989	3,47E-06
PLAA	1,09895134	7,02E-07	SLC24A6	0,77770751	2,77E-06	TTK	-0,7272801	8,23E-06	PSMB6	0,66431885	3,92E-06
HSP90AA1	0,91915852	7,02E-07	GADD45A	0,77573842	2,80E-06	NQO2	0,67645632	8,23E-06	AKR1C3	0,78912753	3,99E-06
PSMA5	1,08513714	7,02E-07	DSP	-0,8746494	2,80E-06	KIF4A	-0,9775771	8,23E-06	OSGIN1	0,68242834	4,02E-06
PSMA4	1,0190874	7,02E-07	KCTD5	0,91348187	2,86E-06	SPRY2	0,71349551	8,31E-06	PSMD1	0,76157922	4,08E-06
TNFSF9	1,42699489	7,02E-07	KIAA0368	0,93447746	2,86E-06	CDCA8	-0,6574143	8,37E-06	TNFSF9	1,13225032	4,09E-06
PSMC5	1,03500781	7,02E-07	AKR1B1	0,68987903	2,86E-06	TRIB2	-0,6623818	8,62E-06	KCNA4	-1,0463265	4,09E-06
EDN1	-0,8978846	7,17E-07	EPB41	0,97669679	2,92E-06	MRGPRF	0,61011131	8,63E-06	PSMC1	0,76963356	4,17E-06
ACOT13	1,0998054	7,29E-07	IKBKE	0,67676628	2,92E-06	HIST1H1B	-1,3824085	8,63E-06	VCAN	-0,7214562	4,29E-06
CITED2	-0,8119705	7,30E-07	AHSA1	0,9212948	2,93E-06	CCNA2	-0,744736	8,66E-06	HIST2H2AA	-0,8251029	4,29E-06
RRP12	0,77396645	7,31E-07	TRIM59	-0,8725913	2,93E-06	CKAP2L	-0,8164677	8,99E-06	PSMC6	0,93459326	4,29E-06
HTR2B	-1,1085646	7,41E-07	WEE1	-1,2764855	2,95E-06	CYP7B1	-0,947573	9,03E-06	PLCE1	-0,7212685	4,47E-06
ZNF608	-1,0972889	7,41E-07	KATNB1	0,72181497	3,01E-06	AKAP6	-0,7749991	9,42E-06	UCHL1	0,76589216	4,51E-06
AFF1	0,91603932	7,43E-07	AKR1C3	0,85245931	3,07E-06	POMP	0,58854851	9,42E-06	PSMA1	0,76943743	4,57E-06
ARHGAP11A	-1,3166422	7,43E-07	PSMC1	0,90367824	3,07E-06	HJURP	-0,7414701	9,60E-06	LOC344887	1,35273826	4,67E-06
OSR2	-1,1810105	7,43E-07	TRIB2	-0,820803	3,08E-06	KIAA0319	0,66186473	9,64E-06	ACOT13	0,75286991	4,72E-06
CHORDC1	1,34128535	7,43E-07	SYT1	-0,8267327	3,08E-06	AIM1	0,7887295	9,64E-06	SCUBE3	-0,8611234	4,75E-06
PSMA6	0,90828417	7,43E-07	IL27RA	0,95902683	3,14E-06	OXTR	-0,5858475	9,65E-06	CCNE2	-1,2356287	4,80E-06
EPHX4	1,47324733	7,56E-07	AFF1	0,81919255	3,20E-06	RTN3P1	0,78177677	9,65E-06	HIST1H4L	-1,2228441	4,91E-06
EPB41	1,05383657	7,57E-07	PHTF1	1,22757164	3,20E-06	CLU	0,60376804	9,99E-06	MAPRE3	0,63818336	4,94E-06
TMEM259	0,84741934	7,65E-07	KRTAP1-1	-0,8068019	3,24E-06	SLC40A1	-0,6989216	1,01E-05	AKR1B1	0,58754603	5,03E-06
AKR1C1	1,00060616	7,75E-07	KRT34	-0,9770502	3,25E-06	PSMD4	0,62117809	1,04E-05	PSMB1	0,65572685	5,12E-06
KLHL21	0,91068018	7,87E-07	TMEM37	0,83693354	3,25E-06	KCNA4	-0,7555882	1,04E-05	WDR76	-0,8062863	5,15E-06
HIST1H3J	-1,1469055	7,89E-07	ARRDC4	1,2682886	3,25E-06	NCAPG	-0,8350745	1,05E-05	SNX10	0,58551002	5,23E-06
SYT1	-0,8983219	8,14E-07	PFN2	0,73046553	3,27E-06	TTLL7	0,92235628	1,05E-05	HIST1H1B	-1,481982	5,38E-06
DOLPP1	1,02817173	8,14E-07	EDN1	-0,7172223	3,36E-06	FPR1	0,81179428	1,12E-05	LMCD1	-0,6759737	5,38E-06
CCDC134	0,90215404	8,14E-07	HSPA9	0,79400942	3,36E-06	ESM1	1,08301897	1,13E-05	PSMD12	0,81810738	5,40E-06
PSMC6	1,24845886	8,16E-07	TMEM140	0,93525034	3,42E-06	PSMB5	0,7063246	1,15E-05	TRIP13	-1,0190878	5,49E-06
PSMD12	1,16834733	8,18E-07	DYNLL2	0,90987481	3,42E-06	C5orf30	0,6518547	1,16E-05	MYC	0,65079134	5,53E-06
IMPDH2	-0,8648197	8,23E-07	MEOX2	-1,1201442	3,42E-06	RTN3	0,69980697	1,21E-05	CTSL1	0,65317635	5,61E-06
INO80B	0,81156706	8,25E-07	ROBO2	-1,0813306	3,52E-06	GK	1,09913226	1,23E-05	TK1	-0,9897061	5,63E-06
OTTHUMG0	1,30849953	8,26E-07	ADPGK	0,95862158	3,52E-06	PAK6	-0,7105963	1,23E-05	LINC00707	-1,198518	5,74E-06
NPLOC4	0,98510581	8,29E-07	DNAJA1	0,77738676	3,52E-06	C20orf24	0,64897646	1,23E-05	ATP6V0A1	0,88515492	5,88E-06
KIAA0930	0,74514439	8,29E-07	TNS3	-0,8729952	3,52E-06	PDE5A	-0,8573829	1,24E-05	BLM	-0,9877051	5,98E-06
PQLC2	0,87366601	8,31E-07	SGOL1	-1,0668551	3,55E-06	GPR137C	-0,876229	1,26E-05	HIST1H2BF	-1,2679782	6,01E-06
CDH2	-0,935466	8,31E-07	KIT	-1,4071976	3,55E-06	BTG1	-0,622679	1,30E-05	PSMA7	0,59679406	6,04E-06
PLBD2	0,81173069	8,31E-07	OXTR	-0,7257384	3,56E-06	MUC13	0,73312984	1,31E-05	PLIN2	0,59882937	6,08E-06
HSP90AB1	0,89582194	8,31E-07	TNFRSF19	-1,0466987	3,56E-06	ZNF704	-0,7434539	1,32E-05	KRTAP1-5	-0,6706445	6,14E-06
TNS3	-0,96018	8,31E-07	TNFSF13B	1,07250363	3,56E-06	LPXN	0,62434451	1,32E-05	GDF15	0,7993772	6,15E-06
POLR3E	0,87431552	8,31E-07	DEDD2	0,69542394	3,56E-06	HIST1H3A	-1,0838583	1,33E-05	CENPI	-1,3694689	6,15E-06
SELK	0,85110532	8,31E-07	FBXO5	-0,9830613	3,56E-06	MECOM	0,7929666	1,33E-05	PSMC5	0,71057066	6,15E-06
PDE5A	-1,1531849	8,43E-07	HIST1H1A	1,05602012	3,56E-06	PSMA6	0,66965812	1,33E-05	RNF150	-0,676605	6,15E-06
ANGPTL1	1,4128907	8,43E-07	HMGB1P4	-0,6843635	3,56E-06	CPEB1	0,69257542	1,34E-05	PLAA	0,80858406	6,27E-06
OSER1	0,84630589	8,43E-07	ACOX2	0,75089634	3,56E-06	ATP8A1	0,81355309	1,34E-05	ZNF367	-0,8929221	6,27E-06
DSP	-0,946583	8,43E-07	OSER1	0,87549593	3,56E-06	HMGB1P5	-0,6560384	1,35E-05	C20orf24	0,68987027	6,29E-06
BCL2	-1,076678	8,49E-07	IPO13	0,8962395	3,56E-06	MYPN	0,91796858	1,36E-05	KIF14	-1,2283086	6,29E-06
AKR1B15	0,92678927	8,49E-07	C12orf10	0,78826833	3,56E-06	TMEM255A	0,59720539	1,36E-05	RACGAP1	-0,9151233	6,49E-06
EAF1	0,9225935	8,50E-07	APOL2	0,68568199	3,56E-06	ITGB8	-0,8285458	1,38E-05	HIST1H2AM	-1,0694765	6,56E-06
NEK7	-0,9883568	8,50E-07	OTTHUMG0	1,83893281	3,62E-06	MAN2B1	0,61279995	1,38E-05	HIST1H3G	-1,1516408	6,67E-06
FHOD1	0,80226939	8,64E-07	ANKRD39	0,8846522	3,64E-06	CA5B	-0,6130907	1,38E-05	TDP2	0,70612555	6,69E-06

NXPE3	1,2973457	2,78E-07	CTSL1	0,74808458	6,25E-06
AKR1B1	1,03744433	2,82E-07	HIST1H2BF	-1,1812542	6,25E-06
RUNX1T1	-0,9772162	2,83E-07	NQO2	0,68188425	6,25E-06
PSMB2	1,11802906	2,83E-07	MYBL1	-0,71573	6,25E-06
CDC20	-1,048389	2,84E-07	HSPA1A	0,85093209	6,25E-06
RBMS3	-0,9796578	3,01E-07	CCDC15	-0,8563914	6,44E-06
FAM111B	-2,0087206	3,01E-07	HSPA1B	1,10820191	6,71E-06
DNAJC2	1,08084255	3,02E-07	CENPF	-1,7628771	7,28E-06
TNFSF9	1,4994692	3,02E-07	BLM	-1,0017319	7,29E-06
EGF	1,24925643	3,05E-07	HELLS	-1,7727708	7,44E-06
NCAPG	-1,478711	3,05E-07	DHRS7	0,92055121	7,75E-06
BSDC1	1,32559403	3,05E-07	CCNE2	-1,2097941	7,86E-06
HSP90AB1	0,92102939	3,05E-07	CCNB2	-1,2994135	8,07E-06
PSMC5	1,11406007	3,05E-07	KRT34	-0,7278529	8,83E-06
HIST1H3F	-1,3893187	3,20E-07	PLIN2	0,67810906	8,96E-06
SLC46A3	1,25748714	3,21E-07	ARHGAP11A	-1,2444898	9,10E-06
ACOT13	1,2458482	3,21E-07	UCHL1	0,7798434	9,19E-06
TNS3	-1,0266922	3,21E-07	SCUBE3	-1,0886587	9,19E-06
FAM102A	0,97336949	3,24E-07	KIF11	-1,6890526	9,30E-06
PSMD12	1,19341615	3,24E-07	NEIL3	-1,2256286	9,96E-06
ACO2	1,12710441	3,24E-07	PSMC6	1,04107309	1,01E-05
PLAA	1,13173797	3,24E-07	C14orf1	-0,6988358	1,03E-05
LMO7	-1,0982608	3,24E-07	PSMA3	1,12944385	1,06E-05
PSMB4	1,15691463	3,24E-07	UACA	-0,9813168	1,06E-05
SOX9	-0,9817671	3,24E-07	CDC25C	-0,754289	1,06E-05
MELK	-1,1612642	3,24E-07	USP5	0,77705971	1,08E-05
E2F6	0,98029709	3,24E-07	SPC25	-1,115512	1,08E-05
PSMA6	0,97544059	3,24E-07	PSMD6	0,76064496	1,08E-05
DNTTIP2	1,04379044	3,38E-07	ATAD2	-1,1716227	1,10E-05
USP14	1,02007437	3,41E-07	NCAPG2	-1,2544834	1,10E-05
SLC25A38	1,05217345	3,42E-07	MELK	-0,9482879	1,10E-05
RTN3	1,02765127	3,43E-07	PLAA	0,80339317	1,11E-05
SPP1	1,20266763	3,43E-07	ATP6V1A	0,7361835	1,13E-05
PSMA4	1,07449819	3,44E-07	HIST1H2BH	-0,7763619	1,15E-05
DEPDC1	-1,4710962	3,46E-07	SNX10	0,68053346	1,15E-05
PPP2R2C	1,25045023	3,47E-07	RAD54L	-0,6553789	1,15E-05
ADHFE1	0,90912482	3,50E-07	PSMB6	0,75144775	1,15E-05
TMEM37	0,9190692	3,51E-07	COL12A1	-1,0127253	1,15E-05
SLC2A1	1,02090527	3,62E-07	MMP10	0,97511233	1,15E-05
ABCB6	0,87379707	3,62E-07	LMCD1	-0,6946687	1,17E-05
PGD	0,93962941	3,62E-07	MAPRE3	0,60863616	1,17E-05
POP1	1,13047886	3,62E-07	TICRR	-1,1414198	1,19E-05
TMEM140	1,0431607	3,62E-07	VEZT	0,7535116	1,20E-05
FRMD6	-0,8241788	3,62E-07	SQSTM1	0,67904686	1,21E-05
INTS10	1,22435247	3,62E-07	SLC48A1	0,86388915	1,28E-05
TM2D2	1,24206871	3,62E-07	STC1	-0,6647182	1,28E-05
ATAD2	-1,2380111	3,67E-07	KCNJ6	-0,9758447	1,31E-05
AIFM2	0,93965834	3,73E-07	HIST1H3G	-1,1025283	1,31E-05
PIR	1,0918793	3,73E-07	PSMB7	0,78137853	1,31E-05
MXRA5	-1,0399039	3,75E-07	PSMD1	0,73738046	1,33E-05
ADAMTS12	0,93054397	3,79E-07	LGALS8	0,7416778	1,34E-05
AMOT	-0,8571182	3,79E-07	ABCB6	0,63764132	1,34E-05
DEK	-0,9061238	3,79E-07	OSER1	0,73290324	1,34E-05
MAMLD1	-0,9276585	3,79E-07	NEGR1	-0,9296941	1,37E-05
DYNLL2	1,14917473	3,79E-07	ATP6V0B	0,88927961	1,39E-05
RRP12	0,83382677	3,90E-07	VCAN	-0,962465	1,42E-05
PSMC1	0,94619055	3,94E-07	ACOT13	0,82790711	1,42E-05
UBR3	1,19214286	3,95E-07	CDC20P1	-1,0339724	1,44E-05
RTN3P1	1,04271399	3,97E-07	PSMD4	0,62492426	1,46E-05
TXNL1	0,88193162	4,06E-07	C20orf24	0,73899116	1,47E-05
SH2D4A	-0,9893636	4,18E-07	CDC45	-0,7195203	1,47E-05
TMEM161A	1,16096206	4,18E-07	HIST1H2BO	-1,0239649	1,49E-05
HSP90AA1	0,93328573	4,30E-07	GTSE1	-0,813912	1,54E-05
CDCA2	-1,2408316	4,30E-07	GDF15	0,76211995	1,54E-05
ZNF608	-1,0517484	4,41E-07	RACGAP1	-0,8378994	1,57E-05
FAXDC2	-1,0128974	4,43E-07	POLQ	-1,2258487	1,60E-05
MARCKS	-0,9546028	4,46E-07	FBXO5	-0,9042224	1,60E-05
AJUBA	-1,0456152	4,46E-07	LOXL4	-0,887968	1,61E-05
SORD	1,23072368	4,46E-07	CBR1	0,67398128	1,62E-05
OCLN	1,2571698	4,46E-07	PSMA5	0,97908291	1,63E-05
VAMP1	0,89140467	4,46E-07	PSMA1	0,86954968	1,64E-05
FHOD1	0,82886494	4,50E-07	PLCE1	-0,8188652	1,64E-05
CNKSR3	1,01597039	4,50E-07	MMP1	0,61019389	1,64E-05
PSMC2	0,85910869	4,55E-07	MEOX2	-0,8732199	1,64E-05
C18orf54	-1,5649015	4,58E-07	PSMD12	0,79592634	1,68E-05
AKR1C1	1,0173567	4,61E-07	TRIM16L	0,74874334	1,76E-05
SMC2	-1,0541149	4,65E-07	PSMC5	0,68354372	1,78E-05
KIF15	-1,1416188	4,65E-07	NIT2	0,9113821	1,80E-05
CDH11	-0,912676	4,65E-07	PTGES	0,74361095	1,83E-05
CCNB2	-1,2706804	4,65E-07	FKBP4	0,67807708	1,86E-05
BRIP1	-1,5457546	4,65E-07	KRTAP1-5	-0,6682087	1,87E-05
STMN2	-0,8073844	4,67E-07	CPNE8	0,89610099	1,87E-05
HSPA9	0,88350953	4,72E-07	SOX9	-0,6582975	1,88E-05
HIST1H2BF	-1,136133	4,74E-07	HUNK	-0,7330954	1,92E-05
SEMA3D	-1,0218096	4,74E-07	CDCA3	-0,7279901	1,94E-05

PLEKHB2	0,94831089	8,75E-07	GABARAPL	0,91100667	3,74E-06	PHKB	0,77092696	1,38E-05	SQSTM1	0,68177572	6,69E-06
DNAJA1	0,98852576	8,75E-07	NECAB3	0,64822665	3,74E-06	MAP1A	0,73916902	1,39E-05	FABP3	-0,8568421	6,82E-06
C20orf24	0,88192408	8,75E-07	SLC46A3	1,10395914	3,75E-06	MELK	-0,6932802	1,40E-05	ATP6V1A	0,7330148	6,82E-06
MXRA5	-1,008492	8,75E-07	E2F6	0,96322553	3,75E-06	HIST1H3D	-1,0617828	1,44E-05	LGR5	-0,9792019	6,82E-06
CADM1	-0,8035033	8,75E-07	PPAP2B	-0,8069175	3,76E-06	NKX3-1	0,63430354	1,46E-05	CSRP2	-0,701346	6,84E-06
KIAA0754	-0,9130552	8,75E-07	TANGO2	0,69281192	3,83E-06	RACGAP1	-0,7582157	1,51E-05	PTGES	0,63965818	6,89E-06
NTM	-0,7508657	8,76E-07	PRDX2	0,64144659	3,92E-06	NCAPG2	-0,8963445	1,51E-05	HIST1H2AG	-1,2845688	7,12E-06
FABP3	-1,11394	8,76E-07	LMNB1	-1,2924781	3,97E-06	GTSE1	-0,6020265	1,56E-05	HIST1H2BH	-0,7502639	7,19E-06
KIAA0368	0,97755338	8,96E-07	MED31	1,20378664	4,27E-06	HIST1H4L	-1,1547517	1,57E-05	ATAD5	-0,8962684	7,19E-06
TM2D2	1,14352359	9,22E-07	FEZ1	0,89295638	4,29E-06	OTTHUMG0	-1,1494524	1,58E-05	MIR222	1,31293027	7,19E-06
CCRL1	-1,0869605	9,56E-07	SLC10A7	1,31814065	4,34E-06	VAMP1	0,68779034	1,59E-05	EPGN	1,5362824	7,27E-06
TRIB2	-0,9141899	9,56E-07	NPC1	0,93276584	4,40E-06	ASPN	-0,7891217	1,61E-05	HUNK	-0,6987854	7,46E-06
ZNF367	-0,9329026	9,56E-07	SQSTM1	0,72130103	4,47E-06	SAMD9L	0,70603308	1,62E-05	NEIL3	-1,3601428	7,47E-06
UBR3	1,19552108	9,56E-07	CENPT	0,73067684	4,47E-06	NCAPD2	-0,6258075	1,63E-05	EPS15L1	0,80236095	7,50E-06
HSPE1	1,08513484	9,66E-07	RTCA	0,94504504	4,48E-06	KIF14	-1,0333482	1,67E-05	VEZT	0,73809829	7,69E-06
HMGN2P18	-0,8135195	9,71E-07	POLR3E	0,73451568	4,49E-06	MT2A	0,61443227	1,68E-05	PSMB3	0,9066479	7,95E-06
MED31	1,21964594	9,74E-07	PSMA2	1,07943137	4,54E-06	HTR2A	0,85857504	1,68E-05	FAM65B	-0,7083259	7,96E-06
SPP1	1,05660907	9,75E-07	ORC3	1,02069135	4,55E-06	LRRCC1	-0,6890125	1,68E-05	NPC1	0,84636108	8,02E-06
LIMA1	-0,8851442	9,77E-07	POC5	1,11105054	4,68E-06	LOXL1	0,58989639	1,69E-05	EZH2	-0,922518	8,02E-06
SLC4A4	-1,1737426	9,82E-07	TPX2	-1,2051312	4,71E-06	FAM219A	0,93677541	1,70E-05	ELN	-0,7362863	8,06E-06
POP1	1,10147634	9,92E-07	CNKSR3	1,1110328	4,80E-06	HIST1H2AB	-1,6419072	1,70E-05	CDC45	-0,7449374	8,06E-06
RGCC	-0,8267771	1,02E-06	CDH11	-0,7291785	4,81E-06	CEP55	-0,9810989	1,72E-05	SOX9	-0,6309211	8,15E-06
WBP2	0,85988055	1,05E-06	BCOR	-0,7920569	4,82E-06	FANCI	-0,8152956	1,77E-05	FOLR3	0,72326095	8,15E-06
SPTAN1	0,74987995	1,05E-06	KIF21A	1,37059391	4,82E-06	MT1CP	0,76790902	1,81E-05	DESI1	0,73889728	8,29E-06
OTTHUMG0	1,01119089	1,05E-06	L3MBTL2	0,82397972	4,83E-06	LMCD1	-0,6191761	1,82E-05	MCM8	-0,959172	8,37E-06
SLC45A4	0,77084523	1,05E-06	LDB2	-0,7321208	4,84E-06	HMGCS1	-0,7429278	1,90E-05	LHX9	-0,6265404	8,48E-06
EGF	1,10661189	1,05E-06	BMPER	-0,8279712	4,93E-06	SLC46A3	0,7845607	1,90E-05	NEK2	-0,8644337	8,48E-06
PCYT1A	0,94206369	1,05E-06	DEPDC1	-1,3043092	4,94E-06	HTR2B	-1,0577353	1,90E-05	COL8A1	-0,6554825	9,01E-06
HIST1H2AE	-1,5148476	1,06E-06	STARD7	0,73247847	5,06E-06	HIST1H4B	-1,0040541	1,91E-05	STOX2	0,79302755	9,23E-06
ARRDC4	1,14836014	1,07E-06	MSRB1	0,70859121	5,06E-06	DKK2	-0,6282182	1,93E-05	CTH	0,70979857	9,28E-06
SLC24A6	0,83972767	1,07E-06	SCG5	-0,7095979	5,08E-06	KIF15	-0,6957696	1,96E-05	SCD	-0,6929478	9,55E-06
GTSE1	-0,7724244	1,08E-06	EMP2	-0,819248	5,09E-06	CLPB	0,62674084	2,01E-05	PSMA4	0,74247063	9,58E-06
ARNT2	-0,8829124	1,08E-06	ABCB6	0,71886451	5,09E-06	DESI1	0,80750429	2,05E-05	TTLL7	0,82704569	9,62E-06
CRY2	0,74392719	1,09E-06	PLK1	-1,1303387	5,09E-06	ATP6V1A	0,67036937	2,05E-05	ZFAND2A	0,76202541	9,87E-06
ATAD2	-1,2914396	1,09E-06	PSMA7	0,70350457	5,22E-06	GPR124	-0,6158359	2,05E-05	UHRF1	-0,7006253	9,88E-06
MARCKS	-0,985258	1,10E-06	RRP12	0,67019723	5,22E-06	MT1JP	0,61725379	2,07E-05	TRIB1	0,85275063	9,92E-06
POLR2C	0,81258541	1,10E-06	SIN3B	0,69277019	5,34E-06	HIST1H2AL	-0,6505395	2,15E-05	MSMO1	-0,6491854	1,02E-05
KIF4A	-1,2060245	1,10E-06	COPS2	0,72925874	5,40E-06	AKR1B15	0,61392762	2,18E-05	DSP	-0,6920863	1,03E-05
MAMLD1	-0,8342596	1,10E-06	BNIP3	0,6904004	5,41E-06	HINT1	0,63063629	2,24E-05	NEK7	-0,6373958	1,03E-05
PSMC1	0,87621537	1,10E-06	PGRMC1	0,64901122	5,46E-06	DPP3	0,58793518	2,24E-05	TMEM38B	0,67669988	1,05E-05
CACYBP	0,88413027	1,10E-06	CCNF	-0,7049732	5,53E-06	PSMA4	0,70378264	2,26E-05	FBXO32	0,62007753	1,05E-05
NCLN	0,90742361	1,10E-06	SNRPD3	0,71658933	5,55E-06	PSAT1	-0,7974247	2,28E-05	HIST1H1E	-0,6204905	1,05E-05
TXNL1	0,84291379	1,11E-06	UBE2M	0,69749232	5,60E-06	ITGA2	0,76525095	2,29E-05	IFNGR1	0,63255424	1,09E-05
UCHL1	0,91803108	1,14E-06	KIF18A	-1,3122692	5,60E-06	MMP12	1,20297011	2,31E-05	FBXO5	-0,9166143	1,10E-05
COL8A1	-0,8194247	1,16E-06	TMEM259	0,69387546	5,62E-06	CDCA2	-0,7512027	2,32E-05	KIF18A	-0,8668647	1,11E-05
LOC344887	1,5971453	1,16E-06	MARK1	0,82017821	5,62E-06	PTGES	0,71583	2,34E-05	LOC1005060	-1,052046	1,11E-05
HECW1	0,89713237	1,16E-06	OTTHUMG0	-0,9336627	5,62E-06	FAM72B	-0,6972272	2,34E-05	DOCK10	0,63335162	1,13E-05
PLA2G16	1,22805216	1,16E-06	BUB1	-1,2842219	5,62E-06	OR1J4	0,812335	2,38E-05	SAMD9L	0,6362232	1,14E-05
HIST1H2AH	-0,9208087	1,16E-06	BSCL2	0,61348455	5,62E-06	NDUFAB1	0,58959749	2,40E-05	FAM72B	-0,7837164	1,14E-05
APOBEC3B	-0,7448142	1,16E-06	LRIG1	0,69689519	5,62E-06	MT1B	0,6436175	2,40E-05	KIFC1	-0,6728618	1,14E-05
ATG4A	1,08474323	1,17E-06	LOC1005060	-1,1133682	5,62E-06	EPS15L1	0,76531138	2,46E-05	USP14	0,6550801	1,15E-05
FAXDC2	-1,0155221	1,18E-06	MTX1	0,59412066	5,62E-06	PARM1	0,80108681	2,50E-05	KRT19	-0,5999505	1,16E-05
IDE	0,92916989	1,18E-06	WDR74	0,78223708	5,62E-06	CD22	0,63362624	2,52E-05	MCM7	-0,790502	1,19E-05
CLSPN	-1,336624	1,18E-06	DCTN4	0,71225378	5,63E-06	HIST1H4D	-1,263836	2,58E-05	PCNA	-0,62161	1,25E-05
AJUBA	-1,0398141	1,18E-06	IGHMBP2	0,59591502	5,66E-06	FAT4	-0,6627901	2,62E-05	DNAJC6	0,77113842	1,25E-05
PTGES	0,81170343	1,18E-06	SLMAP	0,8297211	5,71E-06	OXCT1	-0,7388844	2,62E-05	ATP6V0B	0,74138691	1,26E-05
SLC2A1	0,94850515	1,19E-06	PSMB5	0,90016074	5,71E-06	CLCC1	0,77343792	2,62E-05	LARP4	0,62884423	1,26E-05
PSMB5	0,91161535	1,19E-06	GAS8	0,75711437	5,75E-06	ANXA7	0,61205916	2,64E-05	PLK4	-1,1874992	1,26E-05
TM4SF1	-0,9010324	1,20E-06	PDGFRA	-0,7183751	5,75E-06	AQP9	-1,1399008	2,65E-05	BRCA2	-1,0381337	1,27E-05
ULBP2	0,83766941	1,20E-06	ULBP2	0,73823137	5,81E-06	BSDC1	0,6491279	2,68E-05	CENPE	-0,8642946	1,27E-05
PRDX2	0,69147765	1,20E-06	FAM102A	0,70975077	5,81E-06	NCLN	0,71369297	2,70E-05	KIF24	-0,6548647	1,29E-05
DNAJC2	1,13096027	1,20E-06	VIPAS39	0,92653493	5,89E-06	PSMA5	0,67511372	2,71E-05	MT1X	0,90454329	1,30E-05
SMO	0,757359	1,21E-06	ELFN1	-0,6951447	5,98E-06	STEAP1	0,62982292	2,83E-05	MEOX2	-0,8802274	1,31E-05
CEP55	-1,4075123	1,23E-06	PSMC3	0,76832749	6,04E-06	AFF2	-0,654657	2,85E-05	HIST1H3D	-1,1192963	1,31E-05
GABARAPL	0,93576667	1,23E-06	HMGN3	-0,6977043	6,23E-06	CDCA3	-0,6382103	2,88E-05	ANXA7	0,62526138	1,31E-05
UBQLN1	0,74392805	1,23E-06	RDH11	0,90276108	6,23E-06	HIST2H2AB	-0,9605844	2,96E-05	E2F6	0,62160398	1,37E-05
RDH11	0,91713711	1,24E-06	SLC2A1	0,78260429	6,24E-06	ADAMTS5	0,63318154	2,97E-05	SYT1	-0,5984172	1,37E-05
SWAP70	-0,7472963	1,24E-06	ATP6V0B	0,74279389	6,24E-06	AGTRAP	0,58945348	3,07E-05	OSER1	0,63768641	1,43E-05
UBR4	0,85511672	1,28E-06	FAM65B	-0,8811463	6,26E-06	SHCBP1	-0,8337265	3,09E-05	MNS1	-0,8226965	1,46E-05
SIN3B	0,77889819	1,29E-06	TACC2	0,71320229	6,28E-06	HMGA1P1	0,59060983	3,18E-05	CCNF	-0,68453	1,50E-05
KIT	-1,6698355	1,29E-06	PLA2G16	1,05140776	6,32E-06	IL27RA	0,66002549	3,35E-05	DNAJC2	0,58677089	1,50E-05
NEK6	-0,7697897	1,29E-06	YWHAG	0,70921294	6,47E-06	MT1A	0,60006403	3,37E-05	PSMC3IP	-0,8234384	1,50E-05
NUF2	-1,3739454	1,29E-06	MARCKS	-0,7996581	6,66E-06	FANCD2	-0,703872	3,37E-05	MAD2L1	-0,8167401	1,56E-05
PBK	-1,5437738	1,29E-06	PSMB1	0,6533967	6,66E-06	AKR1C7P	1,14798124	3,41E-05	HIST1H2AE	-1,1290239	1,56E-05
CDK1	-1,6170501	1,29E-06	STMN1	-0,6837799	6,70E-06	ZWILCH	-0,6784649	3,42E-05	KIAA1524	-0,9935846	1,57E-05
MAN2B1	0,76837297	1,29E-06	FKBP4	0,76841059	6,80E-06	FBN2	-0,6384845	3,42E-05	NXPE3	0,80600117	1,59E-05
NCAPG2	-1,2195095	1,29E-06	AMOT	-0,6393712	6,80E-06	KIF20B	-0,8993478	3,42E-05	PSMB5	0,67963369	1,59E-05
HSPA9	0,85321389	1,29E-06	CITED2	-0,618916	6,88E-06	HIST1H2AM	-1,1031231	3,44E-05	HIST2H2AB	-0,8450062	1,63E-05
HIST1H3B	-1,2914476	1,29E-06	LARP4	0,97471398	6,90E-06	MT1X	0,73516684	3,44E-05	BRCA1	-0,8586263	1,64E-05
HMGN2P28	-0,817389	1,29E-06	GPI	0,66669183	6,91E-06	FAM72D	-0,9535536	3,55E-05	UBE2C	-0,8094548	1,64E-05
TACC2	0,76372767	1,29E-06	FAM217B	0,83455632	6,94E-06	LGALS8	0,61227116	3,55E-05	COL3A1	-0,6388587	1,65E-05
SMC2	-1,0847794	1,29E-06	STC1	-0,8751575	6,95E-06	LOC1005060	-0,9006323	3,57E-05	HIST1H3A	-1,0772385	1,68E-05
TMPO	-1,1439837	1,29E-06	ANXA7	0,75875619	7,16E-06	HTATIP2	0,64297182	3,71E-05	OTTHUMG0	0,89243226	1,70E-05
HMMR	-1,3922187	1,29E-06	KCNJ2	-1,3254717	7,17E-06	LCE2B	0,86266209	3,71E-05	GCLC	0,60301096	1,72E-05
CTGF	-0,7663347	1,30E-06	KCNQ5	-1,0475128	7,23E-06	IDE	0,58745042	3,74E-05	PLAUR	0,62635887	1,77E-05

CLN8	1,03041666	4,74E-07	PSMA4	0,82671435	1,94E-05
PSMA1	0,95688032	4,82E-07	ASF1B	-0,8114577	1,95E-05
PDGFRA	-0,8579314	4,82E-07	SGK494	-0,6900234	2,02E-05
E2F8	-1,1494522	4,88E-07	AKR1B1	0,6333023	2,04E-05
HFE	1,05815353	4,88E-07	MCM7	-0,8514226	2,04E-05
SHCBP1	-1,3301176	5,00E-07	PLK4	-1,2333331	2,04E-05
UBB	0,79931803	5,00E-07	HTATIP2	0,72708801	2,04E-05
PLSCR4	-0,9417999	5,19E-07	MT1CP	0,5960382	2,04E-05
SIN3B	0,81231025	5,21E-07	TGM2	-0,7118471	2,04E-05
PSMA5	1,2010451	5,31E-07	C11orf87	-0,9371464	2,04E-05
PDE5A	-1,1545425	5,31E-07	PSMD13	0,93286386	2,04E-05
MCM5	-0,901343	5,31E-07	UBXN4	0,63533966	2,04E-05
HJURP	-1,0723456	5,33E-07	GSTM5	0,6988414	2,06E-05
IQGAP3	-1,0396936	5,33E-07	FAM72B	-0,7616915	2,12E-05
NDUFAB1	1,05944663	5,33E-07	EZH2	-0,8728903	2,16E-05
SLC9A9	1,28906576	5,33E-07	SKA3	-1,2808536	2,28E-05
FABP3	-1,2034061	5,33E-07	KIF20B	-1,4650172	2,30E-05
TRIB2	-0,8423257	5,33E-07	TMEM97	-0,7030022	2,32E-05
PLBD2	0,81316299	5,33E-07	XRCC2	-0,9317212	2,34E-05
C16orf93	1,11383327	5,39E-07	AKR1C4	0,78182319	2,38E-05
SMO	0,81350395	5,39E-07	HIST1H4L	-1,1888587	2,38E-05
PQLC2	0,99825613	5,39E-07	SCD	-0,7290473	2,39E-05
CACYBP	0,8800986	5,52E-07	SOD1	0,79886003	2,39E-05
PLEKHB2	0,98908699	5,52E-07	TMEM140	0,65751608	2,39E-05
PSMD4	0,93400209	5,60E-07	SDE2	0,76246341	2,45E-05
ATP6V0D2	0,82963607	5,63E-07	CDC6	-1,0815143	2,47E-05
GLRX3	0,95004097	5,64E-07	CCNF	-0,6643659	2,48E-05
CHORDC1	1,28813282	5,64E-07	DESI1	0,70447774	2,51E-05
UCHL1	0,96799233	5,64E-07	PSMC3IP	-0,7971433	2,51E-05
IL27RA	1,0519705	5,64E-07	MLF1IP	-1,2877631	2,60E-05
COL8A1	-0,8563874	5,65E-07	NFIB	-0,7820289	2,64E-05
POC5	1,17202509	5,65E-07	PSMB1	0,76530354	2,74E-05
OTTHUMG00000	1,35735116	5,67E-07	CLIC2	1,00976916	2,84E-05
SYT1	-0,8874997	5,67E-07	PSMB3	1,03426867	2,85E-05
GAS8	0,89486765	5,67E-07	NXPE3	0,77748267	2,85E-05
PSMC3	0,85618482	5,67E-07	SMC2	-0,9083962	2,85E-05
SDE2	1,2318352	5,67E-07	MYC	0,60327877	2,85E-05
ATP8B1	-0,8509461	5,67E-07	PLAUR	0,59637927	2,92E-05
ACOX2	0,81460871	5,69E-07	PSMA7	0,71336933	2,92E-05
THRB	-0,7924672	5,75E-07	PRLR	-0,7941831	2,92E-05
PTGES	0,90896641	5,76E-07	TXNL1	0,7409499	2,92E-05
FAM217B	0,86010057	5,76E-07	FAM129A	0,85319862	2,97E-05
DIAPH3	-1,3180755	5,76E-07	ATAD5	-1,0281785	2,99E-05
PFN2	0,73213776	5,77E-07	HIST1H2AH	-0,9375047	2,99E-05
TATDN1	1,0213845	5,79E-07	ANXA7	0,74366797	3,06E-05
SLC24A6	0,94193658	5,99E-07	NEK2	-0,7195015	3,06E-05
UBXN4	0,80819362	6,02E-07	GSTO1	0,65552817	3,07E-05
PHTF1	1,30234396	6,02E-07	AKR1C7P	1,10432796	3,07E-05
OTTHUMG00000	2,21069958	6,02E-07	WDR76	-0,7583043	3,07E-05
CITED2	-0,8307992	6,03E-07	ATP6V0A1	0,8227754	3,09E-05
KIAA0368	0,99228298	6,11E-07	RTKN2	-0,8333964	3,09E-05
CAMK2G	0,93979825	6,17E-07	AKR1C3	0,86958167	3,09E-05
HMMR	-1,3160842	6,22E-07	FOLR3	0,76697016	3,09E-05
POPDC3	1,22284448	6,22E-07	LHX9	-0,6843675	3,12E-05
CDCA8	-1,1395516	6,23E-07	MCM8	-1,0216572	3,23E-05
IGHMBP2	0,70232222	6,27E-07	IFNGR1	0,77179467	3,25E-05
TNFRSF11B	-0,7783621	6,33E-07	HIST1H2AJ	-0,6523293	3,32E-05
ULBP2	0,86917041	6,37E-07	E2F6	0,67715776	3,38E-05
INO80B	0,88741682	6,37E-07	FAM72D	-0,9481691	3,42E-05
NAA25	1,4868757	6,37E-07	TRIB1	0,68077374	3,42E-05
ITGAV	-0,7495109	6,39E-07	NREP	-0,6026633	3,43E-05
NEK6	-0,7650738	6,39E-07	KIF14	-1,358277	3,48E-05
ARNT2	-0,8773432	6,44E-07	SLC19A2	0,7207306	3,60E-05
AKR1C2	0,90712367	6,48E-07	USP14	0,66076466	3,72E-05
MX1	1,19902915	6,51E-07	LGR5	-1,1209083	3,75E-05
RRM2	-0,9855295	6,51E-07	HIST1H2AL	-0,7639162	3,83E-05
CDH2	-0,9725394	6,59E-07	EPS15L1	0,74552887	3,85E-05
KIF2C	-1,101485	6,60E-07	COL3A1	-0,7940822	3,95E-05
EPB41	1,02655876	6,62E-07	FAM65B	-0,7770425	3,95E-05
NPLOC4	1,00477684	6,82E-07	SNORD71	-0,6587687	3,95E-05
OXTR	-0,8617182	6,83E-07	ID3	0,70406082	3,95E-05
MAN2B1	0,82039407	6,95E-07	ADPGK	0,61759192	3,95E-05
C6orf165	1,18207862	6,95E-07	PSMA6	0,87336585	3,95E-05
TGFBR1	-0,8458836	6,95E-07	MAMLD1	-0,6982037	3,95E-05
BTG1	-0,7291076	6,95E-07	LINC00707	-1,334459	4,14E-05
UBQLN1	0,75079079	6,95E-07	CDK2	-0,6620709	4,14E-05
KCTD12	-0,8743877	6,95E-07	SLC38A6	0,8108155	4,22E-05
VEZT	0,98707838	6,95E-07	KBTBD8	0,67572051	4,24E-05
GABARAPL1	0,97901497	6,95E-07	BIRC5	-0,7173952	4,26E-05
CBX5	-0,9333126	6,98E-07	SYT1	-0,6227485	4,26E-05
SGIP1	-1,0538413	6,98E-07	DUSP5	0,66723402	4,27E-05
GADD45A	0,79179737	6,98E-07	ALPK2	-0,6565732	4,29E-05
HMGN2P15	-0,7237534	7,05E-07	TM2D2	0,76638767	4,32E-05
ZNF10	0,96821855	7,05E-07	SLC10A7	0,80658744	4,33E-05
AKR1B15	0,89711349	7,05E-07	BRCA1	-0,9073098	4,35E-05

PCNA	-0,9523216	1,32E-06	CEP170B	0,60765082	7,27E-06	FEZ1	0,59474549	3,79E-05	MAP1A	0,64465984	1,78E-05
UBB	0,73889593	1,33E-06	LIMA1	-0,7318384	7,33E-06	IFI44	0,94317694	3,80E-05	FOXQ1	0,65166841	1,78E-05
SQSTM1	0,77531872	1,35E-06	RFFL	0,64415393	7,36E-06	EPHX4	0,94390493	3,89E-05	NIT2	0,79333879	1,78E-05
SPDL1	-0,8434488	1,35E-06	CLCC1	0,95611356	7,46E-06	ALDH1L2	-0,654258	3,94E-05	CCL2	-0,7842633	1,78E-05
PSMD2	0,73512927	1,35E-06	SNORA38B	-1,1300514	7,46E-06	CADPS2	-0,6207024	3,94E-05	FKBP4	0,62011346	1,78E-05
MKX	-0,8054018	1,35E-06	AKAP2	-0,6443773	7,46E-06	LY6K	0,60166344	3,98E-05	CDC6	-1,0798894	1,81E-05
ACOX2	0,7660762	1,37E-06	PITRM1	0,67383078	7,46E-06	PSMA2	0,72495291	3,99E-05	HTATIP2	0,62017297	1,81E-05
IGHMBP2	0,64580759	1,37E-06	LMO7	-0,7660078	7,52E-06	GPR56	0,5862766	4,07E-05	UBE2T	-0,6624724	1,83E-05
AKR1C2	0,89410301	1,37E-06	PRDM8	-0,7197129	7,52E-06	FAXDC2	-0,5864843	4,19E-05	CPNE8	0,85527802	1,88E-05
NAA25	1,40370417	1,37E-06	CALCOCO2	0,62133941	7,52E-06	FOSL1	0,75090021	4,26E-05	TICRR	-1,0645299	1,89E-05
VEZT	0,97463267	1,40E-06	TATDN1	0,86368359	7,52E-06	CLIC2	0,74989433	4,28E-05	ATL1	0,70395962	1,91E-05
OTTHUMG0	1,25076316	1,40E-06	PBX1	-0,7174655	7,54E-06	AMD1	0,69899338	4,41E-05	FEN1	-0,801084	1,92E-05
RBMS3	-0,8717329	1,43E-06	CLN8	0,9073153	7,54E-06	NEK2	-0,61517	4,46E-05	CCNB1	-0,630115	1,95E-05
RAD51AP1	-1,1058108	1,44E-06	PLEKHM3	0,82285805	7,54E-06	PHACTR3	-0,6160395	4,52E-05	C9orf72	0,81562624	1,97E-05
MCM10	-0,850034	1,44E-06	KIF15	-0,9441502	7,58E-06	GPR1	0,79527627	4,55E-05	MMP10	0,80327794	1,97E-05
HMGB1P4	-0,820097	1,44E-06	MAPRE2	0,61414879	7,58E-06	LY96	1,03094685	4,55E-05	SPRY1	-0,6018993	1,99E-05
SLC10A7	1,19765624	1,45E-06	RGCC	-0,6659049	7,63E-06	PTPN22	0,71373388	4,57E-05	GINS2	-0,815407	1,99E-05
PHTF1	1,22963509	1,46E-06	NCLN	0,76913368	7,70E-06	PLA2G16	0,67899172	4,59E-05	TMPO	-0,9340786	1,99E-05
BRIP1	-1,5370718	1,47E-06	VCP	0,67959031	7,75E-06	PTPLAD2	0,68401929	4,61E-05	KCNJ6	-0,8544161	1,99E-05
PSMC3	0,75624347	1,47E-06	TRIM16	0,62434734	7,76E-06	FAM101B	-0,6568317	4,62E-05	BARD1	-0,9599634	2,06E-05
STMN1	-0,8475229	1,47E-06	PSMD2	0,66654653	7,76E-06	HIST1H4J	-0,7273984	4,76E-05	CTNS	0,62369179	2,14E-05
PSMA2	1,05848665	1,47E-06	KCNJ6	-1,3014767	7,79E-06	SNORD71	-0,5963164	4,82E-05	NPLOC4	0,63766632	2,18E-05
DYSF	0,66159209	1,47E-06	GHITM	0,72296526	7,79E-06	ARRB1	0,59554092	4,82E-05	POLQ	-1,1873358	2,18E-05
ZWINT	-0,7733656	1,47E-06	DCLRE1B	-0,6409427	7,79E-06	SORD	0,65661871	4,86E-05	ZWILCH	-0,7648698	2,20E-05
TALDO1	0,91666753	1,48E-06	FAM111B	-1,7214436	7,79E-06	SGOL1	-0,5988912	4,92E-05	TM4SF1	-0,5923873	2,35E-05
DIAPH3	-1,3916567	1,51E-06	WBP2	0,74170304	7,85E-06	BRIP1	-0,8909415	4,93E-05	MED31	0,72726815	2,37E-05
SOCS2-AS1	-0,9360705	1,51E-06	ZDHHC18	0,74106644	7,85E-06	FAM83D	-0,5993264	4,95E-05	HIST1H4B	-0,779779	2,58E-05
ITPKC	0,85525303	1,51E-06	TOP2A	-1,3305499	7,90E-06	SKA1	-0,7077177	5,04E-05	KRT34	-0,7052412	2,65E-05
CBX5	-0,8645634	1,51E-06	LSM10	0,65876869	7,91E-06	KRTAP2-3	0,78683507	5,17E-05	DHFR	-0,5935001	2,70E-05
CCNB2	-1,462151	1,51E-06	KIAA0319	0,74857415	7,93E-06	HSPB6	0,69657392	5,40E-05	HIST1H3C	-1,4077947	2,71E-05
RFTN2	0,89292223	1,51E-06	C3orf72	0,92815138	7,94E-06	MX1	0,69812669	5,40E-05	KNTC1	-0,8907515	2,72E-05
MCM5	-0,869341	1,53E-06	BTG1	-0,7825065	7,97E-06	HIST1H3C	-0,9813754	5,45E-05	C18orf54	-1,0033114	2,78E-05
FAM196B	-0,9283666	1,56E-06	ADAMTS12	0,87064782	8,25E-06	OTTHUMG0	0,69869825	5,87E-05	MCM3	-0,6169383	2,79E-05
CEP170B	0,68503537	1,57E-06	PPARGC1A	-0,7674396	8,39E-06	STRA13	0,71565239	6,05E-05	RTKN2	-0,896462	2,79E-05
CTHRC1	-0,7544837	1,60E-06	PRKAB2	0,63032615	8,48E-06	ZFP36	0,78366485	6,06E-05	SLC10A7	0,87431183	2,80E-05
PDLIM1	-0,747896	1,60E-06	UBR4	0,71922804	8,59E-06	P2RX6P	0,74475425	6,19E-05	PEG10	-0,6143769	2,87E-05
DCTN4	0,74995002	1,60E-06	STYK1	1,30366526	8,61E-06	GAS2L3	-0,869729	6,28E-05	CLIC2	0,88581563	2,89E-05
TMEM140	0,93741913	1,60E-06	MAP3K7CL	-0,6078354	8,63E-06	SNORA38B	-0,9891172	6,49E-05	HIST1H4C	-1,0020269	2,91E-05
PLCE1	-0,7993231	1,60E-06	STRA13	0,80126578	8,63E-06	LOC1005063	0,63090171	6,60E-05	STIL	-0,782857	2,96E-05
NCAPH	-1,0254759	1,61E-06	HECW1	0,79352277	8,63E-06	OPCML	-0,6313678	6,77E-05	RAD51	-0,6815688	2,96E-05
SGSH	0,7577191	1,61E-06	SLC25A38	0,8525371	8,63E-06	TRIP13	-0,6957687	6,79E-05	HIST2H3A	-1,2917945	3,07E-05
HIST1H2AG	-0,9294069	1,62E-06	HGS	0,71476552	8,70E-06	RPSAP52	0,76863932	6,79E-05	ENOX1	0,58855641	3,13E-05
CYTH3	-0,6856487	1,66E-06	BBS4	1,07068235	8,70E-06	USP14	0,59085371	7,02E-05	CDK2	-0,6897135	3,15E-05
HELLS	-1,4233079	1,66E-06	LRIG3	-1,119858	8,70E-06	ABHD3	0,76186954	7,49E-05	HIST1H2BG	-0,7167653	3,15E-05
POMP	0,7159483	1,66E-06	HIST1H3F	-0,9943607	8,76E-06	SPC25	-0,738542	7,72E-05	RFC3	-0,7327488	3,18E-05
ARRB1	0,84500195	1,67E-06	FRMD6	-0,6359075	8,76E-06	ATRX	-0,5875796	8,40E-05	HIST1H2BJ	-0,6061699	3,24E-05
HIST1H2BI	-1,3635082	1,67E-06	POLR3F	0,79812226	8,76E-06	CPNE2	0,67363198	8,56E-05	HIST1H2BO	-0,9850222	3,36E-05
LRRCC1	-1,0618355	1,67E-06	ZFAND5	0,63299287	8,76E-06	TUBA4A	0,71575429	8,58E-05	POC5	0,70720402	3,39E-05
FLYWCH1	0,65024396	1,68E-06	CPNE2	0,86314314	8,76E-06	CPM	-0,7756308	8,60E-05	EPB41	0,59584508	3,40E-05
DEK	-0,8690019	1,70E-06	MPHOSPH10	0,91096501	8,76E-06	SLC12A2	-0,6111224	9,29E-05	GSTM5	0,61377142	3,50E-05
PLAT	-0,7336258	1,70E-06	HFE	0,87436971	8,81E-06	DUSP5	0,6376153	9,31E-05	EML5	-0,9424038	3,51E-05
RNF150	-0,7860199	1,71E-06	FAM43A	-0,8970442	8,85E-06	ARHGAP11E	-0,9593512	9,34E-05	MCM6	-0,7186372	3,52E-05
FBXO5	-1,0736638	1,71E-06	IMPDH2	-0,7142579	8,85E-06	NEFM	0,61355098	9,38E-05	TNFAIP6	0,6233327	3,65E-05
CMTM3	-0,6692613	1,71E-06	ADHFE1	0,74748104	8,88E-06	CCL2	0,60009233	9,53E-05	HMGCS1	-0,664687	3,65E-05
CDCA2	-1,3165771	1,71E-06	H1F0	-0,734195	8,89E-06	ALDH7A1	-0,5855622	9,56E-05	EPHX4	0,63777221	3,78E-05
MXD1	1,08360726	1,72E-06	MEX3B	-0,8213255	8,89E-06	SLC10A7	0,68295544	9,56E-05	KBTBD8	0,62919542	3,83E-05
PCDH18	-0,7094005	1,75E-06	ST13	0,59635178	8,90E-06	PHTF1	0,75755478	9,72E-05	ASF1B	-0,8750989	3,84E-05
NIT2	1,01076806	1,75E-06	HSPB3	0,91601101	8,90E-06	NXPE3	0,59896823	9,80E-05	NCAPD2	-0,6129368	3,84E-05
ST13	0,76194825	1,76E-06	SELK	0,8036744	8,90E-06	MT1F	0,60550402	9,85E-05	LY96	0,86368581	3,88E-05
C11orf87	-1,2210503	1,76E-06	PLEKHG2	-0,6120151	8,90E-06	OTTHUMG0	-0,7107994	0,00010103	MMP12	0,97465788	3,96E-05
HIST1H2BH	-0,7108449	1,77E-06	CREB3L1	-0,6239756	9,05E-06	SAMHD1	-0,5986113	0,00010193	TM2D2	0,66300065	3,97E-05
NREP	-0,8073188	1,77E-06	MN1	-0,8330667	9,07E-06	EVI2B	1,01530182	0,00010236	IFI44	1,06508709	4,01E-05
AGTRAP	0,70092232	1,78E-06	CABLES1	-0,8998073	9,09E-06	SHC3	0,60706886	0,00010616	ITGA2	0,61195069	4,10E-05
LOC1005060	-1,3064111	1,79E-06	BCL2	-1,0179073	9,09E-06	AURKB	-0,6128738	0,00010873	AKR1C7P	1,0562327	4,15E-05
GADD45A	0,71237381	1,79E-06	RPS6KC1	0,83377361	9,12E-06	NEIL3	-0,8147626	0,00011151	INTS10	0,6507197	4,27E-05
SLC25A38	0,92800504	1,79E-06	TBCA	0,7414632	9,16E-06	TMEM171	0,66728547	0,00011162	TMEM140	0,64712661	4,27E-05
SLC9A9	1,12511291	1,79E-06	CRY2	0,66440845	9,16E-06	EPGN	0,90794831	0,00011278	NPR3	-0,728842	4,37E-05
TANGO2	0,70597044	1,79E-06	TMEM57	0,83916903	9,17E-06	HIST1H3G	-0,78614	0,0001128	MXRA5P1	-0,6506113	4,42E-05
VDR	-0,7531163	1,79E-06	AGTRAP	0,5891629	9,29E-06	HIST1H4E	-0,6336388	0,00011582	EGR1	0,87412275	4,62E-05
PGRMC1	0,66836214	1,79E-06	HMGB1P5	-0,7573476	9,31E-06	OCLN	0,68811213	0,00011679	NFIB	-0,6651595	4,75E-05
SGIP1	-0,989094	1,79E-06	ABHD17B	0,87550702	9,36E-06	LMOD1	-0,603464	0,00011679	CENPA	-0,6506842	4,75E-05
AKR1C3	0,829657	1,79E-06	LOXL4	-0,765039	9,36E-06	ITGB3	0,60587158	0,00011788	ASPN	-0,6779752	4,78E-05
LYPD6	-0,6647639	1,79E-06	MPP1	0,73794529	9,36E-06	DNAJC6	0,62152229	0,00012007	MASTL	-0,6731166	4,89E-05
OTTHUMG0	-1,1778712	1,80E-06	IQGAP3	-0,7320684	9,43E-06	NAV3	0,62175089	0,00012078	ID3	0,65229337	5,12E-05
ADH1B	-1,5956617	1,80E-06	DLGAP5	-1,3353371	9,46E-06	CENPE	-0,6673354	0,00012079	SMC4	-0,6544094	5,18E-05
KCTD5	0,80261161	1,80E-06	RARG	-0,6493067	9,49E-06	ABCA9	-0,7379383	0,00012235	HIST1H4D	-1,1544847	5,34E-05
COL3A1	-0,7135757	1,80E-06	HSD17B4	0,73229545	9,62E-06	HIST2H4B	-0,7353773	0,00012307	SDE2	0,67794114	5,39E-05
ADPGK	0,89652539	1,80E-06	SAMD9L	0,79398211	9,62E-06	ABCA6	-0,7384976	0,00012713	HIST1H3H	-1,0398231	5,39E-05
C12orf10	0,78361421	1,80E-06	CDC37L1	0,76073091	9,62E-06	HIST1H4C	-0,9251349	0,00012739	RIPK3	0,62392786	5,39E-05
CCDC15	-0,8221301	1,81E-06	HSPE1	0,66799755	9,64E-06	HIST1H4F	-0,8962482	0,00012739	WDHD1	-0,7683444	5,49E-05
C18orf54	-1,650663	1,83E-06	ARMC9	-0,7246068	9,66E-06	PLEKHH2	-0,6669065	0,00013161	TATDN1	0,60196579	5,54E-05
HTATIP2	0,88209701	1,84E-06	ANP32E	0,8532732	9,69E-06	LOC1001290	-0,6187194	0,00013332	JKAMP	0,58836593	5,54E-05
BICC1	-0,6947152	1,86E-06	HINT1	0,66878579	9,72E-06	TMPO	-0,6067742	0,00013481	SLC19A2	0,6811289	5,61E-05
CDC42EP3	-0,8746865	1,86E-06	MAN2B1	0,62947375	9,88E-06	LOC647859	1,01318745	0,00013666	GLRX	0,62634682	5,62E-05
C16orf93	1,0071875	1,87E-06	OTTHUMG0	1,18325834	9,96E-06	VWA8	0,63475483	0,00013666	MSTN	-0,5947135	5,84E-05

TBCE	0,8984963	7,05E-07	MXRA5P1	-0,8721909	4,35E-05
PSMD2	0,7713377	7,10E-07	HIST1H3D	-0,9876412	4,36E-05
AIMP1	-0,8900322	7,13E-07	TMEM38B	0,87324295	4,41E-05
FBXO5	-1,0300525	7,13E-07	C3orf55	0,79472894	4,41E-05
BRF2	1,51412739	7,26E-07	RNF150	-0,6408376	4,43E-05
HIST1H2AH	-0,8570741	7,35E-07	RIPK3	0,70369113	4,43E-05
KCNQ5	-1,3496057	7,42E-07	OSGIN1	0,67986088	4,55E-05
DCTN4	0,79057257	7,47E-07	TXNIP	-0,6229182	4,55E-05
LRIG3	-1,2233782	7,48E-07	INTS10	0,69891937	4,55E-05
NIT2	1,15430577	7,52E-07	PSMC3	0,59365999	4,55E-05
HTATIP2	0,96153205	7,66E-07	OTTHUMG0	-0,6742748	4,55E-05
PSMB5	0,96941013	7,67E-07	CTH	0,64372234	4,69E-05
SNX21	0,96224019	7,83E-07	HIST1H2AM	-1,1683135	4,69E-05
HSPA4L	1,32653487	7,90E-07	BRCA2	-1,147959	4,71E-05
SNORA29	0,7686261	7,98E-07	SOX4	-0,691402	4,76E-05
HIST1H3J	-1,0899111	8,01E-07	STIL	-0,8831736	4,91E-05
NEK7	-0,9750129	8,06E-07	ZFAND2A	0,89140953	4,93E-05
PTPLAD2	1,04457406	8,06E-07	GPC4	-0,6377778	4,95E-05
HECW1	0,93204365	8,07E-07	BARD1	-0,9437218	4,95E-05
HSPB3	0,97429536	8,11E-07	DMGDH	0,60570555	4,95E-05
OSR2	-1,221683	8,18E-07	CHMP1B	0,65084185	5,41E-05
DYSF	0,70066095	8,22E-07	STOX2	0,71764995	5,60E-05
ARRDC4	1,16192588	8,22E-07	SNORD123	-0,6363578	5,67E-05
CCDC15	-0,7747001	8,22E-07	MASTL	-0,6686002	5,67E-05
POMP	0,76085555	8,27E-07	LARP4	0,61488201	5,67E-05
BABAM1	1,0704504	8,28E-07	EML5	-1,1311952	5,67E-05
AKAP2	-0,7572835	8,31E-07	MNS1	-0,8193201	5,69E-05
SIPA1L1	-0,8522828	8,32E-07	TMPO	-0,8295362	5,84E-05
SLC4A4	-1,239231	8,32E-07	MYLK	-0,7377125	5,86E-05
DCLRE1B	-0,7843365	8,32E-07	EPHX4	0,68949504	5,89E-05
FEZ1	1,04917161	8,43E-07	KIAA1199	-0,715303	5,95E-05
PLAT	-0,7299718	8,43E-07	FABP3	-0,7779938	6,28E-05
KIAA0930	0,76997158	8,50E-07	MMP12	0,98165613	6,31E-05
CDC37L1	0,79538313	8,50E-07	SPC24	-0,6562539	6,50E-05
SPDL1	-0,8010047	8,50E-07	PSMB5	0,71138987	6,74E-05
NUF2	-1,2008322	8,56E-07	HIST2H3A	-1,1287224	6,88E-05
POLR3F	0,87119173	8,56E-07	C9orf72	0,86868627	6,96E-05
GRAMD3	-0,7641388	8,58E-07	PPIF	0,65577048	6,99E-05
NEGR1	-0,9878852	8,58E-07	OTTHUMG0	0,75689583	7,00E-05
SLC45A4	0,78780816	8,58E-07	PDE5A	-0,7279978	7,20E-05
SLC10A7	1,24364236	8,58E-07	ACAT2	-0,7612321	7,23E-05
IDE	0,90876825	8,58E-07	NPR3	-0,856183	7,26E-05
CRY2	0,78638074	8,58E-07	STEAP1	0,58533379	7,43E-05
TRIM59	-0,9137419	8,58E-07	KIAA1524	-0,9872883	7,43E-05
MN1	-1,0844508	8,58E-07	UBXN8	0,5859908	7,43E-05
GPC4	-0,819589	8,85E-07	UHRF1	-0,7206965	7,57E-05
NCLN	1,00447599	8,86E-07	ATL1	0,82136414	7,70E-05
KCNJ6	-1,4376876	8,87E-07	DNAJC6	0,68502148	7,74E-05
HACL1	1,06854618	8,93E-07	HIST1H2BJ	-0,6095544	7,77E-05
DNAJB5	-0,7000271	8,93E-07	AKR1B15	0,59126671	7,77E-05
MAP3K7CL	-0,6972141	8,93E-07	COL8A1	-0,7973348	7,82E-05
TMEM259	0,91861713	9,01E-07	BHLHE40	0,71269526	7,86E-05
IMPDH2	-0,8053328	9,14E-07	RIT1	0,64143892	7,88E-05
TACC2	0,77446425	9,33E-07	PELO	0,64232345	7,94E-05
AFF1	0,8855736	9,39E-07	MT1X	0,94235594	7,94E-05
RTCA	0,94109828	9,40E-07	FEN1	-0,7615272	7,99E-05
H1F0	-0,7468449	9,41E-07	PEG10	-0,717003	8,05E-05
ARHGAP11A	-1,246746	9,46E-07	NPC1	0,72690485	8,22E-05
CDK1	-1,4542556	9,47E-07	JKAMP	0,66038963	8,30E-05
UHRF1	-0,8633358	9,47E-07	MCM3	-0,6205809	8,36E-05
TBCA	0,85195654	9,64E-07	SNORD89	0,65367455	8,45E-05
BBS4	1,12464005	9,66E-07	SLC22A4	0,58801444	8,55E-05
STYK1	1,4322398	9,81E-07	NCAPD2	-0,7078821	8,65E-05
STYX	-0,9123933	9,81E-07	ARHGAP11E	-1,1791294	8,70E-05
CTHRC1	-0,7924559	9,98E-07	MRPL14	0,63382659	8,70E-05
EAF1	0,99844086	1,00E-06	FRY	-0,8752343	8,75E-05
NREP	-0,8229311	1,00E-06	CDKN3	-0,8184237	8,80E-05
PLEKHM3	0,90854596	1,01E-06	OTTHUMG0	0,95229226	8,94E-05
KCNK2	-0,7881791	1,01E-06	GLRX	0,64138449	9,02E-05
NTM	-0,7516513	1,02E-06	BLOC1S2	0,64982166	9,04E-05
GTSE1	-0,7934146	1,03E-06	PHTF1	0,71440468	9,06E-05
BCOR	-0,797302	1,07E-06	UBE2C	-0,7250579	9,27E-05
CDC42EP3	-0,848682	1,07E-06	POC5	0,75578906	9,30E-05
KIF4A	-1,1257983	1,08E-06	C18orf54	-1,0674463	9,30E-05
FAM43A	-1,0098958	1,09E-06	HIST1H2AE	-0,966447	9,30E-05
RAD51AP1	-1,152297	1,12E-06	HIST2H3D	-0,7950386	9,30E-05
CLSPN	-1,3662962	1,12E-06	SMC4	-0,6800211	9,31E-05
DHX32	0,84754157	1,12E-06	CENPA	-0,6452721	9,42E-05
ADPGK	0,99674462	1,12E-06	GHITM	0,61548424	9,64E-05
LHX9	-0,7723247	1,12E-06	HMGCS1	-0,5881529	9,68E-05
SQSTM1	0,79404572	1,12E-06	ERO1LB	0,6529574	9,71E-05
C3orf72	1,11248368	1,12E-06	EMC3	0,68827322	0,00010056
PDGFC	-0,7929174	1,14E-06	TTLL7	0,68816574	0,00010327
CLCC1	1,03636803	1,14E-06	CBX5	-0,7143131	0,00010423
ADRM1	1,01917036	1,14E-06	MCM6	-0,6912134	0,00010474

ITGAV	-0,7577846	1,91E-06	CD99P1	0,61536879	9,98E-06	SLC9A9	0,67768707	0,0001374	SLC38A6	0,72155234	5,94E-05	SPTAN1	0,72681259	1,16E-06	HIST2H2AB	-0,8533987	0,00010489
PAK6	-0,8468431	1,94E-06	CCNE2	-1,2486896	1,01E-05	C18orf54	-0,7661766	0,00014749	LOC1001290	-0,7422592	5,94E-05	RFTN2	0,92871319	1,16E-06	MIR22HG	0,59861041	0,00010549
PSMA1	0,88837696	1,97E-06	TXNRD1	0,618664	1,01E-05	C3orf55	0,72363359	0,00015912	POLA1	-0,8360483	5,96E-05	CYTH3	-0,70889	1,16E-06	IL7R	-0,5908291	0,00011054
TGFBR1	-0,8706182	1,98E-06	APOOL	1,12481189	1,01E-05	CENPI	-0,738777	0,00015939	C11orf87	-0,8449787	6,26E-05	GPSM2	-0,9709339	1,16E-06	CLCC1	0,60904971	0,00011201
MTX1	0,6295805	2,00E-06	ARNT2	-0,7922864	1,01E-05	UBR3	0,60222098	0,00016009	CENPH	-0,7952017	6,26E-05	UGCG	-0,7729026	1,16E-06	LMO7	-0,5930237	0,00011378
MSRB1	0,79320002	2,00E-06	TAF13	0,71769338	1,01E-05	ESCO2	-0,7318895	0,00016162	OPCML	-0,5910891	6,32E-05	TMEM233	0,93600322	1,16E-06	POMP	0,61067965	0,00011378
CNKSR3	0,97509232	2,00E-06	FLYWCH1	0,62395153	1,02E-05	RTKN2	-0,7001491	0,00016476	PSMA2	0,65720296	6,41E-05	DSP	-1,0588936	1,16E-06	AKAP6	-0,7222941	0,00011378
ATP8B1	-0,8771481	2,00E-06	SPATS2	0,86162048	1,02E-05	SCN9A	0,6315279	0,00016956	CCR1	0,66891758	6,44E-05	ADARB1	-0,7555916	1,16E-06	HIST1H4C	-0,9312982	0,00011585
WNT16	-0,9359469	2,04E-06	SOCS2-AS1	-0,7434663	1,03E-05	HIST1H3H	-0,9120922	0,00016956	ARHGAP11E	-1,1186422	6,57E-05	LY96	1,19807645	1,16E-06	SLIT2	-0,6809522	0,00011636
PITRM1	0,69592101	2,04E-06	BLMH	0,77390818	1,03E-05	HIST1H2AG	-0,832018	0,00016991	CENPK	-0,8285908	6,98E-05	PSMA7	0,715292	1,17E-06	CENPE	-1,1277909	0,0001177
PLEKHG2	-0,6120882	2,05E-06	MAMLD1	-0,6620103	1,04E-05	GBP5	-0,6683021	0,00017084	RIT1	0,62772727	6,98E-05	LARP4	0,96182817	1,17E-06	SVEP1	-0,7940944	0,00011773
UBE2M	0,71780607	2,07E-06	PLBD2	0,61425134	1,04E-05	HIST2H3A	-0,8926168	0,00017495	FRY	-0,704011	7,02E-05	HIST1H2AB	-1,3578042	1,17E-06	CSRP2	-0,5989072	0,00011775
AURKB	-0,8809287	2,08E-06	CPNE8	1,08830948	1,04E-05	OTTHUMG0	-0,8558793	0,00017539	SNORD89	0,60546296	7,04E-05	HIST1H2AM	-1,1418258	1,19E-06	OTTHUMG0	-0,8577475	0,00011805
WDR74	0,74210771	2,12E-06	JKAMP	0,83597794	1,04E-05	ATP8B4	-0,6810994	0,00018279	SOX4	-0,722393	7,19E-05	SKA1	-1,1444538	1,19E-06	HMCN1	-1,1453487	0,00011805
LRIG3	-1,2639924	2,12E-06	CADM1	-0,6255896	1,04E-05	PSMC6	0,61094936	0,00018484	OTTHUMG0	-0,6181176	7,19E-05	AURKB	-0,8764279	1,20E-06	OTTHUMG0	0,65959339	0,00011893
FEZ1	0,90535078	2,15E-06	ESCO2	-1,1904898	1,04E-05	TNFSF9	0,65717104	0,0002003	HIST2H3D	-0,7304557	7,19E-05	TTC1	0,83001306	1,21E-06	FAM72C	-0,85715	0,00011906
STMN2	-0,7273197	2,16E-06	AKR1C7P	1,19879081	1,04E-05	HIST2H3D	-0,6082569	0,00022138	FBN2	-0,6561405	7,24E-05	CDC20P1	-1,1871357	1,21E-06	SLC30A1	0,60933564	0,00012552
KAT2B	-0,7527583	2,16E-06	VDR	-0,6741031	1,04E-05	HIST1H2BO	-0,7784621	0,00023801	SVEP1	-0,5959149	7,27E-05	SEMA3A	-0,8995102	1,22E-06	CCL2	-0,5850993	0,00012662
UBXN4	0,76300064	2,17E-06	CASC5	-1,3601649	1,05E-05	HIST1H2BN	-0,6209174	0,00024302	SESN3	-0,7644136	7,37E-05	FAM69A	0,85014394	1,24E-06	OPCML	-0,7089007	0,00012927
STYK1	1,25400845	2,20E-06	CENPA	-0,6044064	1,05E-05	FAM72C	-0,8022795	0,00024522	SLC30A1	0,60776926	7,37E-05	FGD6	0,94817559	1,25E-06	GLRX3	0,65263899	0,00013151
LARP4	0,97599122	2,20E-06	CHRM2	-0,6001093	1,06E-05	GCLM	0,59698851	0,00028621	CLCC1	0,62657383	7,51E-05	PCBD1	0,67941358	1,26E-06	FAT4	-0,8789549	0,00013498
PLEKHM3	0,85619918	2,20E-06	ABCD1	0,60457058	1,07E-05	CCL26	0,71859121	0,00029969	UBR3	0,6104827	7,57E-05	RFTN1	-0,7572876	1,27E-06	PFDN4	0,64682488	0,00014208
CENPA	-0,6899047	2,20E-06	MAF	-0,6335772	1,07E-05	VTRNA1-3	0,70096789	0,00032305	PELO	0,60964853	7,81E-05	HMGN2P18	-0,7591114	1,29E-06	DSP	-0,9785349	0,0001423
CALCOCO2	0,67050606	2,20E-06	MAP2K1	0,70588453	1,08E-05	KIAA1524	-0,6121155	0,00033374	KCNJ2	-0,8824208	7,93E-05	PPID	0,7750261	1,30E-06	FBN2	-0,9545446	0,00014306
RRM2	-1,075731	2,22E-06	KIAA1143	0,93019937	1,08E-05	OTTHUMG0	0,58983802	0,00040305	PHTF1	0,70498365	8,09E-05	FRY	-1,0879623	1,31E-06	CENPK	-0,8020525	0,00014384
ATP8A1	0,96544519	2,24E-06	LOC647859	1,42263119	1,08E-05	PGM2L1	-0,6358292	0,00040701	FAM72D	-1,0592355	8,32E-05	LOC100506013	-1,1380885	1,31E-06	HIST1H3A	-0,8499647	0,00014384
H2AFZ	-0,6861528	2,25E-06	GLRX3	0,73887005	1,08E-05	MIR4521	0,70640873	0,00040845	MT1CP	0,60621002	8,52E-05	CPNE8	1,07199947	1,33E-06	PER3	-0,6867813	0,00014755
DCLRE1B	-0,7589257	2,25E-06	AKR1C4	0,67064031	1,10E-05	STYK1	0,61675815	0,00042629	NAA25	0,79679215	8,62E-05	PDCD4	-0,8145384	1,33E-06	MAGI1	-0,6374311	0,00014906
BRCA2	-1,2857402	2,25E-06	PER3	-0,9330818	1,10E-05	SKA3	-0,5994003	0,00044116	MN1	-0,6433705	8,94E-05	KCNA4	-1,1095617	1,33E-06	SCRT2	0,70222989	0,00014956
AKAP2	-0,6919359	2,25E-06	LOC100130S	-0,7214194	1,11E-05	PDE3B	-0,5992157	0,00046828	OTTHUMG0	0,67474647	8,98E-05	ZNF367	-0,9519056	1,34E-06	KLF10	0,63742431	0,00015014
BNIP3	0,7138396	2,26E-06	PRC1	-1,0383043	1,12E-05	CENPW	-0,5945697	0,00047305	B3GALT2	-0,5892875	9,04E-05	AMOTL1	-0,7299116	1,34E-06	KNTC1	-0,9565762	0,00015052
HMGN2P25	-0,8277711	2,26E-06	UHRF1	-0,7785945	1,13E-05	POLA1	-0,589443	0,00048952	FAM83D	-0,5973931	9,15E-05	ASF1A	0,83037845	1,34E-06	ZWILCH	-0,6840451	0,00015313
TMEM237	-0,8866558	2,27E-06	FXR2	0,62929454	1,13E-05	OTTHUMG0	0,83738775	0,00049295	HIST1H2AL	-0,7541837	9,48E-05	MPP1	0,86914532	1,35E-06	MED31	0,88919087	0,00015359
EMP2	-0,9566125	2,28E-06	ANLN	-1,0918376	1,13E-05	SNORA31	-0,5968777	0,00057436	PTTG1	-0,6852873	9,83E-05	NECAB3	0,68911304	1,35E-06	LOC1001290	-0,8932522	0,00015623
UGCG	-0,921285	2,28E-06	FRY	-1,0002457	1,13E-05	PCDHB12	-0,6098944	0,0005999	RNASEH2A	-0,671597	9,91E-05	MCM10	-0,909765	1,35E-06	DNMT1	-0,6483765	0,00015627
SOX9	-0,9164739	2,31E-06	GAS1	-0,6491107	1,16E-05	PCDHB9	-0,6287385	0,00064003	C3orf55	0,72617089	0,000107	HTR2A	0,80717011	1,35E-06	PPAPDC1B	0,59142042	0,00015627
PSMC2	0,77808664	2,33E-06	FRAS1	-0,7991488	1,16E-05	NAA25	0,6255677	0,00068611	FAM72A	-0,5952691	0,0001109	PITRM1	0,73229667	1,35E-06	MIR222	1,14965975	0,00016147
FRY	-1,0735577	2,33E-06	AJUBA	-0,8639523	1,17E-05	AVPI1	0,59295539	0,00069713	ASF1A	0,60765091	0,00011217	JKAMP	0,92433017	1,36E-06	CUTC	0,58860574	0,00016849
BIRC5	-0,6494455	2,33E-06	ANGPTL1	1,22177641	1,17E-05	IL36B	0,96036258	0,00071218	FAT4	-0,6370303	0,00011396	UBE2O	0,85423168	1,36E-06	RDH11	0,64312156	0,00016854
SMIM10	0,67013656	2,35E-06	KIAA0754	-0,8939027	1,17E-05	TMEM158	0,63794659	0,00073072	PDE5A	-0,6575945	0,00011551	SGSH	0,83130486	1,36E-06	MBNL3	-0,5909729	0,00017046
SHCBP1	-1,5009281	2,35E-06	TTC1	0,76879745	1,18E-05	MAMDC2	-0,5928045	0,00091607	HMCN1	-0,8151674	0,00012976	CEP170B	0,65542363	1,36E-06	ADAMTSL1	-0,618956	0,00017207
SNORA29	0,73320969	2,35E-06	TMEM255A	0,64715414	1,18E-05	SNORD113-1	-0,8467417	0,00099553	OTTHUMG0	-0,6248334	0,00013173	NCAPH	-0,94939	1,36E-06	CCND2	-0,6170404	0,00017786
ZDHHC18	0,77386388	2,35E-06	HSP90AA4P	1,04844861	1,18E-05	MXRA5P1	-0,6003742	0,00104325	MYBL1	-0,6578869	0,00013794	ST13	0,77233895	1,36E-06	CKAP2	-0,8041844	0,00017924
ETV1	-1,0831544	2,39E-06	CAD	-0,5903993	1,18E-05	KCNJ2	-0,616002	0,00104879	RBL1	-0,7609626	0,00014018	TIRAP	0,91990325	1,37E-06	C11orf82	-0,6766893	0,00017924
VAMP1	0,89723868	2,39E-06	GPSM2	-0,981553	1,18E-05	OTTHUMG0	0,85318345	0,00180432	OTTHUMG0	0,65343292	0,00014996	BICC1	-0,7358356	1,38E-06	OTTHUMG0	0,67954777	0,00017926
C10orf107	-0,7015579	2,39E-06	SH2D4A	-0,8152118	1,18E-05	SNORD64	-0,5883071	0,0019192	SPC24	-0,8127526	0,00015176	ORC3	0,97907669	1,39E-06	ADARB1	-0,5962246	0,00017969
TBC1D2	-0,7384158	2,39E-06	EPGN	1,36283085	1,19E-05	LOC1001295	-0,7527692	0,00219047	KIAA1143	0,6075904	0,0001594	TBC1D2	-0,7408299	1,39E-06	HIST1H2AG	-0,9647698	0,00017973
SDE2	1,10426406	2,39E-06	ELAC2	0,76991226	1,19E-05	HIST1H2BB	-0,6383253	0,00383351	OTTHUMG0	-0,744827	0,00015984	HSP90AB3P	0,96254655	1,39E-06	FBN1	-0,7087547	0,00018247
USP14	0,98860453	2,39E-06	NEK7	-0,7368852	1,19E-05	MIR21	-0,6239934	0,00988752	C11orf82	-0,7137377	0,00015987	PCDH18	-0,7165589	1,40E-06	TNFAIP6	0,86546415	0,00018247
MELK	-1,2656744	2,39E-06	SPTAN1	0,62348582	1,19E-05	OTTHUMG0	-0,6032113	0,01026254	BHLHE40	0,66958348	0,00016915	UBR4	0,85045139	1,42E-06	MEIS2	-0,6205664	0,00018626
PDGFRA	-0,8205864	2,39E-06	NDRG3	0,68923871	1,21E-05	SNORD113-1	-0,9148997	0,01131624	CENPQ	-0,7176849	0,00017083	LOC647859	1,72272942	1,42E-06	TATDN1	0,66245925	0,00019299
PFN2	0,64294036	2,42E-06	RFTN2	0,83984702	1,21E-05	HIST1H1D	-0,6034319	0,01277674	CHAF1B	-0,6244481	0,00017224	ATP6V0A1	0,91027874	1,42E-06	CAMLG	0,59200004	0,00019701
DYNC1H1	0,75911256	2,43E-06	HIST1H2BF	-0,8570577	1,25E-05	SNORD114-1	-0,6223854	0,01733763	XRCC2	-0,8742824	0,00019714	SWAP70	-0,7722198	1,42E-06	KCNJ2	-0,9362114	0,00020516
TBCA	0,784979	2,43E-06	HTR2A	0,7049107	1,26E-05	RNU6-51	-0,5941015	0,02399992	DIRAS3	-0,7924656	0,0002009	HMGB1P5	-0,9379605	1,42E-06	IGSF10	-0,6369468	0,00020911
GAS8	0,83655443	2,43E-06	LHX9	-0,6250481	1,27E-05	OTTHUMG0	-0,5956712	0,03047341	FANCB	-0,9850386	0,00021058	TANGO2	0,74246571	1,42E-06	HIST2H2AA	-0,7092592	0,0002124
APOOL	1,16152118	2,44E-06	ADARB1	-0,6360777	1,28E-05	IGKV2-40	-0,7070527	0,03050138	OTTHUMG0	1,11500573	0,00021619	STMN1	-0,7863841	1,44E-06	STYK1	0,68840168	0,0002124
TMEM233	0,92668282	2,44E-06	ITPKC	0,86601839	1,28E-05				STYK1	0,68640999	0,00021802	AP3S2	0,65851957	1,44E-06	NDUFAF4	0,70881153	0,00021335
TRIP13	-0,9824572	2,45E-06	NCAPG	-1,1907046	1,28E-05				GAS2L3	-0,7190759	0,00021992	CCDC47	0,69763605	1,44E-06	FANCB	-0,9971822	0,00021702
RBMS1	-0,6858652	2,46E-06	CCDC47	0,64827352	1,28E-05				OIP5	-0,9059685	0,00024767	MAP3K5	-0,9382784	1,44E-06	TMEM171	0,60845261	0,00021869
HSPA4L	1,18169719	2,46E-06	HIST1H2AE	-1,0782022	1,30E-05				ETV1	-0,6009833	0,00025263	HELLS	-1,4107417	1,45E-06	LY96	1,02993348	0,00022264
MAD2L1	-0,9095828	2,50E-06	KIF2C	-0,7715107	1,32E-05				IL13RA2	0,60586081	0,00025332	KRTAP1-1	-0,7868124	1,45E-06	RAPH1	-0,7657544	0,00022496
TATDN1	0,8804032	2,52E-06	AGTPBP1	1,02622173	1,32E-05				SNORA38B	-0,7870708	0,00027853	RGCC	-0,7872317	1,45E-06	SNORD70	-0,6480496	0,0002381
LDLR	0,81321393	2,52E-06	MIR671	0,68174252	1,32E-05				HTR2B	-0,8116145	0,00028444	VIPAS39	0,97770754	1,49E-06	MN1	-0,7762201	0,00025191
AKR1C4	0,71784787	2,52E-06	FABP3	-0,9440526	1,37E-05				SCRT2	0,6409357	0,00029028	RHOBTB3	-0,806274	1,51E-06	NAA25	0,73723676	0,00025475
H1F0	-0,7791947	2,52E-06	SWAP70	-0,6050182	1,37E-05				ARRDC4	0,58973149	0,00029153	SELK	0,88120522	1,52E-06	PIGH	0,73826208	0,00025567
PHLDB2	-0,7096499	2,54E-06	CHORDC1	0,99501347	1,37E-05				FAM72C	-0,8372222	0,00029426	ARRDC3	-0,7498219	1,52E-06	IKZF5	0,59142949	0,00025975
RTCA	0,90415912	2,58E-06	ATP6V0A1	0,74506739	1,37E-05				OTTHUMG0	0,65736338	0,00030573	GPATCH2L	0,7339747	1,52E-06	NCAPD3	-0,5909734	0,00026007
CDC20P1	-1,3422561	2,67E-06	ARRDC3	-0,6882985	1,37E-05				SNAI1	1,2718228	0,00032457	C3orf55	1,071154	1,52E-06	MIR3198-1	-0,8128869	0,00026304
HSPB1	0,62047076	2,68E-06	TIRAP	0,92242709	1,37E-05				PDE1A	-0,5867876	0,00034495	KCNJ2	-1,572562	1,52E-06	POLA1	-0,8948285	0,00026304
HFE	0,90756449	2,71E-06	NDC80	-1,3500902	1,37E-05				HIST1H2AI	-0,6487378	0,00035332	OTTHUMG00000	1,03500277	1,52E-06	ASF1A	0,71734678	0,00026317
GPSM2	-1,076974	2,71E-06	NAV2	-0,7124624	1,39E-05				OTTHUMG0	0,85460484	0,00036306	AGTPBP1	1,09129285	1,52E-06	WDHD1	-0,7559051	0,00027871
C3orf72	1,04239498	2,71E-06	HIST1H2AB	-1,1887744	1,40E-05				OTTHUMG0	0,84969945	0,00036437	ETV1	-1,0577885	1,53E-06	SESN3	-0,6667806	0,00028311
KCTD12	-0,9320203	2,72E-06	FAM196B	-0,865873	1,41E-05				OTTHUMG0	0,87572142	0,00039483	APOOL	1,19007879	1,54E-06	FAT1	-0,6549543	0,00028821
RHOBTB3	-0,8759726	2,73E-06	SLC48A1	0,63350774	1,41E-05				FAM87B	0,59387976	0,00041545	KRT19	-0,8130095	1,54E-06	ETV1	-0,6044233	0,00029012
PSIP1	-0,7888871	2,73E-06	CENPF	-1,1990983	1,41E-05				CPM	-0,5988761	0,00044135	CEP55	-1,2781835	1,54E-06	COL6A3	-0,6272077	0,00030116
BARD1	-1,1561739	2,76E-06	AMOTL1	-0,6589273	1,41E-05				MIR3198-1	-0,61472	0,00045798	KCTD5	0,83572165	1,54E-06	PRKDC	-0,8465398	0,00030116
GPC4	-0,8008442	2,76E-06	ATMIN	0,64191403	1,41E-05				MMS22L	-0,6876517	0,00050726	SIPA1L2	-0,7646541	1,54E-06	HIST1H4B	-0,6009465	0,00030677
GRAMD3	-0,8191004	2,79E-06	UBE2K	0,68917734	1,41E-05				OTTHUMG0	-0,6573062	0,00052432	PSMA2	1,07232087	1,54E-06	KIFC1	-0,5912028	0,00032252
INTS10	1,11050477	2,82E-06	TGFBR2	-0,6090575	1,42E-05				LOC647859	0,88067647	0,00053712	ERBB2IP	-0,7976945	1,56E-06	CDON	-0,6098953	0,00032876
AGTPBP1	1,02598307	2,84E-06	ARHGAP11A	-1,0519149	1,43E-05				HES1	1,27490227	0,00061465	MIR671	0,85037655	1,57E-06	HIST1H3H	-0,8642088	0,0003288
STYX	-0,9379908	2,84E-06	RHOBTB3	-0,8459288	1,43E-05				IFI30	0,61924184	0,00068515	SCUBE3	-0,8235377	1,57E-06	RNF128	0,62974396	0,00032978
ATP6V0A1	0,88380502	2,86E-06	RNF24	0,63848209	1,43E-05				SPIN4	-0,7737398	0,00071443	OTTHUMG00000	1,91240047	1,58E-06	FAM111A	-0,6299779	0,00033071
ST13P19	0,73049904	2,87E-06	C11orf87	-0,9427502	1,46E-05				KLF10	0,70570468	0,00074705	CAMLG	0,79118499	1,58E-06	OTTHUMG0	-0,8792406	0,00034442
HIST1H2BM	-1,065599	2,90E-06	CDON	-0,9039794	1,47E-05				OTTHUMG0	-0,688994	0,00077303	RDH11	0,9990643	1,58E-06	DIRAS3	-0,6539198	0,00034539
OCLN	1,15426289	2,90E-06	HIST1H3B	-0,8114261	1,47E-05				HIST1H2BB	-0,835089	0,00078196	AIM1	0,85129615	1,59E-06	RFC3	-0,6439922	0,00034909

BCOR	-0,6904285	2,90E-06	TRIM16L	0,64826301	1,47E-05
DNMT1	-0,6804056	2,92E-06	AGTR1	0,88708138	1,47E-05
CHRM2	-0,6979091	2,92E-06	C9orf72	1,45589324	1,47E-05
SKA1	-1,2321372	2,92E-06	KCTD12	-0,752841	1,48E-05
TXNIP	-1,1823495	2,93E-06	PBK	-1,0106778	1,49E-05
L3MBTL2	0,81027652	2,93E-06	HIST1H2AH	-0,6736194	1,49E-05
SQRDL	0,70257195	2,94E-06	CPA6	0,68960569	1,50E-05
HIST1H3C	-1,0287552	2,94E-06	KCNA4	-0,9838035	1,50E-05
STRA13	0,86932939	2,94E-06	ASF1A	0,82387513	1,50E-05
FRAS1	-0,7931057	2,96E-06	PTGES	0,58699497	1,51E-05
LOXL4	-0,8017072	2,96E-06	GCLC	0,70074393	1,51E-05
PLAUR	0,68759094	2,96E-06	SDC4	-0,7674966	1,51E-05
THRB	-0,7694616	2,96E-06	SNX29	0,65241008	1,51E-05
BLM	-0,9149843	2,97E-06	TBCE	0,80548603	1,53E-05
DNAJB5	-0,6343175	2,97E-06	ADH1B	-1,4953576	1,53E-05
MEIS1	-0,7157007	2,99E-06	HJURP	-0,8344847	1,54E-05
MIR671	0,75454941	2,99E-06	RBMS3	-0,7199312	1,55E-05
SDC4	-0,8798475	3,01E-06	GLRX	0,73686404	1,57E-05
HMGB1P5	-0,9349405	3,01E-06	GNAQ	0,61770476	1,57E-05
SCUBE3	-0,7788811	3,01E-06	KIF20A	-0,9834388	1,59E-05
VGLL3	-0,7087618	3,01E-06	DEK	-0,6899226	1,60E-05
HMGN3	-0,7554323	3,05E-06	BICC1	-0,6090634	1,60E-05
SEMA3D	-1,0891595	3,07E-06	DNAJB5	-0,696213	1,60E-05
GPI	0,64765556	3,08E-06	KIF4A	-0,8594626	1,60E-05
KCNJ2	-1,5349618	3,11E-06	ATAD2	-1,1519551	1,60E-05
B3GALT2	-0,6987696	3,12E-06	PLAU	-0,5852123	1,62E-05
HACL1	0,95479787	3,13E-06	CLSPN	-1,1503848	1,65E-05
DDAH1	-0,7272011	3,13E-06	TCEB3	0,64581941	1,70E-05
RAPH1	-0,8156216	3,13E-06	KIAA1715	0,6244775	1,71E-05
BTG1	-0,8322637	3,13E-06	MCM10	-0,7560785	1,72E-05
TCF19	-1,027783	3,13E-06	AAR2	0,76373196	1,72E-05
KIFC1	-0,6612896	3,13E-06	RAPH1	-0,8411538	1,73E-05
CREB3L1	-0,637148	3,13E-06	RRM2	-0,6916405	1,75E-05
PEG10	-0,8887853	3,14E-06	NMT1	0,63189746	1,75E-05
KIAA0319	0,71428513	3,14E-06	SMO	0,62479618	1,76E-05
PDE1A	-1,020106	3,14E-06	TUBA4A	0,95109912	1,76E-05
ATP6V0B	0,81853343	3,14E-06	PDZRN3	-0,6139545	1,76E-05
CCDC47	0,66914893	3,18E-06	CASP1	-0,5850121	1,80E-05
TIRAP	0,83638392	3,22E-06	COL8A1	-0,6065588	1,81E-05
ATMIN	0,71230975	3,24E-06	RNA5SP108	0,74872997	1,82E-05
PBX1	-0,735399	3,25E-06	C1orf52	0,6521674	1,86E-05
GNB4	-0,9173624	3,25E-06	CLIC2	0,99668786	1,86E-05
HUNK	-0,7278867	3,26E-06	NFIB	-0,7463684	1,86E-05
CENPI	-1,3647335	3,27E-06	OCLN	0,97732553	1,87E-05
C14orf1	-0,6822784	3,27E-06	ETV1	-0,9453368	1,87E-05
ERBB2IP	-0,7850518	3,27E-06	HIST1H3J	-0,8357751	1,87E-05
NEGR1	-1,0697049	3,29E-06	RBMS1	-0,626038	1,89E-05
MARK1	0,79080235	3,32E-06	HSPB6	0,68950154	1,89E-05
MAPRE2	0,59439305	3,32E-06	FGD6	0,92282089	1,93E-05
TPM1	-0,6306425	3,33E-06	DNMT1	-0,6305395	1,98E-05
LHX9	-0,7214213	3,37E-06	CDH2	-0,6649013	1,98E-05
LSM10	0,72524375	3,38E-06	ZWINT	-0,61761	2,08E-05
NMT1	0,64214566	3,38E-06	ERBB2IP	-0,6822679	2,08E-05
VWA8	0,96062912	3,38E-06	HMGN2	-0,688645	2,08E-05
SAMD4B	0,7166981	3,42E-06	SPDL1	-0,8035123	2,09E-05
GFRA1	-0,7006725	3,42E-06	PDCD4	-0,7546239	2,09E-05
PSMA7	0,67135289	3,43E-06	ATP5SL	0,64866044	2,09E-05
NECAB3	0,61934222	3,45E-06	DPH3	0,67448592	2,10E-05
ADHFE1	0,80429926	3,48E-06	CAMLG	0,76578873	2,10E-05
ALAS1	0,72143982	3,48E-06	VRK3	0,62264088	2,10E-05
FAM72B	-0,8337155	3,48E-06	OTTHUMG0	1,11727383	2,10E-05
NFIB	-0,862266	3,49E-06	LINC00327	-0,804812	2,10E-05
PLK4	-1,2338138	3,50E-06	INVS	0,75305267	2,10E-05
AMOTL1	-0,6853851	3,51E-06	SPG20	0,70807278	2,12E-05
CDK2	-0,9688503	3,52E-06	RIT1	0,81357677	2,13E-05
SLC46A3	1,12594783	3,53E-06	TMEM237	-0,8059047	2,13E-05
HIST1H1E	-0,6884451	3,54E-06	IKZF5	0,93403648	2,15E-05
MLF1IP	-1,145069	3,54E-06	AIMP1	-0,8072228	2,16E-05
FAM69A	0,85403044	3,56E-06	RNF6	0,61873751	2,16E-05
SPATS2	0,84783142	3,56E-06	PLCE1	-0,7164627	2,17E-05
EZH2	-0,9073456	3,59E-06	LMBRD2	0,82026249	2,18E-05
SIPA1L1	-0,8186708	3,65E-06	CTHRC1	-0,5878192	2,19E-05
YWHAG	0,68588563	3,66E-06	HMGN2P28	-0,6499767	2,24E-05
RPS6KC1	0,84800641	3,67E-06	PRR11	-1,1947698	2,25E-05
CIT	0,75019222	3,67E-06	CDKN2C	-0,5894494	2,25E-05
HGS	0,71161079	3,67E-06	RNF152	-0,7351864	2,25E-05
MNS1	-0,9492017	3,67E-06	API5	0,67159183	2,26E-05
TAX1BP3	-0,5904227	3,69E-06	FBN2	-0,7920833	2,30E-05
MCM7	-0,7755511	3,74E-06	FADD	0,61780599	2,30E-05
KIF20B	-1,2581081	3,76E-06	NEK2	-0,709367	2,30E-05
TK1	-0,6982846	3,77E-06	AURKB	-0,7134166	2,30E-05
KRT19	-0,7414255	3,80E-06	TUBGCP5	0,90174898	2,31E-05
CPA6	0,76486668	3,80E-06	SGK1	0,60177856	2,31E-05
UHRF1	-0,8141309	3,81E-06	DHX32	0,75606721	2,31E-05
PLSCR4	-0,9812291	3,82E-06	CKAP2L	-0,9664754	2,32E-05

SLC2A12	0,699195	0,00081063
MIR4521	0,69021694	0,00119418
PRIM1	-0,6256478	0,00160094
OTTHUMG0	-0,6916907	0,00217594
OTTHUMG0	0,74450919	0,00370227
KCNJ2-AS1	-0,5981744	0,00636932
MIR1245A	-0,8269864	0,00662132
MIR4434	0,80400005	0,00761946
HIST1H1D	-0,6870515	0,00767648
SNORD114-2	-0,6415489	0,00922077
SNORD113-3	-0,6042123	0,022038
IGKV2-40	-0,6780163	0,02251886

CDK2	-0,8854808	1,59E-06	UTRN	-0,8387416	0,0003495
COPS2	0,78026646	1,59E-06	RAD51	-0,6450319	0,00036511
HIST1H1A	0,98967539	1,59E-06	JAGN1	0,6516046	0,00038405
MAFG	0,80137642	1,62E-06	COA6	0,64317284	0,00038489
PCYT1A	0,95613085	1,62E-06	HIST1H2AI	-0,6436101	0,00038489
ZWILCH	-0,9224522	1,62E-06	GINS2	-0,7538543	0,00038489
HMGN3	-0,6958342	1,63E-06	IFI30	0,68546216	0,00038706
MKX	-0,8036431	1,65E-06	GABBR2	-0,6576991	0,0003975
ADH1B	-1,6338109	1,65E-06	KIAA1143	0,60762128	0,00040026
EFCAB7	0,97399194	1,66E-06	FOPNL	0,69231904	0,00043385
PGRMC1	0,72771694	1,66E-06	TENM3	-0,7549949	0,00043478
VGLL3	-0,6869362	1,66E-06	PSMA2	0,7991245	0,00043641
DNM3	0,9141486	1,66E-06	CACHD1	-0,5982541	0,00043859
WDR74	0,81815219	1,67E-06	DPH3	0,59903214	0,00044636
HELB	1,14845136	1,68E-06	OTTHUMG0	0,84596041	0,00044721
NDRG3	0,78651694	1,70E-06	SMC1A	-0,6318185	0,00045271
UBOX5	0,66374276	1,77E-06	FLJ44635	-0,7017743	0,0004572
PDE1A	-0,8496764	1,78E-06	TMEM62	0,58645219	0,0004636
BRCA2	-1,1891395	1,80E-06	FAM87B	0,65357077	0,00049666
PLEKHG2	-0,6346609	1,80E-06	KIF18A	-0,8810853	0,00050203
PSIP1	-0,7826756	1,81E-06	SPIN4	-0,7784298	0,00050949
NR3C1	-0,5931563	1,81E-06	MMP16	-0,66704	0,00053807
IKBKE	0,6193082	1,81E-06	TSPAN2	-0,6189495	0,00054349
TCF19	-0,9119422	1,81E-06	SNORA38B	-1,0165687	0,0005443
GHITM	0,81168263	1,82E-06	HES1	0,84510324	0,00054449
PPM1K	0,94499834	1,82E-06	HSPG2	-0,6382718	0,00054461
BMF	-0,6740406	1,82E-06	KIAA0754	-0,7137979	0,00055863
AKAP6	-0,8871311	1,82E-06	COL5A2	-0,6088341	0,00057121
AKR1C3	0,86062691	1,82E-06	NLRP10	-0,7878904	0,00058408
DDAH1	-0,7450244	1,82E-06	CEP128	-0,6806385	0,00058948
MTHFD2	-0,7790354	1,83E-06	OIP5	-0,809888	0,00060137
NCAPG2	-1,0991126	1,84E-06	SLC2A12	0,72243972	0,0006118
PSMB1	0,71624559	1,84E-06	MMS22L	-0,7975316	0,00061771
ELAC2	0,77909118	1,84E-06	AHNAK	-0,6750807	0,00062345
PHLDB2	-0,712674	1,85E-06	CPM	-0,6295807	0,00064919
RARG	-0,6562637	1,85E-06	RBL1	-0,7691911	0,00064959
GNB4	-0,8972451	1,86E-06	POLR2A	-0,6279131	0,00072515
ELN	-0,8268464	1,87E-06	OTTHUMG0	-0,9257583	0,00072606
RPS6KC1	0,91990689	1,88E-06	MYH10	-0,6697397	0,00073503
TNFSF13B	1,18180917	1,89E-06	PLA2G4A	0,59959218	0,00073552
VWA8	1,02624955	1,89E-06	MSTN	-0,7441579	0,00074602
EML5	-1,0967927	1,89E-06	OTTHUMG0	0,71564023	0,00081138
OTTHUMG00000	-0,7748799	1,91E-06	HIST1H4D	-0,9727475	0,00082408
RAB36	0,70400199	1,91E-06	SNAI1	1,00189891	0,0008407
AGTRAP	0,79424981	1,91E-06	MAD2L1	-0,692069	0,00085798
C14orf1	-0,611009	1,91E-06	LRP1	-0,6739001	0,00088004
LRRCC1	-0,9311657	1,91E-06	CENPH	-0,7418778	0,00089804
CALCOCO2	0,67005361	1,95E-06	OTTHUMG0	0,7252053	0,00099493
SPATS2	0,93248699	1,95E-06	CHAF1B	-0,6025154	0,00099627
PIGH	0,91733715	1,97E-06	OTTHUMG0	0,7030289	0,00100809
EXO1	-1,3239585	1,98E-06	ALKBH2	0,61339249	0,00104587
CPA6	0,79548555	1,99E-06	FRAS1	-0,7196	0,00117714
APOBEC3B	-0,7360708	1,99E-06	OTTHUMG0	0,88375719	0,00123229
TAF6L	0,60391431	2,00E-06	PLEC	-0,7394254	0,0012407
KIAA0319	0,77871134	2,01E-06	IL13RA2	0,61843009	0,00126084
SPG20	0,75004355	2,02E-06	VTRNA1-3	0,59080563	0,0012726
RNF6	0,75242666	2,02E-06	GAS2L3	-0,5949823	0,00128416
KIAA0754	-0,934049	2,02E-06	VCAN-AS1	-0,605539	0,00132121
API5	0,69863787	2,02E-06	CGRRF1	0,62283118	0,001336
PFKM	0,7349128	2,02E-06	ATRX	-0,6618683	0,00138309
MARK1	0,85067149	2,02E-06	EVI2A	0,69637433	0,00143847
BTN2A2	0,66566245	2,04E-06	FASTKD2	0,59485725	0,00144032
KIAA1715	0,67990873	2,04E-06	CYB5R4	0,59616435	0,00151131
IKZF5	0,99295792	2,05E-06	SNORD114-3	-0,8138677	0,00172721
HMGB1	-0,7151076	2,05E-06	MYCBP2	-0,6455865	0,00236489
VDR	-0,7110213	2,05E-06	MIR4521	0,82818139	0,00238783
APOO	0,9610983	2,06E-06	OTTHUMG0	-0,6271003	0,00241815
CTGF	-0,6448723	2,07E-06	OTTHUMG0	0,70365082	0,00250465
BARD1	-1,063453	2,07E-06	OTTHUMG0	-0,9215009	0,00269042
WBP2	0,90713817	2,08E-06	SNORD113-2	-0,8465895	0,00285015
NEK2	-0,884508	2,09E-06	MIR1245A	-1,1916294	0,00361013
TRIP13	-0,8944622	2,09E-06	IGIP	-0,6061805	0,00445434
CCNF	-0,8420235	2,11E-06	OTTHUMG0	-0,7715809	0,00448344
PAK6	-0,7953166	2,12E-06	MIR1299	-0,7997503	0,0045702
IVNS1ABP	-0,7650841	2,13E-06	OTTHUMG0	-0,6943661	0,00617493
ITGA6	-0,8647865	2,13E-06	OTTHUMG0	-0,6315118	0,00629424
STK38	-0,6782676	2,14E-06	LOC647859	0,66278789	0,00688797
RNF150	-0,8120456	2,14E-06	LOC1009968	-0,7957826	0,00771684
SOCS2-AS1	-0,9407895	2,14E-06	SNORD113-1	-0,6115519	0,00858049
MPHOSPH10	0,94685954	2,15E-06	HIST1H1D	-0,6667199	0,00871967
TM4SF1	-0,9224883	2,16E-06	ANKRD36B	-0,6781052	0,00877088
KAT2B	-0,7111634	2,17E-06	ANKRD36	-0,7323663	0,00890423
OTTHUMG00000	-1,2505009	2,19E-06	SNORD113-	-1,138398	0,00901701
INTS4L2	0,73966831	2,21E-06	LMO7-AS1	-0,6521363	0,01194471
TPM1	-0,6467783	2,21E-06	RNU6-51	-1,2209263	0,01196737

VRK3	0,69839062	3,82E-06	GAS2L3	-1,1621475	2,32E-05	PEX19	0,81316653	2,22E-06	SNORD114-	-0,7141287	0,01420509
MAFG	0,70979567	3,82E-06	THRB	-0,6923111	2,32E-05	ABHD17B	0,87350492	2,22E-06	OTTHUMG0	-0,7042514	0,01582543
ANKRD1	-0,818293	3,82E-06	MLKL	0,7161755	2,36E-05	L3MBTL2	0,89468415	2,22E-06	SNORD41	-0,7703153	0,01705912
MEIS2	-0,7953309	3,83E-06	CDC25C	-0,5914397	2,36E-05	ANXA7	0,7618886	2,23E-06	SNORD114-	-0,7367909	0,02020343
MAP2K1	0,71905034	3,84E-06	ATP13A3	-0,6435268	2,36E-05	MCM6	-0,8511691	2,24E-06	OTTHUMG0	-0,6352453	0,02052303
RAB36	0,65874002	3,86E-06	USP38	0,82503944	2,37E-05	VRK3	0,76265154	2,28E-06	MIR21	-0,6958122	0,02277795
ELN	-0,7205904	3,87E-06	FAM69A	0,69706344	2,40E-05	DCBLD2	-0,70604	2,29E-06	LOC1001909	-0,6179594	0,02726147
MPP1	0,77932223	3,87E-06	EXOC8	0,98701312	2,43E-05	DNMT1	-0,7076681	2,30E-06	HIST1H2BB	-0,7086017	0,02744834
AIM1	0,80903315	3,99E-06	SGSH	0,61283218	2,45E-05	SOCS2	-0,7889717	2,31E-06	SNORD114-	-0,608061	0,02907694
MN1	-0,9833885	4,02E-06	RSPO3	-0,7291925	2,46E-05	POLR3E	0,93879837	2,31E-06	OTTHUMG0	-0,6508326	0,03771185
BSCL2	0,6225274	4,02E-06	KIAA0513	0,62754636	2,46E-05	WNT16	-0,8917627	2,31E-06			
TGFBR2	-0,7350363	4,06E-06	SQRDL	0,64423388	2,47E-05	DCK	-0,802845	2,31E-06			
HIST1H4L	-1,0466382	4,06E-06	HOXC8	-0,6542725	2,48E-05	KRT33B	-0,7849952	2,31E-06			
FOXM1	-0,8523557	4,08E-06	ABHD17C	-0,8804701	2,50E-05	SLCO2A1	-0,7458757	2,32E-06			
ORC3	0,94095646	4,09E-06	ADH1C	-0,6148702	2,51E-05	FEN1	-0,7171816	2,33E-06			
STARD5	-0,895805	4,21E-06	KCTD20	0,64906125	2,54E-05	WDR76	-0,7513464	2,33E-06			
KCNK2	-0,8049334	4,22E-06	CCRL1	-0,7164078	2,54E-05	TSPAN2	-1,051139	2,33E-06			
FAM72D	-1,166568	4,24E-06	SLC30A6	0,71439683	2,54E-05	SLC30A6	0,74003282	2,33E-06			
IKBKE	0,62939826	4,24E-06	VPS37A	0,69766451	2,55E-05	PEG10	-0,8149756	2,34E-06			
JMJD6	0,59389127	4,24E-06	TSPAN9	0,60993054	2,55E-05	FBN2	-0,9513875	2,36E-06			
CENPT	0,71128625	4,26E-06	CCDC71L	-0,6648634	2,57E-05	GJA1	-0,6568375	2,37E-06			
CLCC1	0,97342522	4,26E-06	GID4	0,64379321	2,59E-05	RPTOR	0,69401837	2,39E-06			
KCNJ6	-1,4400738	4,26E-06	UGCG	-0,806898	2,59E-05	ARRB1	0,88761552	2,41E-06			
MAP3K7CL	-0,6748603	4,27E-06	HACL1	0,87327357	2,62E-05	POLR3G	0,65334394	2,41E-06			
ALPK2	-0,7086301	4,28E-06	AP3S2	0,60088171	2,62E-05	FANCD2	-0,8837298	2,41E-06			
DACT1	-0,7678728	4,29E-06	MCM7	-0,6713032	2,62E-05	PBX1	-0,7659919	2,41E-06			
TBCE	0,7921238	4,29E-06	CBX5	-0,7244082	2,62E-05	DNAJA3	0,63147149	2,41E-06			
OTTHUMG0	2,27836238	4,31E-06	KIF11	-1,0963135	2,62E-05	HUNK	-0,7359301	2,41E-06			
DTL	-1,0501716	4,31E-06	PDGFC	-0,6083783	2,63E-05	TMEM237	-0,8510339	2,41E-06			
VIPAS39	0,87653348	4,34E-06	EZH2	-0,8205528	2,64E-05	PLAU	-0,6147216	2,41E-06			
CCNF	-0,7796691	4,34E-06	TBC1D2	-0,6160546	2,64E-05	IDH3B	0,66028753	2,42E-06			
TSPAN18	-0,6209266	4,34E-06	SIPA1L1	-0,6879992	2,65E-05	UBTF	-0,586769	2,44E-06			
NFASC	0,64466949	4,35E-06	NPR3	-0,8745634	2,67E-05	SQRDL	0,78122151	2,46E-06			
RFTN1	-0,7376767	4,38E-06	ZNF704	-0,6952738	2,68E-05	BLMH	0,74100397	2,46E-06			
KIAA0513	0,73527745	4,38E-06	C18orf54	-1,2932416	2,69E-05	HSD17B4	0,76105287	2,47E-06			
PDE4B	-0,6749894	4,38E-06	PCNA	-0,7851837	2,73E-05	TOMM40L	0,67378523	2,47E-06			
GHITM	0,73202402	4,40E-06	CDC40	0,74447207	2,75E-05	LDLR	0,79180687	2,50E-06			
ITGA5	0,6577303	4,46E-06	CDK2	-0,8553014	2,76E-05	FLYWCH1	0,6417939	2,55E-06			
CCT3	0,63525708	4,48E-06	ALAS1	0,63320184	2,76E-05	PRDM8	-0,728065	2,56E-06			
ARRDC3	-0,8097042	4,50E-06	FAM20A	-0,697216	2,78E-05	CADM1	-0,7715908	2,56E-06			
GAS1	-0,686852	4,50E-06	MCM6	-0,8285472	2,79E-05	SGK1	0,68404975	2,60E-06			
C4orf46	-0,714982	4,52E-06	SOCS2	-0,7217978	2,80E-05	HMGB1P4	-0,8259117	2,61E-06			
CDC25C	-0,7875196	4,55E-06	GNB4	-0,7728225	2,80E-05	PDE1C	-0,6595018	2,62E-06			
FBN2	-0,9347214	4,55E-06	MAPK9	0,61423394	2,80E-05	GPR137C	-1,0832822	2,62E-06			
HELB	1,0584516	4,55E-06	RUNX2	-0,5987514	2,80E-05	UBE2M	0,80296429	2,62E-06			
FAM217B	0,75187924	4,55E-06	DDB2	-0,5905518	2,81E-05	TAX1BP3	-0,5884876	2,62E-06			
YAP1	-0,6097888	4,57E-06	PLXNC1	-0,6047047	2,81E-05	PSMB6	1,05257217	2,62E-06			
VPS53	0,59836142	4,64E-06	ASPM	-1,5854044	2,82E-05	NGLY1	0,6684863	2,65E-06			
GPATCH2L	0,72911297	4,64E-06	GNPDA2	1,0000964	2,82E-05	TMEM255A	0,79302074	2,65E-06			
HIST1H4D	-1,2525061	4,67E-06	E2F7	-0,8748724	2,83E-05	NFIX	-0,6839204	2,65E-06			
SH3RF1	-0,6711881	4,70E-06	LATS2	-0,5861583	2,84E-05	RBMS1	-0,654774	2,65E-06			
VCAN	-0,6746707	4,74E-06	BNC2	-0,7086556	2,86E-05	TK1	-0,658697	2,66E-06			
TRIM2	0,62158139	4,77E-06	KRT19	-0,6560766	2,86E-05	PPP4R4	0,81236529	2,66E-06			
ABLIM3	0,64438328	4,78E-06	AP4B1	0,81844982	2,86E-05	SLC44A1	-0,7044426	2,67E-06			
PPP1R14BP3	-0,6545686	4,81E-06	STARD5	-0,7357573	2,89E-05	GPR124	-0,687486	2,67E-06			
BRCA1	-0,9929477	4,82E-06	KAT2B	-0,7126894	2,89E-05	NMT1	0,67051493	2,69E-06			
DDAH2	-0,6237754	4,83E-06	EXO1	-1,1625795	2,90E-05	NDNF	-0,6744893	2,69E-06			
GID4	0,74702474	4,84E-06	HIST1H4L	-0,8792017	2,91E-05	CHRM2	-0,7111558	2,70E-06			
ELFN1	-0,6240587	4,89E-06	ASF1B	-0,6244709	2,91E-05	CREB3L1	-0,6959505	2,70E-06			
CD99P1	0,67427329	4,89E-06	SPP1	0,84157901	2,96E-05	GSTM4	0,66376262	2,72E-06			
ARSJ	-1,0615469	4,89E-06	PEX19	0,65846333	2,96E-05	STARD7	0,73760193	2,72E-06			
STARD7	0,70916065	4,89E-06	F2RL2	0,63751077	2,98E-05	DDAH2	-0,63054	2,72E-06			
ELAC2	0,72420706	4,89E-06	SAMD4B	0,5881559	2,98E-05	ZCCHC17	0,84751768	2,73E-06			
ABHD17B	0,77712737	4,89E-06	UBE4B	0,60132767	2,98E-05	UQCC	0,60471846	2,75E-06			
KRTAP1-1	-0,8834593	4,89E-06	HSF2	0,79714134	2,98E-05	EAPP	0,71446014	2,76E-06			
AMOT	-0,7944074	4,92E-06	HMGB1	-0,5991418	3,01E-05	CD99P1	0,66409342	2,76E-06			
PHF19	-0,596697	4,92E-06	DCBLD2	-0,5942256	3,06E-05	HIST1H4L	-1,0889635	2,77E-06			
PPARGC1A	-0,730797	4,99E-06	PDE5A	-0,8346879	3,07E-05	ITPKC	0,94804069	2,77E-06			
SNX29	0,66106557	4,99E-06	NIPAL3	0,63292419	3,07E-05	DEDD2	0,87143064	2,77E-06			
RAD54L	-0,6152948	4,99E-06	TSPAN2	-0,8724276	3,07E-05	RALGPS2	-0,8796166	2,77E-06			
EPGN	1,0768998	5,01E-06	BRIP1	-1,2381371	3,07E-05	TBK1	0,70108979	2,78E-06			
HIST1H1B	-1,1500964	5,01E-06	LIN54	0,67025184	3,07E-05	SLMAP	0,76739851	2,78E-06			
TMEM57	0,83149391	5,01E-06	NYNRIN	-0,5959089	3,07E-05	ZWINT	-0,7781952	2,78E-06			
HIST1H4C	-1,1556928	5,01E-06	HELLS	-1,2361809	3,08E-05	EPGN	1,27742715	2,79E-06			
NDRG3	0,74572761	5,08E-06	NUSAP1	-0,986958	3,08E-05	ARHGEF10	-0,6710976	2,80E-06			
CACHD1	-0,7600915	5,13E-06	CCDC102B	-0,675365	3,08E-05	TSPAN9	0,65939892	2,80E-06			
GSTM4	0,63611099	5,17E-06	LRRCC1	-0,9119174	3,10E-05	ATP13A3	-0,7239046	2,83E-06			
MAP3K5	-0,9114336	5,20E-06	CACHD1	-0,6704087	3,15E-05	NPR3	-0,9583805	2,84E-06			
EXO1	-1,3654294	5,20E-06	C15orf57	0,58878025	3,15E-05	ANKRD1	-0,64415	2,84E-06			
SOCS2	-0,7830858	5,22E-06	DOCK10	0,59692177	3,15E-05	VCP	0,68448329	2,85E-06			
SMC4	-0,8954508	5,23E-06	TMEM171	0,59622698	3,18E-05	SLC38A1	-0,7432063	2,85E-06			
PLAU	-0,5957577	5,26E-06	PHKB	0,76915195	3,19E-05	SETBP1	-0,6991768	2,85E-06			
DOCK10	0,66264366	5,27E-06	ZNF10	0,74240901	3,20E-05	SLC38A4	-0,8619066	2,85E-06			
MLKL	0,6183575	5,27E-06	DACT1	-0,6494546	3,23E-05	HIST1H2AE	-1,3344962	2,87E-06			
C15orf57	0,62130598	5,31E-06	POLR3G	0,6344246	3,24E-05	TALDO1	0,95122273	2,87E-06			

ADARB1	-0,6861717	5,33E-06	ZCCHC17	0,74906612	3,24E-05
EXOSC9	-0,7170527	5,34E-06	SLC9A9	0,86651963	3,26E-05
AKR1C7P	1,20364179	5,34E-06	UBE2MP1	0,62431001	3,34E-05
ITGA6	-0,8795049	5,36E-06	MAFG	0,65556336	3,35E-05
RNF6	0,69586873	5,36E-06	ATP8A1	0,89548178	3,35E-05
SYNPO2	-0,6382193	5,37E-06	INTS4L2	0,69010169	3,35E-05
CARHSP1	-0,7074003	5,39E-06	PHLDB2	-0,7288548	3,35E-05
OTTHUMG0	1,74792922	5,39E-06	CARHSP1	-0,6748369	3,40E-05
SEPP1	-0,5921877	5,41E-06	VWA8	0,81664872	3,41E-05
AKT1S1	0,59693503	5,43E-06	PHLPP1	-0,6532538	3,44E-05
HMGN2	-0,8301437	5,45E-06	NEO1	-0,5950647	3,44E-05
PDCD4	-0,7939771	5,48E-06	TTK	-1,0812694	3,49E-05
SEMA3A	-0,9492107	5,48E-06	BLM	-0,763771	3,50E-05
MAML2	-0,6814564	5,50E-06	PELO	0,75190111	3,51E-05
SGK1	0,66965973	5,51E-06	GRAMD3	-0,6534138	3,51E-05
ATP6V0D2	0,62199556	5,51E-06	CDCA2	-1,0514619	3,52E-05
PRKAB2	0,64780136	5,53E-06	CCNB2	-1,1011642	3,52E-05
CPNE2	0,85529472	5,58E-06	OTTHUMG0	0,87258824	3,56E-05
UBOX5	0,58738537	5,58E-06	MAP3K5	-0,8938677	3,57E-05
SLMAP	0,78344551	5,58E-06	PEG10	-0,6326597	3,57E-05
NIPAL3	0,69891581	5,61E-06	HIST2H4B	-0,772037	3,57E-05
NYNRIN	-0,703592	5,61E-06	SLC4A4	-0,9498808	3,57E-05
RNF152	-0,7233932	5,61E-06	ST13P19	0,62341899	3,57E-05
PLXDC2	-0,5970754	5,61E-06	TRAPPC2P1	0,75280419	3,60E-05
KIAA1143	0,87205648	5,61E-06	RAB30	0,730017	3,62E-05
MAF	-0,6364298	5,64E-06	TENM2	-0,6015809	3,62E-05
LRIG1	0,62398439	5,67E-06	PDE8A	0,69711893	3,66E-05
MUC13	0,68978164	5,67E-06	SGIP1	-0,7676022	3,66E-05
PEX19	0,70914921	5,69E-06	ZNF436	-0,620175	3,67E-05
HIST1H4B	-0,9555374	5,74E-06	KIF23	-1,0179139	3,67E-05
HINT1	0,68555186	5,77E-06	FBXO30	0,77439098	3,77E-05
SIPA1L2	-0,7466599	5,81E-06	STYX	-0,8437412	3,77E-05
TUBA4A	1,01986445	5,81E-06	SMIM10	0,6687903	3,77E-05
GPD2	-0,8857237	5,81E-06	BARD1	-1,0170239	3,80E-05
C9orf72	1,41859218	5,81E-06	PARM1	0,83852031	3,80E-05
NFIX	-0,6211865	5,85E-06	PRMT5	0,58992374	3,86E-05
HIST1H4E	-0,7892211	5,86E-06	HMGN2P18	-0,6720618	3,90E-05
VCP	0,64138247	5,86E-06	TMTC1	0,58816961	3,94E-05
COPS2	0,74137279	6,01E-06	CDC6	-1,0913998	3,98E-05
SPC25	-0,9037026	6,07E-06	TIMELESS	-0,6033132	4,09E-05
FAT4	-0,8216988	6,12E-06	EML5	-0,952625	4,10E-05
PTRF	-0,6261334	6,13E-06	PAK6	-0,6213796	4,12E-05
AP3S2	0,61811142	6,13E-06	TCF19	-0,7365436	4,13E-05
ASF1B	-0,6945343	6,16E-06	SNORD71	-0,5925555	4,16E-05
TNFRSF11B	-0,7472121	6,16E-06	GPATCH2L	0,69277188	4,17E-05
HIST1H2AL	-0,6185177	6,16E-06	RIPK3	0,66930566	4,19E-05
LDB2	-0,7035661	6,16E-06	NEGR1	-0,8072452	4,21E-05
CDC37L1	0,79301073	6,16E-06	SPRY1	-0,7396591	4,21E-05
PRDM8	-0,7631517	6,16E-06	DGKI	-0,6589458	4,22E-05
HSD17B4	0,72363598	6,16E-06	KRBOX4	0,61466993	4,24E-05
ARMC9	-0,6172227	6,16E-06	DCK	-0,7197357	4,24E-05
ADRA2A	-0,7454828	6,16E-06	HMGN2P15	-0,6010041	4,25E-05
JKAMP	0,79499329	6,16E-06	UTP11L	0,59912901	4,26E-05
ANXA7	0,67560682	6,16E-06	CKAP2	-0,7060713	4,29E-05
TMEM255A	0,693325	6,17E-06	C6orf165	0,84102634	4,29E-05
CAD	-0,6258919	6,27E-06	NFYB	-0,800875	4,31E-05
HIST1H1A	0,86970718	6,28E-06	TMEM200A	-0,6077759	4,35E-05
STK32B	-0,663959	6,28E-06	POLR3GL	-0,6008335	4,37E-05
HMCN1	-0,7191302	6,34E-06	TMPO	-0,8879319	4,40E-05
C6orf165	0,90917792	6,37E-06	ARL6IP6	-0,6719578	4,41E-05
EML5	-1,1145668	6,39E-06	HIST1H2AG	-0,6732853	4,43E-05
GREM2	-0,6773712	6,39E-06	FAM72B	-0,6363284	4,43E-05
FGD6	0,90595397	6,40E-06	B3GALT2	-0,5911642	4,51E-05
POC5	1,01855773	6,40E-06	NCAPH	-0,7176997	4,55E-05
FANCD2	-0,9505129	6,54E-06	FAT4	-0,7513113	4,57E-05
USP47	0,65221979	6,56E-06	HMMR	-0,9394943	4,62E-05
ENPP1	-0,6397782	6,59E-06	FGF7	-0,5883537	4,62E-05
MCM6	-0,9394307	6,62E-06	EAPP	0,58838495	4,63E-05
TSPAN9	0,61052533	6,62E-06	GPATCH1	0,67010817	4,64E-05
WDR76	-0,7748552	6,62E-06	TGFBR1	-0,5966126	4,64E-05
TTC1	0,74817252	6,69E-06	TDP2	0,58626732	4,65E-05
INTS4L2	0,69399693	6,72E-06	MCM3	-0,6499689	4,65E-05
MCM3	-0,7215186	6,72E-06	RAD51	-0,6459857	4,65E-05
TOMM40L	0,62895347	6,73E-06	SYNJ1	0,65279355	4,65E-05
RPTOR	0,62818979	6,74E-06	TICRR	-0,8028244	4,69E-05
MAPK9	0,66847119	6,80E-06	DGKH	-0,765351	4,75E-05
PPM1K	0,87978032	6,80E-06	FZD1	-0,7690521	4,75E-05
FXR2	0,62001843	6,85E-06	FAF2	0,59219555	4,75E-05
E2F6	0,89151338	6,98E-06	HIST1H2AM	-0,7992105	4,77E-05
SLCO2A1	-0,6602935	7,02E-06	IVNS1ABP	-0,7834587	4,77E-05
TMEM37	0,7745296	7,04E-06	MCM4	-0,590902	4,79E-05
C3orf55	0,93831734	7,05E-06	MEDAG	-0,6281919	4,84E-05
CDC6	-1,1067263	7,07E-06	PFDN4	0,74503588	4,86E-05
EFCAB7	0,82374787	7,08E-06	SETBP1	-0,5855123	4,89E-05
KIAA0101	-1,096933	7,08E-06	SEMA3A	-0,7964486	4,89E-05

CACHD1	-0,7694681	2,87E-06
ECT2	-0,7691175	2,87E-06
BNIP3	0,72712003	2,87E-06
TRIM2	0,63150768	2,87E-06
PER3	-0,8266483	2,88E-06
TAF13	0,74995563	2,88E-06
EZH2	-0,9298793	2,90E-06
VMP1	0,59569016	2,91E-06
FANCI	-0,9194383	2,95E-06
LMBRD2	0,79455014	2,96E-06
CENPA	-0,7171853	2,96E-06
TRAPPC2P1	0,72779753	2,97E-06
CCT3	0,7067632	2,97E-06
ARHGAP29	-0,6285358	2,99E-06
DTL	-0,9894714	3,00E-06
SDC4	-0,9054145	3,00E-06
CCNE2	-1,2301043	3,03E-06
FAM198B	-0,8319275	3,03E-06
SEMA3E	0,76818377	3,06E-06
ADD3	-0,6938906	3,06E-06
CIT	0,74927484	3,07E-06
PDLIM1	-0,6974863	3,14E-06
GID4	0,72306456	3,14E-06
GPD2	-0,8405639	3,14E-06
ZDHHC18	0,76743739	3,17E-06
C11orf87	-1,1766843	3,17E-06
TGFBR2	-0,6961523	3,20E-06
ENPP1	-0,6615245	3,21E-06
PHKB	0,82379794	3,22E-06
VCAN	-0,7187259	3,22E-06
CDON	-0,8683002	3,22E-06
AAR2	0,91872012	3,22E-06
GCLC	0,72521053	3,22E-06
INTS4L1	0,70055936	3,22E-06
MCM7	-0,7853177	3,23E-06
TRIM16L	0,60678687	3,27E-06
AKT1S1	0,6737299	3,27E-06
MAPK9	0,69492432	3,27E-06
CTNNAL1	-0,8305476	3,27E-06
KIAA0513	0,74316794	3,27E-06
CLIC2	0,9871548	3,30E-06
ITGA5	0,66281606	3,31E-06
NFYB	-0,7812501	3,34E-06
NIPAL3	0,72359108	3,34E-06
SAMD4B	0,71875668	3,34E-06
COL3A1	-0,714087	3,36E-06
DPYD	-0,666316	3,38E-06
LMAN2L	0,84585194	3,40E-06
CAD	-0,6490112	3,42E-06
CDC25C	-0,7266147	3,42E-06
PLXDC2	-0,611219	3,42E-06
BNC2	-0,7341933	3,44E-06
TMPO	-1,0233538	3,47E-06
ABHD17C	-0,9239239	3,48E-06
DDB2	-0,7097425	3,48E-06
NFASC	0,65175984	3,53E-06
TMEM57	0,84254659	3,56E-06
GNPDA2	1,03647079	3,59E-06
ATMIN	0,68539807	3,59E-06
MSRB1	0,81457203	3,61E-06
FXR2	0,68899247	3,61E-06
HINT1	0,67859137	3,61E-06
PTPN21	-0,698337	3,61E-06
LYPD6	-0,6756941	3,66E-06
YWHAG	0,6894037	3,70E-06
HMGN2	-0,6952094	3,71E-06
SYNPO2	-0,6522561	3,71E-06
ALAS1	0,77378923	3,71E-06
ARNTL2	-0,6032248	3,71E-06
BSCL2	0,63968976	3,72E-06
DPCD	0,66210606	3,72E-06
PRMT5	0,6925384	3,76E-06
GFRA1	-0,6291932	3,78E-06
KIAA1143	0,91134661	3,79E-06
IL21-AS1	-0,6853643	3,80E-06
UBE2K	0,68380039	3,85E-06
RASA1	-0,6917601	3,85E-06
TINF2	0,72296642	3,87E-06
MLF1IP	-1,087193	3,88E-06
CENPI	-1,2026508	3,88E-06
ST13P4	0,73548088	3,90E-06
GAS2L3	-1,118715	3,91E-06
HSPA4	0,66435053	3,96E-06
SLC19A2	0,86321823	3,97E-06
MEIS2	-0,7438208	3,97E-06

FAM198B	-0,9133556	7,08E-06	SLC38A4	-0,7661566	4,97E-05
CCL26	0,89077249	7,14E-06	TRAF3IP1	0,6250138	4,97E-05
ATP13A3	-0,6932631	7,14E-06	TMEM167B	0,65092077	4,98E-05
PFKM	0,6427525	7,14E-06	CDK1	-1,1564865	4,99E-05
CDCA3	-0,5958397	7,14E-06	OSBPL3	-0,6578725	5,00E-05
FANCI	-1,0030388	7,14E-06	PLSCR4	-0,6505165	5,02E-05
PDGFC	-0,7899203	7,19E-06	TMEM233	0,60751528	5,20E-05
HTT	0,63776916	7,22E-06	EFTUD1P1	0,61715078	5,23E-05
AGTR1	0,86947243	7,23E-06	TM4SF1	-0,7209295	5,26E-05
PER3	-0,8204536	7,26E-06	ARRB1	0,61141951	5,33E-05
RACGAP1	-0,8549271	7,26E-06	SEMA3D	-0,7520754	5,64E-05
NEK2	-0,8194451	7,26E-06	CDKN2AIP	0,61900599	5,69E-05
SLC44A1	-0,7497811	7,30E-06	NAP1L2	1,24156617	5,69E-05
SPG20	0,66723779	7,30E-06	RAD51AP1	-0,9106516	5,72E-05
INVS	0,75621747	7,48E-06	INTS4L1	0,66931031	5,75E-05
SLC38A4	-0,8816716	7,48E-06	EPG5	-0,6486484	5,76E-05
CCNE2	-1,2417878	7,53E-06	MELK	-0,8773149	5,82E-05
TMEM200A	-0,6607279	7,64E-06	NEK1	0,74625929	5,86E-05
MTHFD2	-0,7780743	7,64E-06	HMCN1	-0,6309286	5,87E-05
KCNA4	-1,1553808	7,65E-06	LMTK2	0,6043176	5,87E-05
ATP10A	-0,5980401	7,65E-06	TTC28	-0,6205772	6,01E-05
HSPB6	0,71871592	7,72E-06	FGD4	-0,705865	6,09E-05
DLC1	-0,6855156	7,73E-06	SHCBP1	-0,9796148	6,10E-05
ZFP36L2	-0,6773494	7,75E-06	TBK1	0,63527386	6,25E-05
FEN1	-0,7279714	7,76E-06	B3GALNT2	0,68509471	6,25E-05
SORT1	-0,7302242	7,76E-06	ANKEF1	0,65770266	6,36E-05
POLR3GL	-0,5911621	7,76E-06	PPM1K	0,84196086	6,41E-05
CYP7B1	-0,8167274	7,76E-06	BNIP3P1	0,77429025	6,47E-05
NCAPD2	-0,6381383	7,83E-06	OTTHUMG0	0,6256795	6,49E-05
RRAS2	-0,6400715	7,88E-06	ATP8B4	-0,7906877	6,49E-05
ARL6IP6	-0,7935734	8,24E-06	SMC2	-0,8588345	6,49E-05
IKZF5	0,92681186	8,34E-06	SLC38A1	-0,675542	6,53E-05
SPC24	-0,6829516	8,36E-06	CCDC109B	-0,5947504	6,58E-05
ZWILCH	-1,0122409	8,39E-06	HIST1H3D	-0,8196571	6,63E-05
AFAP1	-0,6012043	8,42E-06	MEIS2	-0,6554442	6,64E-05
CKAP2	-0,8098755	8,43E-06	STK38	-0,6920885	6,64E-05
TENM2	-0,7320626	8,52E-06	FEN1	-0,6223067	6,66E-05
TMOD1	0,71293621	8,61E-06	SUDS3	0,64198055	6,72E-05
HSPA4	0,62470472	8,61E-06	SLC44A1	-0,5851395	6,75E-05
IVNS1ABP	-0,8315016	8,64E-06	PTPN21	-0,5942709	6,78E-05
CDON	-0,8933773	8,65E-06	PGPEP1	0,62625483	6,83E-05
DDB2	-0,7464703	8,66E-06	ATP8B1	-0,6194235	6,88E-05
DHX32	0,71989962	8,67E-06	ADD3	-0,6263001	6,88E-05
UBE2MP1	0,65807638	8,84E-06	FAM198B	-0,8144526	6,93E-05
CTNNAL1	-0,9374486	8,85E-06	HIVEP2	-0,601443	7,03E-05
CLEC2A	-0,7783937	8,87E-06	TTPAL	-0,6959742	7,06E-05
ZCCHC17	0,7203336	8,87E-06	HUNK	-0,5865462	7,12E-05
PSMB1	0,61071185	8,87E-06	XYLT1	-0,6306949	7,32E-05
CYP3A5	0,59614274	8,87E-06	HIST1H3C	-0,6925226	7,33E-05
NFYB	-0,8583222	8,87E-06	NCAPG2	-0,8556162	7,34E-05
MMP16	-0,7491144	8,87E-06	DBC1	-0,6210585	7,51E-05
PTPLAD2	0,96256014	8,93E-06	GINS2	-0,6433981	7,52E-05
HIST1H2BG	-0,8042895	9,12E-06	LMLN	0,68012077	7,56E-05
SMDT1	0,6641331	9,18E-06	NSUN3	0,62378037	7,56E-05
FGF7	-0,6315308	9,18E-06	PIGH	0,74232928	7,56E-05
FAM20A	-0,7865963	9,18E-06	GABARAPL:	0,68450002	7,75E-05
LMTK2	0,68400038	9,18E-06	GCNT1	-0,6292634	7,75E-05
NOX4	-0,6292679	9,36E-06	NHS	-0,6355052	7,83E-05
PHACTR3	-0,5919737	9,36E-06	WWP1	0,62944122	7,85E-05
FAM72C	-1,0500128	9,37E-06	BRCA1	-0,8642433	7,85E-05
SNRPD3	0,63431112	9,39E-06	RALGPS2	-0,7082476	7,85E-05
OTTHUMG0	-0,6882994	9,45E-06	SLC8A1	-0,6532587	7,91E-05
B3GALTL	-0,7099424	9,45E-06	WDR7	0,66293759	7,91E-05
PTPN21	-0,6556288	9,45E-06	NUF2	-0,949268	7,95E-05
TIMELESS	-0,7404309	9,47E-06	GPC4	-0,5871272	7,95E-05
GPR124	-0,6485094	9,47E-06	HIST1H4C	-0,7982297	7,98E-05
TRAF3IP1	0,63258391	9,47E-06	MBOAT4	0,81805306	8,16E-05
H3F3A	-0,5977693	9,47E-06	TSHZ2	-0,6460768	8,21E-05
ICK	-0,6103007	9,47E-06	PSIP1	-0,6795313	8,26E-05
HMGN2P15	-0,7396364	9,48E-06	UTRN	-0,6204157	8,26E-05
PDE8A	0,71552292	9,82E-06	AFF2	-0,6081667	8,30E-05
NEIL3	-1,0397144	9,83E-06	TET1	-0,5969671	8,60E-05
CENPK	-0,9815541	9,83E-06	HELB	1,0875202	8,80E-05
ATP5SL	0,62844435	9,86E-06	ARSJ	-0,7904282	8,88E-05
LOC1005061	0,62862735	9,86E-06	RACGAP1	-0,6419609	8,90E-05
PELI3	0,58517433	9,86E-06	RBM28	0,65854131	8,97E-05
TNFRSF10A	0,61623487	9,86E-06	EXOSC9	-0,6543681	9,01E-05
GCLC	0,67866771	9,88E-06	GPR137C	-0,8840517	9,01E-05
ARHGAP29	-0,7012873	9,90E-06	ST13P4	0,65644213	9,28E-05
BNC2	-0,7003793	9,93E-06	EML4	-0,7296425	9,33E-05
PHKB	0,75739842	1,01E-05	HMGN2P11	-0,6056375	9,33E-05
CLIC2	0,97426887	1,02E-05	PTPN13	-0,6109199	9,48E-05
HIST2H4B	-0,8437254	1,02E-05	SIM1	-0,6120557	9,54E-05
HSP90AB3P	1,0835979	1,03E-05	NECAP1	0,59461728	9,55E-05
TSPAN2	-1,1358525	1,03E-05	PLK4	-0,9724009	9,63E-05

BLM	-0,8733475	4,04E-06
PRKAB2	0,69052133	4,04E-06
TMEM38B	0,69751286	4,05E-06
SCFD2	-0,6617318	4,06E-06
PLAUR	0,72746487	4,06E-06
NFIB	-0,8560904	4,06E-06
HGS	0,75228641	4,07E-06
MSTN	-0,91876	4,13E-06
TENM2	-0,7248257	4,16E-06
AKR1C4	0,70807276	4,16E-06
RAB30	0,854633	4,21E-06
CASP1	-0,6374468	4,25E-06
SPRY1	-0,6824506	4,30E-06
MAP2K1	0,76387145	4,39E-06
MLKL	0,70744599	4,42E-06
INVS	0,76300998	4,42E-06
GAS1	-0,6732616	4,48E-06
TBC1D22A	0,60080215	4,51E-06
GSTM2	0,64598342	4,59E-06
BCAP31	0,61862531	4,59E-06
ATP6V1H	0,62211027	4,59E-06
SAT1	0,68831932	4,59E-06
SYNJ1	0,77450821	4,60E-06
EPDR1	0,70906526	4,62E-06
PRDX2	0,75158795	4,62E-06
ATP5SL	0,76016204	4,62E-06
USP47	0,65136997	4,70E-06
TMEM200A	-0,6772406	4,77E-06
ERCC8	0,90123568	4,77E-06
EIF1AY	0,74510209	4,83E-06
BRCA1	-0,9498835	4,89E-06
SLC1A2	-0,5945897	4,90E-06
RRAS2	-0,6332475	4,90E-06
RIT1	0,79164157	4,90E-06
ICK	-0,6115356	4,92E-06
KIF20B	-1,1708691	4,92E-06
ROR1	-0,6649103	4,92E-06
MUC13	0,89128049	4,92E-06
LMLN	0,79781765	4,92E-06
ARSJ	-0,9362826	4,93E-06
GPI	0,65557578	4,96E-06
FAM72D	-1,1006805	5,00E-06
VPS53	0,59118677	5,08E-06
PHACTR3	-0,6193961	5,09E-06
BDKRB1	0,6979292	5,10E-06
OXCT2	0,60187599	5,12E-06
YAP1	-0,6200457	5,14E-06
ANKRD39	0,94906548	5,15E-06
PDE8A	0,72656027	5,22E-06
MAF	-0,6479234	5,23E-06
HIVEP2	-0,7081395	5,26E-06
TUBA4A	1,0151255	5,27E-06
HSF2	0,83186541	5,39E-06
SNX29	0,6722412	5,39E-06
STK40	0,59820201	5,39E-06
LIMK2	0,66964514	5,40E-06
PELO	0,78814995	5,45E-06
WDR7	0,74740511	5,45E-06
MRPL14	0,71300114	5,45E-06
PHLPP1	-0,7022955	5,49E-06
ABCF3	0,58957388	5,49E-06
ALPK2	-0,7172144	5,52E-06
DYNC1H1	0,72655577	5,58E-06
TDP2	0,70955758	5,60E-06
NSUN3	0,7195478	5,62E-06
TSPAN18	-0,6779775	5,63E-06
OSBPL3	-0,7176289	5,66E-06
HIST1H2AG	-0,8592748	5,69E-06
USP38	0,81039065	5,75E-06
HOXC8	-0,7382389	5,75E-06
LRIG1	0,63079555	5,79E-06
NRAS	-0,7528402	5,79E-06
TUBGCP5	0,91460139	5,79E-06
CUTC	0,63454475	5,84E-06
FRAS1	-0,8347481	5,84E-06
OSER1	0,90981613	5,84E-06
SMDT1	0,79897599	5,85E-06
PPARGC1A	-0,7312917	5,85E-06
GPATCH1	0,64439638	5,88E-06
ANKEF1	0,721262	5,88E-06
ATP10A	-0,5961923	5,89E-06
PLCE1	-0,7686553	5,89E-06
KIAA0101	-0,9496102	5,89E-06
INTS4	0,72303577	5,89E-06
CDC6	-1,0684725	5,89E-06

NAP1L2	1,35084982	1,03E-05	SKIL	-0,6373797	9,63E-05
ZNF704	-0,7004369	1,03E-05	CTNNAL1	-0,8458882	9,65E-05
POLR3F	0,76508308	1,03E-05	ZNF562	0,67241413	9,71E-05
MRPL14	0,58716564	1,03E-05	P2RX6P	0,62313176	9,74E-05
RBM28	0,700506	1,03E-05	NT5DC3	-0,7018037	9,79E-05
PRICKLE1	-0,5876521	1,03E-05	SMC4	-0,6965665	9,86E-05
CLCN7	0,60326165	1,05E-05	INTS4	0,65138435	9,89E-05
PPIF	0,67047427	1,05E-05	HIST1H3G	-0,7112996	9,95E-05
EPG5	-0,6501042	1,05E-05	RTKN2	-0,7018146	0,00010033
TMEM63B	0,60050206	1,06E-05	AIM1	0,64209384	0,00010071
ASF1A	0,75236619	1,06E-05	MKX	-0,6239148	0,00010075
TBK1	0,64206386	1,06E-05	EMX2	-0,653314	0,00010115
FANCA	-0,5984424	1,06E-05	FASTKD2	0,82505967	0,00010272
IL21-AS1	-0,6090903	1,06E-05	SCRT2	0,89718433	0,00010616
HIST1H3D	-1,1235127	1,07E-05	SYNE1	-0,6526443	0,00010616
FAM72A	-0,6655938	1,07E-05	SLC38A6	0,66937407	0,00010634
HIST1H3G	-0,9292524	1,08E-05	DTL	-0,8116283	0,00010897
HIST1H2AM	-1,1273006	1,08E-05	CEP290	0,84569106	0,00010914
SHOX	-0,656551	1,11E-05	C3orf55	0,84891177	0,00010966
INTS4	0,676607	1,11E-05	CDC20P1	-0,8938249	0,00010976
ARHGEF10	-0,6417963	1,12E-05	TLR6	0,83755075	0,0001121
PLXNC1	-0,60499	1,12E-05	NDUFAF7	0,62699977	0,00011257
TDP2	0,6117715	1,14E-05	CDCA7L	-0,7606217	0,00011301
ABHD17C	-0,9484276	1,14E-05	KDM2A	0,59388791	0,00011396
NOG	-1,0157169	1,14E-05	ZNF277	0,59663374	0,00011398
NEO1	-0,6036267	1,16E-05	SESN3	-0,6411291	0,00011422
PRSS23	-0,6067464	1,16E-05	SNORA10	-0,6509505	0,00011422
ARHGAP11[illegible]	-1,1717903	1,18E-05	IRAK3	0,68003077	0,00011465
LOC730101	-0,9334634	1,19E-05	RNF150	-0,6055092	0,00011619
OSBPL3	-0,7295351	1,24E-05	PTPLAD2	0,6022796	0,00011866
ADAM12	0,59345594	1,24E-05	NNMT	-0,6871279	0,00011919
DPH3	0,71574045	1,24E-05	ATP2B1	-0,6134099	0,00012061
ADD3	-0,753471	1,25E-05	MIB1	-0,5961879	0,00012061
ALDH1L2	-0,7531998	1,25E-05	MSTN	-0,7718119	0,00012218
TBX5-AS1	-0,8103065	1,25E-05	GPD2	-0,7328352	0,00012459
ABCD1	0,61272188	1,25E-05	FOPNL	0,70921027	0,00012479
GJA1	-0,6694292	1,25E-05	KLF3	-0,5852659	0,00012737
RASA1	-0,6455166	1,25E-05	WDR76	-0,6795199	0,00012746
ROR1	-0,6338109	1,26E-05	PDE1A	-0,7149985	0,00012746
API5	0,61874451	1,27E-05	MLF1IP	-0,9232301	0,00012933
AAR2	0,8259441	1,27E-05	MIR214	-0,657151	0,00012985
UBE4B	0,61361905	1,27E-05	EFTUD1	0,59619273	0,00013052
PDE1C	-0,6256869	1,27E-05	CPED1	-0,6893411	0,00013326
RALGPS2	-0,8277835	1,28E-05	MTHFD2	-0,6527816	0,00013378
SESN3	-0,7912087	1,29E-05	MMP16	-0,6846858	0,00013384
UBE2T	-0,6124367	1,30E-05	MNS1	-0,6933413	0,00013473
ECT2	-0,9008224	1,32E-05	EVI2A	-0,6137043	0,00013473
PHLPP1	-0,6823431	1,32E-05	LY96	0,95693399	0,00013686
BLMH	0,70887166	1,34E-05	DPYD	-0,5949834	0,00013943
AAED1	0,61424596	1,34E-05	HSP90AA6P	0,68621077	0,00013943
GNAQ	0,59717007	1,35E-05	ARL13B	-0,6534089	0,00013951
KIAA1715	0,63034915	1,36E-05	ERCC8	0,81576901	0,00014057
FGD4	-0,7347015	1,37E-05	DIAPH3	-0,895214	0,0001414
LIMK2	0,58920446	1,37E-05	TRIM37	0,63131792	0,0001414
SYNJ1	0,73915502	1,38E-05	ZKSCAN3	0,63230234	0,00014233
CABLES1	-0,805284	1,38E-05	CYB5R4	0,7027869	0,00014283
C5	-0,7908776	1,38E-05	MIR21	-0,8460371	0,00014342
PELO	0,72939938	1,38E-05	SKA1	-0,8709116	0,00014345
UBE2K	0,64995899	1,38E-05	NRAS	-0,663884	0,00014447
LIN54	0,6499863	1,38E-05	PDE12	0,72072669	0,00014489
RIT1	0,73949609	1,38E-05	HIST1H1B	-0,8457742	0,00014502
NLRP10	-1,0663945	1,38E-05	APOO	0,68116425	0,00014549
CPED1	-0,7572983	1,40E-05	ZWILCH	-0,7596193	0,00014701
GAS2L3	-1,3272737	1,43E-05	HSDL1	0,59761958	0,00014761
GLRX	0,71016888	1,44E-05	MIR3198-1	-0,6850714	0,00014896
HOXC8	-0,713943	1,45E-05	EFCAB7	0,61631714	0,0001512
DCBLD2	-0,6316737	1,45E-05	NAPB	0,80547537	0,0001527
CENPP	-0,6524959	1,46E-05	ZNF823	0,6397534	0,00015693
STIM2	-0,5912934	1,46E-05	CPM	-0,6879022	0,00015816
CAMLG	0,7263981	1,47E-05	RBL1	-0,849917	0,00015852
G6PC3	0,59256713	1,47E-05	HIST1H4D	-0,7957235	0,00015944
KRT33B	-0,649497	1,47E-05	OTTHUMG0	-0,9956067	0,00016285
NGLY1	0,58987034	1,47E-05	TRPC4	-0,701933	0,00016463
HIVEP2	-0,6562507	1,48E-05	CASP6	0,58562951	0,00016545
ANP32E	0,75848657	1,49E-05	RBBP8	-0,6625005	0,00016617
SLC30A6	0,66945644	1,49E-05	VPS29	0,64639591	0,00016623
OTTHUMG0	0,78797367	1,50E-05	SP4	-0,6194608	0,00017024
FAM43A	-0,9731398	1,50E-05	KIF20B	-0,9083216	0,00017052
KDELC1	-0,6237403	1,51E-05	GK3P	0,80396939	0,00017581
PRMT5	0,60773992	1,52E-05	SNORA31	-0,6615662	0,00017581
BDKRB1	0,63880234	1,52E-05	TRANK1	-0,6563369	0,0001819
PRR16	-0,6993511	1,53E-05	BRCA2	-1,0421829	0,00018192
SLC19A2	0,73845248	1,53E-05	SLFN11	-0,7600654	0,00018205
THBD	0,71010896	1,54E-05	EIF1AY	0,64291383	0,0001842
TBX5	-0,5874035	1,55E-05	HIST1H4B	-0,6312042	0,00018553

MEX3B	-0,8484494	5,95E-06
CDKN2C	-0,6435032	5,98E-06
ATP6V0B	0,93178305	5,98E-06
CMTM3	-0,6226981	5,98E-06
HARS	0,59900626	5,99E-06
OR56B1	-0,5981137	6,00E-06
SLC38A6	0,79547175	6,05E-06
MEIS1	-0,6631796	6,19E-06
HMGN2P28	-0,6802182	6,21E-06
CXCL12	-0,6098592	6,26E-06
LIN54	0,71414034	6,27E-06
OTTHUMG00000	0,80775517	6,32E-06
PCNA	-0,8854386	6,32E-06
TRMT112	0,62342388	6,33E-06
TMEM63B	0,6458067	6,38E-06
GBE1	0,60529395	6,43E-06
SKA3	-0,9847508	6,45E-06
SNAPC5	0,62229677	6,51E-06
RAPH1	-0,8816358	6,51E-06
FAT4	-0,7567579	6,52E-06
PRKCA	-0,6978906	6,56E-06
SLFN11	-0,864293	6,56E-06
YIPF1	0,67234769	6,57E-06
CRBN	0,76576408	6,58E-06
NECAP1	0,70581587	6,60E-06
FANCA	-0,6150865	6,60E-06
MED27	0,60326389	6,62E-06
CLEC2A	-0,6504241	6,65E-06
URM1	0,61929183	6,71E-06
LSM10	0,75442988	6,73E-06
CCDC102B	-0,6787846	6,76E-06
FGD4	-0,6315655	6,78E-06
AFAP1	-0,5982354	6,81E-06
IFNGR1	0,6156269	6,81E-06
NDUFB9	0,68699223	6,89E-06
PLCB4	-0,6107645	6,89E-06
TSHZ3	-0,6043614	6,89E-06
AAED1	0,61705659	6,90E-06
C1orf52	0,74475246	7,02E-06
WISP1	-0,5915627	7,02E-06
ANP32E	0,80341346	7,03E-06
ALDH1L2	-0,8128303	7,03E-06
TNFRSF10A	0,71317192	7,05E-06
ST13P19	0,73217698	7,06E-06
FADD	0,6531709	7,06E-06
DHFR	-0,6234276	7,07E-06
XPR1	-0,5992887	7,10E-06
PRSS23	-0,6207499	7,16E-06
GNAQ	0,60687698	7,16E-06
LIN52	0,62792742	7,24E-06
RNF181	0,65267223	7,25E-06
LDB2	-0,7518181	7,25E-06
ABCD1	0,65315146	7,25E-06
STARD5	-0,7859784	7,32E-06
ZNF197	0,62560795	7,32E-06
AP4B1	0,86479524	7,38E-06
CDC40	0,69618522	7,40E-06
SORT1	-0,6580082	7,45E-06
CPED1	-0,6977401	7,49E-06
FAM196B	-0,8308579	7,49E-06
WWP1	0,66146761	7,53E-06
FOPNL	0,81511747	7,53E-06
MAPRE2	0,62770136	7,54E-06
ARL6IP6	-0,7151779	7,56E-06
MCM3	-0,6815957	7,60E-06
EXOSC9	-0,6774802	7,61E-06
SMIM3	0,666097	7,69E-06
CDCA7L	-0,8337518	7,71E-06
HIST1H4H	0,63486724	7,71E-06
NLRP10	-0,9460765	7,75E-06
PRR16	-0,6527881	7,77E-06
ZNF562	0,78245509	7,85E-06
FAM72B	-0,7719211	7,85E-06
APAF1	-0,8215981	7,85E-06
NEIL3	-0,9666466	7,91E-06
HIBCH	0,81175943	7,94E-06
NAP1L2	1,41894822	7,94E-06
TIMELESS	-0,6910701	7,94E-06
PTRF	-0,6479836	8,04E-06
FAF2	0,58729546	8,06E-06
PPME1	0,63496648	8,06E-06
IRX3	-0,6280001	8,06E-06
KCTD20	0,65911093	8,08E-06
FASTKD2	0,96071665	8,08E-06
PTPN13	-0,6678745	8,08E-06

MCM4	-0,6935663	1,56E-05	SNORD51	-0,6265579	0,00018559
XPR1	-0,6093576	1,56E-05	FOXM1	-0,6078297	0,00018566
ARL13B	-0,8071271	1,56E-05	XRCC2	-0,6490709	0,00018936
B3GALNT2	0,74361844	1,57E-05	LOC1001293	0,59280235	0,00019121
RAD51	-0,718843	1,58E-05	HIST1H4J	-0,6055519	0,00019249
OTTHUMG0	-0,8699773	1,61E-05	ALKBH2	0,60880569	0,00019264
CASP1	-0,6853384	1,62E-05	CLEC2A	-0,5957084	0,0001934
NEK1	0,68096238	1,62E-05	MMS22L	-0,8882206	0,000199
KDM2A	0,62482018	1,63E-05	SUPV3L1	0,62058988	0,00020198
FKBP7	-0,6319473	1,63E-05	HIST1H2BI	-0,9919502	0,00020367
RTKN2	-0,7667681	1,63E-05	MIRLET7I	-0,6320188	0,00020554
INTS4L1	0,61854314	1,63E-05	CENPI	-0,9776482	0,00020771
PTPN13	-0,6491307	1,63E-05	APAF1	-0,6416241	0,00020972
RAD54B	-0,716627	1,63E-05	CARD8	0,60822895	0,00020994
CDKN2C	-0,7754409	1,64E-05	ZNF521	-0,5948093	0,00021325
GPR137C	-1,1427779	1,65E-05	ALS2	0,66108037	0,00021474
DGKI	-0,6530053	1,69E-05	KIAA0101	-0,7515327	0,00021686
LOC647859	1,626631	1,69E-05	ZNF484	0,68658692	0,00022101
SPRY1	-0,6778594	1,69E-05	ITGA6	-0,6187907	0,00022134
AKAP6	-0,7640049	1,69E-05	OTTHUMG0	0,8496284	0,00022325
TMTC1	0,59170021	1,70E-05	ARHGAP11E	-0,9617206	0,00022593
SKIL	-0,653919	1,70E-05	KIAA1841	0,59011511	0,00022748
GSTM2	0,6061476	1,70E-05	HSPA4L	0,76160143	0,00022951
PIGH	0,75985874	1,71E-05	OTTHUMG0	-0,6655494	0,00022951
PLCB4	-0,5876972	1,71E-05	SPC25	-0,6961662	0,00022951
GCNT1	-0,7180028	1,72E-05	L3HYPDH	0,61838077	0,00023271
LMLN	0,71460378	1,72E-05	ZSCAN26	0,59018606	0,00023585
TRPC4	-0,8589185	1,76E-05	SNRNP48	0,63746214	0,00023724
HMGB1	-0,6950178	1,76E-05	TRAPPC2	0,6931546	0,00024348
MSH2	-0,6670555	1,77E-05	POLA1	-0,7975784	0,00024362
KIAA1524	-1,0161663	1,80E-05	OTTHUMG0	0,62949996	0,00024402
USP38	0,74447567	1,80E-05	HOXA13	-0,7884775	0,0002461
OTTHUMG0	1,02471686	1,84E-05	PRKD1	-0,6320559	0,00024863
SLFN11	-0,8746215	1,84E-05	GREB1L	-0,689569	0,00024878
DPYD	-0,7138198	1,86E-05	MSH2	-0,6187054	0,00025072
LMBRD2	0,72697957	1,86E-05	HIST1H3H	-0,7612732	0,00025327
DEPTOR	-0,5934073	1,86E-05	ANKRD1	-0,7045075	0,00025552
ADH1C	-0,6214082	1,88E-05	C18orf25	0,61488041	0,00026582
AFF2	-0,6565495	1,88E-05	TMEM62	0,69598375	0,00026614
BNIP3P1	0,72751336	1,89E-05	ID4	-0,6054273	0,00028168
HIST1H2BO	-0,8274767	1,89E-05	PPP4R4	0,72758436	0,00028209
NPR3	-0,9248468	1,93E-05	QRSL1	0,58628604	0,00028411
DGKH	-0,6993039	1,93E-05	BBS5	0,62302087	0,00028647
EAPP	0,59985751	1,93E-05	FANCI	-0,755673	0,00029262
SIM1	-0,6672269	1,93E-05	DOCK11	-0,7110901	0,00029571
DCK	-0,849982	1,94E-05	CEP55	-0,8651405	0,00029806
CPNE8	1,00400337	1,95E-05	HIST1H2BO	-0,5991633	0,00029842
TAF13	0,68979687	1,96E-05	PLSCR1	-0,6235653	0,00029941
NRAS	-0,8137606	1,98E-05	SPIN4	-0,9920624	0,00030119
CCDC109B	-0,7232993	1,98E-05	GAN	0,69143765	0,00030211
CDCA7L	-0,8793204	2,01E-05	TBX5-AS1	-0,5893637	0,00030408
VPS37A	0,65838926	2,01E-05	FANCD2	-0,6368459	0,00031581
OXCT1	-0,7260177	2,02E-05	ACVR2A	-0,6525187	0,0003269
TCEB3	0,60435506	2,03E-05	SLC25A52	0,59594801	0,00033711
NNMT	-0,6914974	2,05E-05	AS3MT	0,6189404	0,0003439
RIPK3	0,69490594	2,06E-05	SERTAD4	-0,6300147	0,00034476
POLA1	-0,9522723	2,06E-05	WDHD1	-0,7512217	0,00034493
RAB30	0,73098792	2,06E-05	PTX3	-0,7439046	0,00034817
GAB2	0,61829853	2,06E-05	RAB27B	-0,6016736	0,00034892
LMAN2L	0,69111297	2,06E-05	CCL2	-0,6706384	0,00036138
RBMS2	-0,6096765	2,07E-05	SNORD114-	-0,8161606	0,00036571
RFC3	-0,6668538	2,07E-05	C5	-0,6420148	0,00038133
EPDR1	0,60553648	2,08E-05	LIN9	-0,6039808	0,00042285
CEP290	0,89159695	2,08E-05	RNASEL	0,62548031	0,00042638
BBS4	1,0117163	2,10E-05	ZNF222	0,68954252	0,00042868
TRPC6	-0,8865034	2,11E-05	HMGN2P25	-0,6074383	0,00043054
C1orf52	0,65813326	2,12E-05	ZNF234	0,62004274	0,00043626
SCRT2	0,84825079	2,12E-05	POLQ	-0,6791847	0,00043943
SKA3	-1,1287202	2,14E-05	ECT2	-0,6427099	0,00044631
SCFD2	-0,6441637	2,15E-05	SIRT1	-0,6213526	0,00045062
TRAPPC2P1	0,7413087	2,16E-05	SNORD114-	-0,8706541	0,00045278
ANKEF1	0,62164971	2,16E-05	KIAA0922	-0,6167271	0,00046213
RBL1	-1,0132109	2,17E-05	LRRC37B	0,6641642	0,00046697
CCDC71L	-0,7788845	2,17E-05	CYP7B1	-0,6670957	0,00046732
NT5DC3	-0,7557783	2,18E-05	NLRP10	-0,7235001	0,00048482
CDH8	-0,6691741	2,18E-05	SKA3	-0,7801236	0,00049428
PRKD1	-0,7347092	2,18E-05	KIAA1524	-0,7292314	0,00053099
FAM111A	-0,7111383	2,19E-05	HIST1H4F	-0,6983015	0,00054064
HMGN2P11	-0,7191784	2,20E-05	FAM72D	-0,8598215	0,0005755
GSTCD	-0,737448	2,21E-05	RNA5SP385	-0,8186037	0,00057971
DNAJA4	0,59272367	2,21E-05	AQP9	-0,6836893	0,00058029
SLC8A1	-0,6939469	2,24E-05	NHLRC1	0,8125575	0,00061176
SLC7A1	-0,5938899	2,24E-05	SNORD113-	-0,9444116	0,00062989
HIST1H2AJ	-0,5866219	2,26E-05	FAM72C	-0,7468577	0,00063982
SYNE1	-0,6336324	2,28E-05	LOC1005058	0,63372148	0,00063982

SIM1	-0,6432681	8,13E-06
TRPC6	-0,7752632	8,13E-06
SNRPA1	0,63234164	8,13E-06
GALNT5	-0,5964383	8,13E-06
DOCK10	0,60943023	8,29E-06
MMP16	-0,7083597	8,34E-06
KIFC1	-0,6511282	8,37E-06
HSPB1	0,62976426	8,38E-06
CKAP2	-0,7244325	8,40E-06
BCL2	-0,9982318	8,40E-06
DDX27	0,63798238	8,40E-06
ARL13B	-0,7594551	8,41E-06
B3GALNT2	0,78005224	8,42E-06
C12orf10	0,84720116	8,50E-06
ZKSCAN3	0,68019898	8,51E-06
COASY	0,69896449	8,51E-06
CABLES1	-0,8045248	8,51E-06
ELFN1	-0,6588849	8,53E-06
MRPL45	0,65042442	8,53E-06
RAD54L	-0,6175272	8,53E-06
NHS	-0,7772554	8,55E-06
PLK4	-1,1151146	8,57E-06
ZNF704	-0,6959331	8,57E-06
LOC100129361	0,67853043	8,72E-06
HIST1H3D	-1,0206683	8,75E-06
SUPV3L1	0,72500443	8,92E-06
USP1	-0,6225189	8,95E-06
UBE4B	0,60749274	8,96E-06
AKR1C7P	1,27290725	8,98E-06
BIRC5	-0,6054926	9,02E-06
TLR6	0,93776624	9,03E-06
FAN1	0,61364025	9,07E-06
TBX5-AS1	-0,6979923	9,15E-06
ID4	-0,727387	9,15E-06
MARCH3	-0,6007128	9,21E-06
FOXM1	-0,7416487	9,23E-06
XRCC2	-0,8784986	9,25E-06
TRPC4	-0,8440998	9,27E-06
STK32B	-0,5977789	9,28E-06
HDHD3	0,69448471	9,28E-06
OTTHUMG0000	0,75944684	9,34E-06
LSG1	0,58985426	9,38E-06
ATP8B4	-0,9166843	9,40E-06
CENPP	-0,682772	9,46E-06
VPS37A	0,6606431	9,46E-06
LMTK2	0,68275325	9,63E-06
CLCN7	0,67794354	9,74E-06
MAD2L1	-0,6960475	9,78E-06
DRG1	0,59429925	9,87E-06
HSPB6	0,72815257	9,93E-06
EFTUD1P1	0,68786078	9,95E-06
HIST1H4C	-0,9820493	9,95E-06
SPC25	-0,85383	9,97E-06
UQCRC2	0,59403787	1,00E-05
PNPLA8	0,59143631	1,00E-05
HMCN1	-0,7468577	1,00E-05
RACGAP1	-0,7046806	1,00E-05
MCM2	-0,6208374	1,00E-05
MAML2	-0,6429439	1,01E-05
PSMD8	0,82959026	1,01E-05
TMEM167B	0,6501971	1,02E-05
CDK15	-0,641685	1,03E-05
UBE2MP1	0,70132087	1,03E-05
KRBOX4	0,70825941	1,05E-05
EMC3	0,6097565	1,05E-05
NEO1	-0,6117114	1,07E-05
KIAA0922	-0,7674508	1,07E-05
GLCE	-0,6358435	1,07E-05
FAM20A	-0,7613898	1,07E-05
BCL6	0,5954206	1,07E-05
TUBB2A	0,59473753	1,07E-05
HSDL1	0,68757551	1,07E-05
CENPK	-0,878286	1,07E-05
ABCA6	-0,9314535	1,07E-05
PPP1R14BP3	-0,7114619	1,08E-05
KLF7	0,5960932	1,08E-05
ZNF484	0,78615212	1,08E-05
AKAP8L	0,61355724	1,09E-05
ABLIM3	0,58881788	1,10E-05
LOXL4	-0,7384181	1,11E-05
HTT	0,6421828	1,11E-05
ACTRT3	0,77380332	1,12E-05
RUNX2	-0,622677	1,14E-05
GREM2	-0,6690705	1,15E-05
NYNRIN	-0,6597976	1,15E-05

SAMD9L	0,6048096	2,28E-05	MCM8	-0,5934727	0,00065547
CCDC102B	-0,7297946	2,28E-05	LOC1005055	-0,6100147	0,00066809
GLRX3	0,85094167	2,28E-05	MGC24103	-0,8495593	0,00074114
EML4	-0,6948636	2,28E-05	TXNDC17	-0,611636	0,00074186
PRKCA	-0,6380023	2,30E-05	ZNF175	0,60429829	0,00078084
DNM3	0,7412379	2,30E-05	CHML	-0,8770876	0,00085961
RAD18	-0,5905911	2,34E-05	NEIL3	-0,6325709	0,00088036
RNASEH2A	-0,7565504	2,36E-05	MIR4668	-0,5960299	0,00088711
MCM8	-0,8023816	2,37E-05	ALG10	-0,6498189	0,00089727
NHS	-0,6757232	2,40E-05	DEPDC7	-0,6435504	0,00101528
NAPB	0,79460875	2,41E-05	CAPS2	0,59626072	0,00105582
LOC1001293	0,58570564	2,41E-05	DPY19L2	-0,6319314	0,00108926
CAMKK1	0,70527476	2,44E-05	INHBB	-0,7120753	0,00112347
ASPN	-0,7068209	2,47E-05	ABCA6	-0,7675936	0,00113897
WDR7	0,63664763	2,50E-05	OTTHUMG0	-0,6006693	0,00114711
F2RL2	0,64253473	2,50E-05	PGM2	-0,6287393	0,001175
P2RX6P	0,72216582	2,52E-05	DDX60	-0,6265478	0,00118565
ID4	-0,704806	2,54E-05	CENPK	-0,7146893	0,00124483
SAMHD1	-0,750172	2,56E-05	OTTHUMG0	-0,6432018	0,00124753
CDK15	-0,6056965	2,56E-05	OTTHUMG0	0,64034703	0,00126511
PPME1	0,58616026	2,58E-05	CCP110	-0,5978656	0,00128026
MTFR2	-0,6973634	2,59E-05	KCNJ2-AS1	-0,7027393	0,001614
STK38	-0,7058756	2,59E-05	SNORD113-	-1,1098102	0,00179137
RSPO3	-0,5897865	2,63E-05	PRIM1	-0,6888886	0,00184645
RIN2	-0,5928423	2,64E-05	EVI2B	-0,610951	0,00187406
MBOAT1	-0,7679786	2,66E-05	FANCB	-0,7795361	0,00211323
OR56B1	-0,6586403	2,69E-05	USP46-AS1	-0,5850269	0,00235845
SETBP1	-0,6355215	2,70E-05	OTTHUMG0	-0,5901235	0,00246725
CA5B	-0,6044388	2,70E-05	IGIP	-0,6415689	0,00250526
GLCE	-0,7230383	2,71E-05	MIR4720	0,85244513	0,00253618
MEX3B	-0,8886311	2,73E-05	OTTHUMG0	0,70912626	0,00263794
PRPS2	-0,617843	2,76E-05	CENPQ	-0,6396704	0,00283636
GINS2	-0,6872011	2,78E-05	SNORD114-	-0,5943002	0,00292914
ZNF197	0,5985938	2,81E-05	MTBP	-0,5879671	0,00294767
CENPW	-0,7088853	2,82E-05	SNORD56B	-0,7316729	0,00296103
CYB561D2	0,59107685	2,85E-05	LOC643401	-0,6416112	0,00313107
SPIN4	-1,0733833	2,88E-05	MRO	0,60458571	0,00390976
SLC38A1	-0,6682589	2,88E-05	LOC1001295	-0,8159231	0,00397446
GPATCH1	0,61338526	2,89E-05	OTTHUMG0	0,64904089	0,00422292
MMS22L	-0,9679867	2,91E-05	RNU5D-1	0,65120318	0,00450586
TUBGCP5	0,87556137	2,93E-05	MIR1244-1	-0,6048401	0,00584859
ZNF562	0,69844343	2,96E-05	BLID	-0,6151358	0,00697781
EFTUD1	0,62871371	2,98E-05	LMO7-AS1	-0,6738471	0,00758967
DES	0,64052144	2,99E-05	RNU6-51	-0,7183134	0,01009256
CC2D2A	-0,6160203	3,01E-05	HIST1H1D	-0,6202498	0,012855
HIST2H2AB	-0,8311876	3,02E-05	MIR553	0,726669	0,01500632
ABCA6	-0,9520017	3,02E-05	OTTHUMG0	-0,6104186	0,01558197
SUDS3	0,63408825	3,08E-05	OTTHUMG0	-0,6176026	0,01569888
CPM	-0,8495251	3,10E-05	SNORD114-	-0,5973881	0,0195053
OTTHUMG0	-0,922234	3,13E-05	IGKV2-40	-0,599977	0,04347823
DOCK2	-0,6584185	3,13E-05			
HTR2A	0,69646949	3,18E-05			
MID2	0,70583362	3,18E-05			
WDHD1	-0,8807096	3,18E-05			
ALG10	-0,7712051	3,21E-05			
LY96	0,93564689	3,22E-05			
PGPEP1	0,70743688	3,22E-05			
CDC40	0,61835769	3,24E-05			
DIAPH2	-0,616506	3,24E-05			
SPR	0,6054328	3,24E-05			
SEMA3E	0,61118194	3,28E-05			
PGM2	-0,7452172	3,28E-05			
HNMT	-0,6311815	3,28E-05			
EIF1AY	0,61322972	3,30E-05			
COASY	0,59170352	3,30E-05			
PTX3	-0,7581497	3,30E-05			
TICRR	-0,7750878	3,32E-05			
EFTUD1P1	0,62209617	3,34E-05			
NDUFAF7	0,60771114	3,35E-05			
NDNF	-0,599377	3,42E-05			
PPID	0,63012055	3,42E-05			
UBE2C	-0,7424396	3,42E-05			
ERCC8	0,81642083	3,43E-05			
LCLAT1	-0,672678	3,43E-05			
GALNT5	-0,5990486	3,43E-05			
OTTHUMG0	0,60539189	3,44E-05			
MIR3198-1	-0,887235	3,44E-05			
XYLT1	-0,6333058	3,44E-05			
MBOAT4	0,82321333	3,54E-05			
PCDH12	0,75333015	3,55E-05			
FAM101B	-0,5995595	3,55E-05			
EMX2	-0,6755734	3,56E-05			
USP1	-0,6655619	3,57E-05			
PRRX2	-0,5971086	3,62E-05			
CTSC	-0,670943	3,66E-05			

TNFRSF10D	0,71293774	1,15E-05
MCM4	-0,6757305	1,15E-05
SUDS3	0,67752287	1,15E-05
RIPK3	0,78082137	1,15E-05
G6PC3	0,6193745	1,16E-05
TMTC1	0,59322694	1,16E-05
WDHD1	-0,8049354	1,17E-05
THBD	0,71091314	1,17E-05
TLR3	-0,5859188	1,17E-05
SKP2	-0,5928061	1,17E-05
C5	-0,7126826	1,18E-05
FARSA	0,60728137	1,18E-05
DACT1	-0,7064001	1,18E-05
SNRPD3	0,65884178	1,18E-05
MNS1	-0,7194024	1,20E-05
NT5DC3	-0,7594102	1,20E-05
LCLAT1	-0,6238552	1,20E-05
EPG5	-0,6138059	1,21E-05
S1PR3	-0,6005963	1,21E-05
CPM	-0,884064	1,22E-05
NDUFAF7	0,67305572	1,22E-05
HIST2H2AB	-0,8187096	1,22E-05
CPNE2	0,86228226	1,22E-05
GCNT1	-0,643696	1,22E-05
RNF152	-0,6295892	1,22E-05
SCPEP1	0,60638408	1,23E-05
C15orf57	0,6942636	1,24E-05
LIN9	-0,682284	1,26E-05
BNIP3P1	0,82683572	1,28E-05
TRAF3IP1	0,60342108	1,29E-05
CASP4	0,59456567	1,29E-05
DPH3	0,78121591	1,30E-05
HIST1H4D	-1,0631711	1,30E-05
SLC8A1	-0,7054067	1,30E-05
GABARAPL3	0,61715163	1,30E-05
MCM8	-0,7686427	1,30E-05
HIST2H4B	-0,7133425	1,31E-05
E2F7	-0,6907313	1,31E-05
EFTUD1	0,68320492	1,31E-05
CARD8	0,66933201	1,31E-05
TMEM62	0,85167929	1,33E-05
ZNF277	0,67575705	1,33E-05
CDKN2AIP	0,6200997	1,33E-05
EPHA4	-0,6034985	1,33E-05
PLXNC1	-0,6010355	1,33E-05
EXOC8	1,01755641	1,33E-05
PTDSS2	0,62292246	1,33E-05
MFSD8	0,65938376	1,33E-05
POLR2C	0,87179869	1,33E-05
LOC550643	0,62041057	1,34E-05
SLC7A1	-0,6516079	1,36E-05
NDUFA4	0,67190817	1,37E-05
FMN2	-0,5958397	1,39E-05
ARHGAP11B	-1,1800993	1,39E-05
NNMT	-0,6209491	1,39E-05
PPIF	0,68154789	1,40E-05
RBL1	-0,9201521	1,40E-05
RBM28	0,67690229	1,41E-05
GLRX	0,762907	1,42E-05
TMOD1	0,69494884	1,43E-05
KCNIP3	-0,5946076	1,44E-05
CEP70	0,65080399	1,44E-05
SMC4	-0,8289124	1,44E-05
MSH2	-0,6006474	1,46E-05
HMGN2P25	-0,7263824	1,47E-05
KIF24	-0,609728	1,47E-05
NPR2	0,64645135	1,47E-05
EML4	-0,6553405	1,48E-05
CYB5R4	0,7782342	1,49E-05
ATAD5	-0,6893033	1,49E-05
MMP12	1,00621531	1,50E-05
ERO1LB	0,76832295	1,51E-05
MBOAT1	-0,8001806	1,51E-05
GPATCH3	0,60939851	1,52E-05
TM2D3	0,60588859	1,52E-05
PNLIPRP3	0,63708957	1,53E-05
HSP90AA4P	1,30793373	1,54E-05
ATP6V1D	0,645683	1,59E-05
KIAA1524	-0,9311484	1,60E-05
TRAPPC2	0,68802405	1,60E-05
POMT2	0,63906467	1,61E-05
GFM1	0,62614134	1,61E-05
AGTR1	0,88193904	1,62E-05
FZD1	-0,8055313	1,62E-05
UBE2C	-0,677116	1,62E-05

APOO	0,81162283	3,66E-05
LIN9	-0,7224181	3,66E-05
DHFR	-0,662726	3,66E-05
HNRNPA0	-0,6105546	3,68E-05
GNPDA2	0,90763952	3,71E-05
RFC4	-0,6965973	3,75E-05
PLCD4	0,61251339	3,76E-05
LRRC1	-0,5945832	3,78E-05
TTC28	-0,6275287	3,81E-05
HMGN2P17	-0,6075779	3,85E-05
NSUN3	0,61977467	3,90E-05
PFDN4	0,61610605	3,99E-05
DDIT3	0,73375959	4,03E-05
ARHGAP18	-0,6158377	4,03E-05
C10orf114	-0,6434966	4,06E-05
HOXA13	-0,7964856	4,08E-05
FLJ44635	-0,664078	4,09E-05
CRBN	0,65829583	4,10E-05
CDC7	-0,6078474	4,10E-05
RBPMS2	0,5996973	4,14E-05
ZKSCAN3	0,64874712	4,14E-05
FZD4	-0,5965957	4,17E-05
HIST1H4J	-0,6347609	4,26E-05
TBC1D4	-0,7174497	4,38E-05
LINC00327	-0,7812364	4,44E-05
TRIQK	-0,6378636	4,45E-05
ZNF10	0,84343733	4,55E-05
ITGB3	0,59441547	4,55E-05
SUPV3L1	0,62984065	4,56E-05
MMP12	0,83766627	4,61E-05
WWP1	0,65349126	4,62E-05
RBBP8	-0,7255994	4,63E-05
LGR5	-0,8125017	4,66E-05
HSF2	0,73918451	4,67E-05
MAP2K6	-0,5891933	4,69E-05
MIS18BP1	-0,6266633	4,71E-05
ZNF436	-0,6239773	4,71E-05
PPP4R4	0,67335389	4,74E-05
INHBB	-0,6005351	4,75E-05
LINC00707	-0,698375	4,76E-05
FANCB	-1,0033849	4,79E-05
FBXO30	0,70531025	4,82E-05
SLC38A6	0,72404426	4,84E-05
CHML	-1,1338897	4,91E-05
MIR612	0,69861728	4,99E-05
MBNL3	-0,6579245	4,99E-05
TRANK1	-0,7216277	5,05E-05
S1PR3	-0,606676	5,05E-05
SPRED1	-0,6852446	5,10E-05
HSPB3	0,92687471	5,11E-05
KIAA0922	-0,7768663	5,13E-05
PLSCR1	-0,7225571	5,15E-05
ALDH7A1	-0,7159776	5,15E-05
DOK6	-0,6421177	5,19E-05
FZD1	-0,9034232	5,26E-05
APAF1	-0,7575928	5,28E-05
DPY19L1	-0,5865462	5,47E-05
TMOD2	-0,6169524	5,51E-05
ZSCAN26	0,6193625	5,58E-05
COPG2	-0,6295054	5,63E-05
L3HYPDH	0,60977793	5,75E-05
HIST1H1C	-0,7284203	6,02E-05
LINC00630	0,72372378	6,14E-05
POLQ	-0,8872528	6,19E-05
LMOD1	-0,6286786	6,48E-05
ZNF33B	-0,5938062	6,48E-05
LIFR	-0,627515	6,49E-05
HSPA7	0,8086028	6,53E-05
ABHD10	-0,7685879	6,58E-05
HIBCH	0,70603969	6,61E-05
HSDL1	0,6338907	6,67E-05
KNSTRN	-0,7155192	6,69E-05
POLD3	-0,6018976	6,77E-05
TMEM194A	-0,6376624	6,83E-05
ACVR2A	-0,67449	6,85E-05
TSHZ2	-0,6370849	6,92E-05
KRBOX4	0,62009858	7,03E-05
POLR3A	0,6067129	7,08E-05
SNORA38B	-0,6988079	7,15E-05
AP4B1	0,71295979	7,16E-05
GBP5	-0,6758803	7,17E-05
XRCC2	-0,8116097	7,20E-05
LYST	0,61117846	7,27E-05
LOC1003792	0,75297793	7,38E-05
VPS29	0,65794389	7,38E-05

OXCT1	-0,655614	1,62E-05
MID2	0,75552843	1,66E-05
DDIT3	0,76273092	1,66E-05
CCNC	0,59568662	1,66E-05
MMS22L	-0,9266595	1,66E-05
FAM134B	0,66353509	1,67E-05
SHOX	-0,674468	1,67E-05
FTSJ3	0,61546977	1,67E-05
HIST1H3C	-0,9311449	1,67E-05
FGF7	-0,743537	1,70E-05
CENPW	-0,6315898	1,73E-05
TCEB3	0,63575688	1,73E-05
F2RL2	0,65058084	1,73E-05
LOC730101	-0,7049019	1,74E-05
HIST1H2AL	-0,6715861	1,74E-05
MIR3198-1	-0,9519143	1,74E-05
OR1J4	0,778756	1,75E-05
UTRN	-0,6444454	1,76E-05
SPR	0,71814261	1,77E-05
KRT33A	-0,629708	1,79E-05
TP53I13	0,61927624	1,79E-05
NEK1	0,74173669	1,79E-05
CISD1	0,67994951	1,79E-05
GYS1	0,58530964	1,80E-05
BBS5	0,71976344	1,80E-05
QRSL1	0,7981596	1,80E-05
DGKI	-0,6268823	1,83E-05
RTKN2	-0,7734558	1,83E-05
POLA1	-0,916447	1,86E-05
DIAPH2	-0,6413425	1,87E-05
HIST1H2BH	-0,7055259	1,90E-05
RBBP8	-0,6776146	1,91E-05
SYNE1	-0,680258	1,91E-05
DISP1	0,66136236	1,93E-05
P2RX6	0,60472125	1,93E-05
LOC100130992	-0,8319821	1,94E-05
SAMHD1	-0,6342323	1,96E-05
CAMKK1	0,7383269	1,96E-05
LINC00630	0,7805863	1,99E-05
CEP290	0,93664949	2,03E-05
CYP7B1	-0,7898631	2,04E-05
ZFP36L2	-0,6940269	2,10E-05
IL36B	1,12007932	2,12E-05
FZD4	-0,6191131	2,12E-05
L3HYPDH	0,64352825	2,12E-05
PCDH12	0,71990494	2,13E-05
DGKH	-0,6687775	2,15E-05
TENM4	-0,5864095	2,15E-05
SESN3	-0,7462084	2,16E-05
ZSCAN26	0,69820948	2,19E-05
CENPT	0,79171697	2,19E-05
LRPPRC	0,60547027	2,20E-05
HIST1H4E	-0,7093025	2,20E-05
SCRT2	0,8863729	2,20E-05
CCDC109B	-0,6262936	2,26E-05
CCDC71L	-0,6760472	2,27E-05
LMOD1	-0,631697	2,29E-05
RBMS2	-0,5894176	2,29E-05
XYLT1	-0,5979921	2,30E-05
CYP2R1	0,59348754	2,31E-05
TET1	-0,6281224	2,31E-05
ASF1B	-0,7056787	2,33E-05
TXNDC17	-0,5909589	2,34E-05
COA6	0,68666763	2,35E-05
EMG1	0,60152838	2,39E-05
CDH8	-0,6647526	2,41E-05
ZC2HC1A	-0,6173472	2,42E-05
TBC1D4	-0,7358022	2,49E-05
CARD6	0,6151612	2,53E-05
KIAA1841	0,63441891	2,54E-05
ERI3	0,61466904	2,55E-05
SLC48A1	0,59301618	2,56E-05
MAB21L1	-0,7927741	2,57E-05
SNORD89	0,58554325	2,58E-05
TTC28	-0,6113911	2,59E-05
RNA5SP108	0,81843381	2,60E-05
SIRT1	-0,5920155	2,60E-05
TRANK1	-0,6793606	2,61E-05
AS3MT	0,76517773	2,66E-05
ALDH7A1	-0,6328947	2,66E-05
PDE12	0,66302106	2,66E-05
RAD51	-0,6095984	2,68E-05
ZNF521	-0,5948614	2,68E-05
FKBP7	-0,5962588	2,72E-05
TSHZ2	-0,5996811	2,75E-05

FASTKD2	0,84088138	7,43E-05
MAB21L1	-0,7490918	7,44E-05
OTTHUMG0	0,92802509	7,53E-05
CTDSPL2	-0,6484875	7,59E-05
ZFP36	0,5856079	7,64E-05
ARNTL2	-0,6481725	7,77E-05
TLR3	-0,6067847	7,90E-05
SNRNP48	0,72001749	8,03E-05
E2F7	-0,6761983	8,19E-05
DEPDC7	-0,7074176	8,35E-05
ZC3H6	-0,5935025	8,45E-05
OTTHUMG0	-0,6177613	8,48E-05
CYB5R4	0,66642487	8,52E-05
EXOC8	0,86673637	8,52E-05
CENPH	-0,6594406	8,52E-05
LOC90784	0,63201739	8,58E-05
HMGN1P30	-0,6206846	8,62E-05
GABARAPL3	0,63825463	8,70E-05
LRRC37B	0,71460386	8,83E-05
ZC2HC1A	-0,6434179	8,85E-05
MIR214	-0,6101908	8,87E-05
TMEM62	0,72963735	8,99E-05
CEP128	-0,6047541	9,32E-05
DMXL1	0,61097541	9,41E-05
ATP8B4	-0,8931918	9,44E-05
FJX1	-0,6200189	9,50E-05
ST13P4	0,76068333	9,73E-05
ATAD5	-0,7201866	9,73E-05
SAT1	0,64992659	0,00010261
DBC1	-0,6133861	0,000105
PRSS12	-0,5984877	0,00010797
XCR1	0,67200783	0,00011364
HIST2H3A	-0,7636259	0,00011438
LINC00669	-0,5955846	0,00011461
KRTAP2-3	-0,6880749	0,00011524
PSTPIP2	-0,6323201	0,00011564
HSP90AA4P	1,45362563	0,00011896
FAM63B	-0,5866099	0,0001199
MOSPD2	-0,6157034	0,00012162
SEH1L	-0,6197188	0,00012339
AS3MT	0,64050078	0,00012339
DPY19L2	-0,7025708	0,00012713
OTTHUMG0	0,82552075	0,00012793
SIRT1	-0,6150681	0,00012971
TTPAL	-0,6332017	0,00012973
FOPNL	0,65900706	0,00013131
QRSL1	0,65681734	0,0001321
HIST1H3A	-0,7347856	0,00013229
DDX60	-0,7496108	0,00013516
GPAM	-0,6178304	0,00013696
PRKCI	-0,5888038	0,00014233
ZNF222	0,6799966	0,00014675
GREB1L	-0,6565649	0,00014802
TLR6	0,78791928	0,00014814
TRIM37	0,61087444	0,00014893
ELP4	-0,6424962	0,00014972
TRAPPC2	0,61219768	0,00015199
IL36B	1,12964376	0,00015697
SLC12A2	-0,6108172	0,00015783
CHAF1B	-0,673716	0,00015895
LOC1001309	-0,8542408	0,00015895
OTTHUMG0	-0,7266433	0,00015946
ZNF234	0,58648641	0,00016074
BBS5	0,62872349	0,00016087
ACSS3	-0,64523	0,00016239
PM20D2	-0,8007686	0,0001634
CCP110	-0,6250468	0,00016966
HIST1H2BL	-0,6121077	0,00017242
NARS2	-0,6528865	0,00017273
TP53INP1	-0,6679684	0,00017425
GABRE	0,67213579	0,00017661
YEATS4	-0,6314359	0,00017661
DPY19L2P1	-0,6242356	0,00017833
TBC1D8B	-0,6829502	0,00017886
ZNF175	0,64635259	0,00018005
TXNDC17	-0,705252	0,00018322
NHLRC2	-0,6299152	0,00018327
HIST1H2AI	-0,5856473	0,00018371
ERO1LB	0,60573875	0,00019568
DCLRE1A	-0,6442958	0,00020095
PCDHB12	-0,6971306	0,00020095
TMEM194B	-0,6098754	0,00020237
MTBP	-0,7616962	0,00020268
GPR37	-0,586557	0,00020866
SCN1A	-0,6483746	0,00021377

HIST1H2BI	-1,2667661	2,75E-05
PRRX2	-0,5916301	2,75E-05
ZBTB43	0,5987296	2,76E-05
SMIM10	0,73426623	2,77E-05
KDM2A	0,60620745	2,78E-05
PRKD1	-0,5867673	2,79E-05
ZNF770	0,59838552	2,81E-05
CHML	-1,0431318	2,85E-05
CTSC	-0,6462512	2,90E-05
CASP6	0,63346967	2,92E-05
LINC00707	-0,8160258	2,92E-05
PGPEP1	0,72608199	2,94E-05
POLR3A	0,63715317	2,97E-05
ZRANB2	0,62057059	2,98E-05
ZNF175	0,77580404	3,02E-05
OTTHUMG00000	1,23534455	3,03E-05
HMGN2P11	-0,660138	3,03E-05
FAM111A	-0,6997107	3,04E-05
ADH1C	-0,6181117	3,05E-05
PGM2	-0,6751951	3,06E-05
MGAT5	0,58628028	3,06E-05
GABRE	0,6392426	3,06E-05
HERC2P9	0,60387471	3,14E-05
ITGB3	0,58524933	3,15E-05
DOCK2	-0,641467	3,16E-05
FGF9	-0,6051368	3,17E-05
SNRNP48	0,7584704	3,18E-05
TRIM37	0,66090566	3,24E-05
HIST1H2BO	-0,7367945	3,28E-05
TTC5	0,64109648	3,30E-05
HERC2P2	0,59448224	3,33E-05
STRA13	0,97610731	3,34E-05
FAM101B	-0,6385232	3,36E-05
CLCN3	0,58952544	3,38E-05
ZNF483	0,71523643	3,40E-05
PRCC	0,61306546	3,42E-05
SOCS5	-0,6431385	3,44E-05
PTX3	-0,6342735	3,47E-05
MOCOS	0,64339372	3,47E-05
CCL26	1,06480055	3,47E-05
AFF2	-0,6848542	3,49E-05
CYB561D2	0,6233816	3,50E-05
CARHSP1	-0,6115955	3,59E-05
DHX16	0,62877976	3,60E-05
HIST1H3G	-0,8869018	3,61E-05
POLQ	-0,8897792	3,62E-05
LGR5	-0,734917	3,63E-05
NAPB	0,85484457	3,64E-05
OTTHUMG00000	-0,8804259	3,68E-05
LOC100506136	0,65703539	3,68E-05
MBNL3	-0,6179196	3,68E-05
NOG	-0,8371362	3,69E-05
MRPL46	0,62199055	3,73E-05
CCP110	-0,6419863	3,74E-05
SUPT7L	0,60113047	3,76E-05
MIR21	-0,9721152	3,82E-05
C12orf4	0,60727593	3,84E-05
HOXD-AS1	0,59397249	3,88E-05
LRRC6	0,63740316	3,94E-05
MTX1	0,64793588	3,99E-05
ADRA2A	-0,7921576	4,08E-05
LINC00327	-0,7624954	4,08E-05
SYT2	0,71043886	4,12E-05
PPAPDC1B	0,59552003	4,12E-05
TRUB2	0,60211846	4,13E-05
TICRR	-0,8158982	4,16E-05
LOC100379224	0,82367126	4,22E-05
RAD54B	-0,6205166	4,24E-05
CSRNP3	0,68744475	4,25E-05
HIST1H1E	-0,6546889	4,25E-05
FANCB	-0,9363283	4,44E-05
C4orf46	-0,6818683	4,46E-05
ZNF222	0,66547964	4,46E-05
TMEM194A	-0,5872453	4,50E-05
RUVBL2	0,60234748	4,57E-05
GREB1L	-0,6490505	4,68E-05
HOXA13	-0,7259227	4,68E-05
HIST1H4B	-0,7988278	4,68E-05
GABBR2	-0,6083575	4,70E-05
VPS29	0,70543269	4,75E-05
FBXO30	0,70755861	4,83E-05
ALG10	-0,7143953	4,98E-05
DMXL1	0,64083046	5,05E-05
FAM32A	0,61171468	5,11E-05
FLJ44635	-0,6794373	5,12E-05

OTTHUMG0	0,62993378	0,00022361
ZBTB8A	-0,5986382	0,00023338
ZNF484	0,68328281	0,000235
OIP5	-0,821114	0,00024203
DOCK11	-0,6147224	0,00024311
HSP90AA6P	0,76293184	0,00024633
ALS2	0,5946818	0,00025351
HMGN1P38	-0,630359	0,00026447
ZNF483	0,60372837	0,00026897
MIR3192	0,65455169	0,00029381
BLID	-0,6628101	0,00029657
ABCA9	-0,6323451	0,00031483
SNORD113-	-1,0334539	0,0003153
OTTHUMG0	0,69987375	0,00031561
DSN1	-0,6545355	0,00032633
AQP9	-0,7785824	0,00032915
GAN	0,65084991	0,00033974
SYT2	0,61377908	0,00034697
CENPQ	-0,8353566	0,00037682
STIL	-0,6326331	0,00037859
GK3P	0,72682988	0,00040692
OTTHUMG0	-0,6014392	0,0004138
FAM87B	0,60464824	0,00041793
SLC25A40	-0,607399	0,00042112
PDE12	0,59199246	0,00042336
PRIM1	-0,8773455	0,00044185
MIR21	-0,6464159	0,00048102
MGC27345	0,66794262	0,00053728
PGM2L1	-0,6075437	0,00054439
HIST1H2AK	-0,6221237	0,00056819
HAUS1	-0,6055151	0,00058553
LOC727896	0,73795049	0,00062094
SNORD113-	-0,6402084	0,00066347
OTTHUMG0	0,78341748	0,00067175
MB21D2	-0,5920505	0,00069873
MIS18A	-0,6450412	0,00075704
OTTHUMG0	0,63860206	0,00076611
HIST1H2BB	-0,6718781	0,00089176
OR1J4	0,62519321	0,0009105
RNA5SP108	0,81390315	0,00092384
LOC1001295	-0,7467186	0,00092546
TXNRD3NB	-0,5853807	0,00094152
PCDHB9	-0,6406223	0,00095622
LMO7-AS1	-0,7043442	0,00103895
KCNJ2-AS1	-0,6513557	0,00125225
HIST1H3H	-0,8495051	0,00141117
RNU5E-4P	0,60469004	0,00141742
LOC646543	0,71910116	0,00172
HIST1H4F	-0,6795273	0,00184893
OTTHUMG0	-0,606804	0,00187433
OR1Q1	0,59660928	0,00203119
SNORD113-	-1,0604399	0,00205197
MIR553	0,95224718	0,00247868
RNU6-51	-0,7519288	0,00257395
MIR1244-1	-0,7389778	0,00267975
HIST1H1D	-0,7198625	0,00271781
SNORD114-	-0,5903436	0,00373973
OTTHUMG0	-0,8477088	0,0046046
MIR590	0,67880439	0,0050366
MIR1245A	-0,6321068	0,00523619
MIR616	0,6326975	0,00616277
RNA5SP385	-0,5923005	0,00635585
RNU5D-1	0,72879089	0,00863632
IGKV2-40	-0,6585736	0,00979033
OTTHUMG0	0,67039626	0,01142961
FABP3P2	-0,5883905	0,02194603
OTTHUMG0	-0,5985953	0,02450769

LRRC37B	0,67469023	5,15E-05
LOC100507254	-0,6580248	5,41E-05
RFC3	-0,621112	5,46E-05
SPIN4	-1,0235997	5,48E-05
OTTHUMG00000	0,97790363	5,52E-05
GBP5	-0,6082437	5,94E-05
ZBTB8A	-0,6229643	6,13E-05
GSTCD	-0,6160954	6,23E-05
PM20D2	-0,7010293	6,28E-05
XCR1	0,59785424	6,29E-05
YOD1	0,58973761	6,33E-05
DCTN3	0,6193139	6,57E-05
HIST1H4J	-0,5967896	6,59E-05
KRTAP2-3	-0,651654	6,73E-05
CEP128	-0,6145634	6,76E-05
OTTHUMG00000	0,74362712	6,88E-05
HIST1H1C	-0,6343599	7,08E-05
HIST1H1B	-1,1503212	7,11E-05
IRAK3	0,61730617	7,14E-05
TBC1D8B	-0,634045	7,18E-05
PLSCR1	-0,6026081	7,26E-05
CHAF1B	-0,6152723	7,45E-05
RNASEH2A	-0,6400929	7,52E-05
RPS6KA6	0,6085733	7,53E-05
FAM63B	-0,5971356	7,64E-05
LOC90784	0,70896368	7,67E-05
GK3P	0,83675409	7,70E-05
ZNF234	0,64264437	7,80E-05
MXRA5P1	-0,6155117	7,81E-05
ZNF189	0,58739108	7,91E-05
SPC24	-0,669711	8,26E-05
SCN1A	-0,6353843	8,54E-05
AQP9	-0,8022521	8,63E-05
TP53INP1	-0,6054882	8,71E-05
CENPH	-0,6383535	8,75E-05
HMGN2P17	-0,5928658	8,82E-05
MIR214	-0,6241385	8,84E-05
PLA2G4A	0,67255879	9,21E-05
PFDN4	0,63782887	9,31E-05
MBOAT4	0,88931016	9,40E-05
RBM48	0,63015982	9,40E-05
LIPH	0,64611106	9,63E-05
HIST1H2BM	-0,8983743	9,87E-05
LOC643723	0,61423882	9,91E-05
GINS2	-0,6570509	0,00010049
TMEM115	0,59668038	0,00010136
ULBP3	0,64587322	0,00010266
EMX2	-0,6453511	0,00010349
LYST	0,58967783	0,0001055
OTTHUMG00000	0,587888	0,0001136
WDR83OS	0,60606595	0,00011569
ALS2	0,62567123	0,00012087
SMG8	0,63500252	0,00012161
MIRLET7I	-0,5947809	0,00012502
NHLRC2	-0,6058105	0,00012559
C18orf25	0,58966665	0,00013456
MTBP	-0,7199987	0,00013469
FAM72C	-0,8455555	0,00013971
PCDHB12	-0,7126443	0,00014218
ABCA5	0,6013723	0,00015116
MIR3192	0,68775626	0,00015464
MOAP1	0,59664968	0,00016462
GPR37	-0,6580128	0,00016565
NHLRC1	0,88467097	0,00018988
PGM2L1	-0,6366843	0,00019854
FPR1	0,66973478	0,00019929
MGC27345	0,69710424	0,00019974
OTTHUMG00000	0,73069131	0,01866153
LRRC66	-0,5988877	0,02325945
MIR590	0,61054778	0,02771792
OTTHUMG00000	-0,5926783	0,03043932

Table S3. Impact of proteostasis regulators on global gene expression. Differential gene expression was defined by >1.5-fold difference with a p-value threshold of 0.05.

Treatment / Reg. genes	DGJ	MG132	BTZ	CLC	EerI	ABX	DGJ +MG132	DGJ +EerI	DGJ +ABX
**Upregulated genes**	0	608	540	247	218	1	731	246	1
**Downregulated genes**	0	724	520	224	294	1	668	349	9
**Sum of regulated genes**	0	1332	1060	471	512	2	1399	595	10

Table S4. Overrepresented signaling pathways within the transcriptional signature of MG132. Shown are the significantly overrepresented pathways with the 12 lowest corresponding p-values.

No.	WIKI pathway
1	Amino_acid_conjugation_WP715
2	Glucose_Homeostasis_WP661
3	HIF1A_and_PPARG_regulation_of_glycolysis_WP2456
4	Phospholipid_biosynthesis_WP2457
**5**	**Proteasome_Degradation_WP183**
6	Retinoblastoma_(RB)_in_Cancer_WP2446
**7**	**Parkin-Ubiquitin_Proteasomal_System_pathway_WP2359**
8	Cell_Cycle_WP179
9	Histone_Modifications_WP2369
10	DNA_Replication_WP466
11	Gastric_Cancer_Network_1_WP2361
12	G1_to_S_cell_cycle_control_WP45

bold: proteostasis-associated pathways

Table S5. Overrepresented signaling pathways within the transcriptional signature of BTZ. Shown are the significantly overrepresented pathways with the 12 lowest corresponding p-values.

No.	WIKI pathway
1	Amino_acid_conjugation_WP715
2	Glucose_Homeostasis_WP661
3	HIF1A_and_PPARG_regulation_of_glycolysis_WP2456
4	Phospholipid_biosynthesis_WP2457
5	**Proteasome_Degradation_WP183**
6	Retinoblastoma_(RB)_in_Cancer_WP2446
7	**Parkin-Ubiquitin_Proteasomal_System_pathway_WP2359**
8	Cell_Cycle_WP179
9	Integrated_Pancreatic_Cancer_Pathway_WP2377
10	G1_to_S_cell_cycle_control_WP45
11	Histone_Modifications_WP2369
12	Gastric_Cancer_Network_1_WP2361

bold: proteostasis-associated pathways

Table S6. Overrepresented signaling pathways within the transcriptional signature of CLC. Shown are the significantly overrepresented pathways with the 12 lowest corresponding p-values.

No.	WIKI pathway
1	Amino_acid_conjugation_WP715
2	Glucose_Homeostasis_WP661
3	HIF1A_and_PPARG_regulation_of_glycolysis_WP2456
4	Histone_Modifications_WP2369
5	Phospholipid_biosynthesis_WP2457
**6**	**Proteasome_Degradation_WP183**
**7**	**Parkin-Ubiquitin_Proteasomal_System_pathway_WP2359**
8	Retinoblastoma_(RB)_in_Cancer_WP2446
9	Gastric_Cancer_Network_1_WP2361
10	Copper_homeostasis_WP3286
11	Polyol_Pathway_WP690
12	Zinc_homeostasis_WP3529

bold: proteostasis-associated pathways

Table S7. Overrepresented signaling pathways within the transcriptional signature of EerI. Shown are the significantly overrepresented pathways with the 12 lowest corresponding p-values.

No.	WIKI pathway
1	Amino_acid_conjugation_WP715
2	Cell_Cycle_WP179
3	Glucose_Homeostasis_WP661
4	HIF1A_and_PPARG_regulation_of_glycolysis_WP2456
5	Phospholipid_biosynthesis_WP2457
**6**	**Proteasome_Degradation_WP183**
7	Retinoblastoma_(RB)_in_Cancer_WP2446
8	Histone_Modifications_WP2369
9	DNA_Replication_WP466
10	G1_to_S_cell_cycle_control_WP45
11	Parkin-Ubiquitin_Proteasomal_System_pathway_WP2359
**12**	**NRF2_pathway_WP2884**

bold: proteostasis-associated pathways

Table S8. List of all classified proteostasis genes

Protein name (UniProt)	Gene symbol (HGNC)	HGNC-ID
Very long-chain specific acyl-CoA dehydrogenase, mitochondrial	ACADVL	HGNC:92
Alpha-adducin	ADD1	HGNC:243
Proteasomal ubiquitin receptor ADRM1	ADRM1	HGNC:15759
Activator of 90 kDa heat shock protein ATPase homolog 1	AHSA1	HGNC:1189
Alpha-1,3/1,6-mannosyltransferase ALG2	ALG2	HGNC:23159
E3 ubiquitin-protein ligase AMFR	AMFR	HGNC:463
Ankyrin repeat and SAM domain-containing protein 4B	ANKS4B	HGNC:26795
ADP-ribosylation factor GTPase-activating protein 1	ARFGAP1	HGNC:15852
ATPase ASNA1	ASNA1	HGNC:752
Asparagine synthetase [glutamine-hydrolyzing]	ASNS	HGNC:753
Cyclic AMP-dependent transcription factor ATF-3	ATF3	HGNC:785
Cyclic AMP-dependent transcription factor ATF-4	ATF4	HGNC:786
Cyclic AMP-dependent transcription factor ATF-6 alpha	ATF6	HGNC:791
Cyclic AMP-dependent transcription factor ATF-6 beta	ATF6B	HGNC:2349
V-type proton ATPase subunit d 1	ATP6V0D1	HGNC:13724
Ataxin-3	ATXN3	HGNC:7106
Ancient ubiquitous protein 1	AUP1	HGNC:891
Large proline-rich protein BAG6	BAG6	HGNC:13919
B-cell receptor-associated protein 31	BCAP31	HGNC:16695
Class A basic helix-loop-helix protein 15	BHLHA15	HGNC:22265
Baculoviral IAP repeat-containing protein 6	BIRC6	HGNC:13516
F-box/WD repeat-containing protein 1A	BTRC	HGNC:1144
Calreticulin	CALR	HGNC:1455
Calnexin	CANX	HGNC:1473
C-C motif chemokine 2	CCL2	HGNC:10618
G1/S-specific cyclin-D1	CCND1	HGNC:1582
T-complex protein 1 subunit delta	CCT4	HGNC:1617
T-complex protein 1 subunit eta	CCT7	HGNC:1622
Ubiquitin-conjugating enzyme E2 R1	CDC34	HGNC:1734
Hsp90 co-chaperone Cdc37	CDC37	HGNC:1735
Probable serine carboxypeptidase CPVL	CPVL	HGNC:14399
Cyclic AMP-responsive element-binding protein 3	CREB3	HGNC:2347
Cyclic AMP-responsive element-binding protein 3-like protein 1	CREB3L1	HGNC:18856
Cyclic AMP-responsive element-binding protein 3-like protein 2	CREB3L2	HGNC:23720
Cyclic AMP-responsive element-binding protein 3-like protein 3	CREB3L3	HGNC:18855
Cyclic AMP-responsive element-binding protein 3-like	CREB3L4	HGNC:18854

protein 4		
CREB3 regulatory factor	CREBRF	HGNC:24050
Alpha-crystallin B chain	CRYAB	HGNC:2389
Carboxy-terminal domain RNA polymerase II polypeptide A small phosphatase 2	CTDSP2	HGNC:17077
Cullin-7	CUL7	HGNC:21024
Interleukin-8	CXCL8	HGNC:6025
CXXC-type zinc finger protein 1	CXXC1	HGNC:24343
Disabled homolog 2-interacting protein	DAB2IP	HGNC:17294
Dynactin subunit 1	DCTN1	HGNC:2711
DNA damage-inducible transcript 3 protein	DDIT3	HGNC:2726
Dolichyl-diphosphooligosaccharide--protein glycosyltransferase 48 kDa subunit	DDOST	HGNC:2728
DDRGK domain-containing protein 1	DDRGK1	HGNC:16110
ATP-dependent DNA helicase DDX11	DDX11	HGNC:2736
Derlin-1	DERL1	HGNC:28454
Derlin-2	DERL2	HGNC:17943
Derlin-3	DERL3	HGNC:14236
DnaJ homolog subfamily A member 1	DNAJA1	HGNC:5229
DnaJ homolog subfamily B member 1	DNAJB1	HGNC:5270
DnaJ homolog subfamily B member 11	DNAJB11	HGNC:14889
DnaJ homolog subfamily B member 12	DNAJB12	HGNC:14891
DnaJ homolog subfamily B member 2	DNAJB2	HGNC:5228
DnaJ homolog subfamily B member 9	DNAJB9	HGNC:6968
DnaJ homolog subfamily C member 1	DNAJC1	HGNC:20090
DnaJ homolog subfamily C member 10	DNAJC10	HGNC:24637
DnaJ homolog subfamily C member 3	DNAJC3	HGNC:9439
DnaJ homolog subfamily C member 5	DNAJC5	HGNC:16235
ER degradation-enhancing alpha-mannosidase-like protein 1	EDEM1	HGNC:18967
ER degradation-enhancing alpha-mannosidase-like protein 2	EDEM2	HGNC:15877
ER degradation-enhancing alpha-mannosidase-like protein 3	EDEM3	HGNC:16787
Eukaryotic translation initiation factor 2A	EIF2A	HGNC:3254
Eukaryotic translation initiation factor 2-alpha kinase 1	EIF2AK1	HGNC:24921
Interferon-induced, double-stranded RNA-activated protein kinase	EIF2AK2	HGNC:9437
Eukaryotic translation initiation factor 2-alpha kinase 3	EIF2AK3	HGNC:3255
eIF-2-alpha kinase GCN2	EIF2AK4	HGNC:19687
Translation initiation factor eIF-2B subunit alpha	EIF2B1	HGNC:3257
Translation initiation factor eIF-2B subunit beta	EIF2B2	HGNC:3258
Translation initiation factor eIF-2B subunit delta	EIF2B4	HGNC:3260
Translation initiation factor eIF-2B subunit epsilon	EIF2B5	HGNC:3261
Eukaryotic translation initiation factor 2 subunit 1	EIF2S1	HGNC:3265

ER membrane protein complex subunit 1	EMC1	HGNC:28957
ER membrane protein complex subunit 10	EMC10	HGNC:27609
ER membrane protein complex subunit 2	EMC2	HGNC:28963
ER membrane protein complex subunit 3	EMC3	HGNC:23999
ER membrane protein complex subunit 4	EMC4	HGNC:28032
ER membrane protein complex subunit 6	EMC6	HGNC:28430
ER membrane protein complex subunit 7	EMC7	HGNC:24301
ER membrane protein complex subunit 8	EMC8	HGNC:7864
ER membrane protein complex subunit 9	EMC9	HGNC:20273
Endoplasmic reticulum lectin 1	ERLEC1	HGNC:25222
Erlin-1	ERLIN1	HGNC:16947
Erlin-2	ERLIN2	HGNC:1356
Serine/threonine-protein kinase/endoribonuclease IRE1	ERN1	HGNC:3449
Serine/threonine-protein kinase/endoribonuclease IRE2	ERN2	HGNC:16942
ERO1-like protein alpha	ERO1L	HGNC:13280
ERO1-like protein beta	ERO1LB	HGNC:14355
Endoplasmic reticulum resident protein 27	ERP27	HGNC:26495
Endoplasmic reticulum resident protein 29	ERP29	HGNC:13799
Endoplasmic reticulum resident protein 44	ERP44	HGNC:18311
Extended synaptotagmin-1	ESYT1	HGNC:29534
Exostosin-like 3	EXTL3	HGNC:3518
FAS-associated factor 2	FAF2	HGNC:24666
Protein Niban	FAM129A	HGNC:16784
Protein FAM8A1	FAM8A1	HGNC:16372
F-box only protein 2	FBXO2	HGNC:13581
F-box only protein 6	FBXO6	HGNC:13585
F-box/WD repeat-containing protein 11	FBXW11	HGNC:13607
Peptidyl-prolyl cis-trans isomerase FKBP10	FKBP10	HGNC:18169
Peptidyl-prolyl cis-trans isomerase FKBP14	FKBP14	HGNC:18625
Peptidyl-prolyl cis-trans isomerase FKBP2	FKBP2	HGNC:3718
Peptidyl-prolyl cis-trans isomerase FKBP5	FKBP5	HGNC:3721
Peptidyl-prolyl cis-trans isomerase FKBP7	FKBP7	HGNC:3723
FAD-dependent oxidoreductase domain-containing protein 2	FOXRED2	HGNC:26264
Neutral alpha-glucosidase AB	GANAB	HGNC:4138
Golgi to ER traffic protein 4 homolog	GET4	HGNC:21690
Glutamine--fructose-6-phosphate aminotransferase [isomerizing] 1	GFPT1	HGNC:4241
Receptor of activated protein C kinase 1 (RACK1)	GNB2L1	HGNC:4399
Golgin subfamily B member 1	GOLGB1	HGNC:4429
Golgi SNAP receptor complex member 2	GOSR2	HGNC:4431
Glycogen synthase kinase-3 alpha	GSK3A	HGNC:4616
E3 ubiquitin-protein ligase HACE1	HACE1	HGNC:21033

Hepatoma-derived growth factor	HDGF	HGNC:4856
Homocysteine-responsive endoplasmic reticulum-resident ubiquitin-like domain member 1 protein	HERPUD1	HGNC:13744
Minor histocompatibility antigen H13	HM13	HGNC:16435
Heat shock protein HSP 90-alpha	HSP90AA1	HGNC:5253
Heat shock protein HSP 90-beta	HSP90AB1	HGNC:5258
Endoplasmin	HSP90B1	HGNC:12028
Heat shock 70 kDa protein 1A	HSPA1A	HGNC:5232
Heat shock 70 kDa protein 1B	HSPA1B	HGNC:5233
78 kDa glucose-regulated protein	HSPA5	HGNC:5238
Heat shock cognate 71 kDa protein	HSPA8	HGNC:5241
Heat shock protein beta-6	HSPB6	HGNC:26511
Hsp70-binding protein 1	HSPBP1	HGNC:24989
Heat shock protein 105 kDa	HSPH1	HGNC:16969
Hypoxia up-regulated protein 1	HYOU1	HGNC:16931
Interferon gamma	IFNG	HGNC:5438
Insulin-like growth factor-binding protein 1	IGFBP1	HGNC:5469
Insulin-induced gene 1 protein	INSIG1	HGNC:6083
Insulin-induced gene 2 protein	INSIG2	HGNC:20452
E3 ubiquitin-protein ligase Itchy homolog	ITCH	HGNC:13890
Inositol 1,4,5-trisphosphate receptor type 1	ITPR1	HGNC:6180
JNK1/MAPK8-associated membrane protein	JKAMP	HGNC:20184
ER lumen protein-retaining receptor 3	KDELR3	HGNC:6306
Kelch domain-containing protein 3	KLHDC3	HGNC:20704
Protein ERGIC-53	LMAN1	HGNC:6631
Vesicular integral-membrane protein VIP36	LMAN2	HGNC:16986
VIP36-like protein	LMAN2L	HGNC:19263
Prelamin-A/C	LMNA	HGNC:6636
Lon protease homolog 2, peroxisomal	LONP2	HGNC:20598
Endoplasmic reticulum mannosyl-oligosaccharide 1,2-alpha-mannosidase	MAN1B1	HGNC:6823
Mesencephalic astrocyte-derived neurotrophic factor	MANF	HGNC:15461
E3 ubiquitin-protein ligase MARCH6	MARCH6	HGNC:30550
Membrane-bound transcription factor site-1 protease	MBTPS1	HGNC:15456
Membrane-bound transcription factor site-2 protease	MBTPS2	HGNC:15455
Malectin	MLEC	HGNC:28973
Membrane magnesium transporter 1	MMGT1	HGNC:28100
Mannosyl-oligosaccharide glucosidase	MOGS	HGNC:24862
Myeloid-derived growth factor	MYDGF	HGNC:16948
Marginal zone B- and B1-cell-specific protein	MZB1	HGNC:30125
Nuclear factor erythroid 2-related factor 2	NFE2L2	HGNC:7782
Peptide-N(4)-(N-acetyl-beta-glucosaminyl)asparagine	NGLY1	HGNC:17646

amidase		
Nuclear protein localization protein 4 homolog	NPLOC4	HGNC:18261
NSFL1 cofactor p47	NSFL1C	HGNC:15912
Protein OS-9	OS9	HGNC:16994
Oligosaccharyltransferase complex subunit OSTC	OSTC	HGNC:24448
Ubiquitin thioesterase OTUB1	OTUB1	HGNC:23077
Protein disulfide-isomerase	P4HB	HGNC:8548
E3 ubiquitin-protein ligase parkin (PRKN)	PARK2	HGNC:8607
Mono [ADP-ribose] polymerase PARP16	PARP16	HGNC:26040
Protein disulfide-isomerase A2	PDIA2	HGNC:14180
Protein disulfide-isomerase A3	PDIA3	HGNC:4606
Protein disulfide-isomerase A4	PDIA4	HGNC:30167
Protein disulfide-isomerase A5	PDIA5	HGNC:24811
Protein disulfide-isomerase A6	PDIA6	HGNC:30168
Protein disulfide-isomerase-like protein of the testis	PDILT	HGNC:27338
Prefoldin subunit 2	PFDN2	HGNC:8867
Cytosolic phospholipase A2 beta	PLA2G4B	HGNC:9036
Peptidyl-prolyl cis-trans isomerase A	PPIA	HGNC:9253
Peptidyl-prolyl cis-trans isomerase B	PPIB	HGNC:9255
Protein phosphatase 1 regulatory subunit 15A	PPP1R15A	HGNC:14375
Serine/threonine-protein phosphatase 2A 56 kDa regulatory subunit beta isoform	PPP2R5B	HGNC:9310
Prolactin regulatory element-binding protein	PREB	HGNC:9356
Glucosidase 2 subunit beta	PRKCSH	HGNC:9411
Proteasome subunit alpha type-1	PSMA1	HGNC:9530
Proteasome subunit alpha type-2	PSMA2	HGNC:9531
Proteasome subunit alpha type-3	PSMA3	HGNC:9532
Proteasome subunit alpha type-4	PSMA4	HGNC:9533
Proteasome subunit alpha type-5	PSMA5	HGNC:9534
Proteasome subunit alpha type-6	PSMA6	HGNC:9535
Proteasome subunit alpha type-7	PSMA7	HGNC:9536
Proteasome subunit alpha type-7-like	PSMA8	HGNC:22985
Proteasome subunit beta type-1	PSMB1	HGNC:9537
Proteasome subunit beta type-2	PSMB2	HGNC:9539
Proteasome subunit beta type-3	PSMB3	HGNC:9540
Proteasome subunit beta type-4	PSMB4	HGNC:9541
Proteasome subunit beta type-5	PSMB5	HGNC:9542
Proteasome subunit beta type-6	PSMB6	HGNC:9543
Proteasome subunit beta type-7	PSMB7	HGNC:9544
Proteasome subunit beta type-8	PSMB8	HGNC:9545
Proteasome subunit beta type-9	PSMB9	HGNC:9546
Proteasome subunit beta type-10	PSMB10	HGNC:9538

26S proteasome regulatory subunit 4	PSMC1	HGNC:9547
26S proteasome regulatory subunit 7	PSMC2	HGNC:9548
26S proteasome regulatory subunit 6A	PSMC3	HGNC:9549
26S proteasome regulatory subunit 6B	PSMC4	HGNC:9551
26S proteasome regulatory subunit 8	PSMC5	HGNC:9552
26S proteasome regulatory subunit 10B	PSMC6	HGNC:9553
26S proteasome non-ATPase regulatory subunit 1	PSMD1	HGNC:9554
26S proteasome non-ATPase regulatory subunit 2	PSMD2	HGNC:9559
26S proteasome non-ATPase regulatory subunit 3	PSMD3	HGNC:9560
26S proteasome non-ATPase regulatory subunit 4	PSMD4	HGNC:9561
26S proteasome non-ATPase regulatory subunit 5	PSMD5	HGNC:9563
26S proteasome non-ATPase regulatory subunit 6	PSMD6	HGNC:9564
26S proteasome non-ATPase regulatory subunit 7	PSMD7	HGNC:9565
26S proteasome non-ATPase regulatory subunit 8	PSMD8	HGNC:9566
26S proteasome non-ATPase regulatory subunit 9	PSMD9	HGNC:9567
26S proteasome non-ATPase regulatory subunit 10	PSMD10	HGNC:9555
26S proteasome non-ATPase regulatory subunit 11	PSMD11	HGNC:9556
26S proteasome non-ATPase regulatory subunit 12	PSMD12	HGNC:9557
26S proteasome non-ATPase regulatory subunit 13	PSMD13	HGNC:9558
26S proteasome non-ATPase regulatory subunit 14	PSMD14	HGNC:16889
Proteasome activator complex subunit 1	PSME1	HGNC:9568
Proteasome activator complex subunit 2	PSME2	HGNC:9569
Proteasome activator complex subunit 3	PSME3	HGNC:9570
Proteasome activator complex subunit 4	PSME4	HGNC:20635
Proteasome inhibitor PI31 subunit	PSMF1	HGNC:9571
UV excision repair protein RAD23 homolog A	RAD23A	HGNC:9812
UV excision repair protein RAD23 homolog B	RAD23B	HGNC:9813
Rhomboid-related protein 3	RHBDL3	HGNC:16502
E3 ubiquitin-protein ligase RNF103	RNF103	HGNC:12859
RING finger protein 121	RNF121	HGNC:21070
E3 ubiquitin-protein ligase RNF128	RNF128	HGNC:21153
E3 ubiquitin-protein ligase RNF138	RNF138	HGNC:17765
E3 ubiquitin-protein ligase RNF139	RNF139	HGNC:17023
E3 ubiquitin-protein ligase RNF170	RNF170	HGNC:25358
RING finger protein 175	RNF175	HGNC:27735
E3 ubiquitin-protein ligase RNF185	RNF185	HGNC:26783
E3 ubiquitin-protein ligase NRDP1	RNF41	HGNC:18401
E3 ubiquitin-protein ligase RNF5	RNF5	HGNC:10068
Dolichyl-diphosphooligosaccharide--protein glycosyltransferase subunit 1	RPN1	HGNC:10381
Dolichyl-diphosphooligosaccharide--protein glycosyltransferase subunit 2	RPN2	HGNC:10382

Ubiquitin-40S ribosomal protein S27a	RPS27A	HGNC:10417
Secretory carrier-associated membrane protein 5	SCAMP5	HGNC:30386
Sterol regulatory element-binding protein cleavage-activating protein	SCAP	HGNC:30634
Stromal cell-derived factor 2-like protein 1	SDF2L1	HGNC:10676
Signal peptidase complex catalytic subunit SEC11C	SEC11C	HGNC:23400
Protein SEC13 homolog	SEC13	HGNC:10697
Protein transport protein Sec23A	SEC23A	HGNC:10701
Protein transport protein Sec23B	SEC23B	HGNC:10702
Protein transport protein Sec24A	SEC24A	HGNC:10703
Protein transport protein Sec24B	SEC24B	HGNC:10704
Protein transport protein Sec24C	SEC24C	HGNC:10705
Protein transport protein Sec24D	SEC24D	HGNC:10706
Protein transport protein Sec31A	SEC31A	HGNC:17052
Protein transport protein Sec61 subunit alpha isoform 1	SEC61A1	HGNC:18276
Protein transport protein Sec61 subunit alpha isoform 2	SEC61A2	HGNC:17702
Protein transport protein Sec61 subunit beta	SEC61B	HGNC:16993
Protein transport protein Sec61 subunit gamma	SEC61G	HGNC:18277
Translocation protein SEC62	SEC62	HGNC:11846
Translocation protein SEC63 homolog	SEC63	HGNC:21082
Protein sel-1 homolog 1	SEL1L	HGNC:10717
Protein sel-1 homolog 2	SEL1L2	HGNC:15897
Stress-associated endoplasmic reticulum protein 1	SERP1	HGNC:10759
Serpin H1	SERPINH1	HGNC:1546
Small glutamine-rich tetratricopeptide repeat-containing protein alpha	SGTA	HGNC:10819
SHC-transforming protein 1	SHC1	HGNC:10840
Nucleotide exchange factor SIL1	SIL1	HGNC:24624
E3 ubiquitin-protein ligase SMURF1	SMURF1	HGNC:16807
Signal peptidase complex subunit 2	SPCS2	HGNC:28962
Signal peptidase complex subunit 3	SPCS3	HGNC:26212
Sterol regulatory element-binding protein 1	SREBF1	HGNC:11289
Sterol regulatory element-binding protein 2	SREBF2	HGNC:11290
Signal recognition particle 19 kDa protein	SRP19	HGNC:11300
Signal recognition particle 54 kDa protein	SRP54	HGNC:11301
Signal recognition particle subunit SRP68	SRP68	HGNC:11302
Signal recognition particle receptor subunit alpha	SRPR	HGNC:11307
Signal recognition particle receptor subunit beta	SRPRB	HGNC:24085
Translocon-associated protein subunit alpha	SSR1	HGNC:11323
Translocon-associated protein subunit beta	SSR2	HGNC:11324
Translocon-associated protein subunit gamma	SSR3	HGNC:11325
Translocon-associated protein subunit delta	SSR4	HGNC:11326

Stress-induced-phosphoprotein 1	STIP1	HGNC:11387
Dolichyl-diphosphooligosaccharide--protein glycosyltransferase subunit STT3A	STT3A	HGNC:6172
Dolichyl-diphosphooligosaccharide--protein glycosyltransferase subunit STT3B	STT3B	HGNC:30611
E3 ubiquitin-protein ligase CHIP	STUB1	HGNC:11427
Sulfotransferase 1A3	SULT1A3	HGNC:11455
Small VCP/p97-interacting protein	SVIP	HGNC:25238
E3 ubiquitin-protein ligase synoviolin	SYVN1	HGNC:20738
Putative deoxyribonuclease TATDN2	TATDN2	HGNC:28988
Transducin beta-like protein 2	TBL2	HGNC:11586
T-complex protein 1 subunit alpha	TCP1	HGNC:11655
Talin-1	TLN1	HGNC:11845
E3 ubiquitin-protein ligase TM129	TMEM129	HGNC:25137
Transmembrane and ubiquitin-like domain-containing protein 1	TMUB1	HGNC:21709
Thioredoxin-related transmembrane protein 1	TMX1	HGNC:15487
Protein disulfide-isomerase TMX3	TMX3	HGNC:24718
Thioredoxin-related transmembrane protein 4	TMX4	HGNC:25237
Torsin-1A	TOR1A	HGNC:3098
Tripeptidyl-peptidase 1	TPP1	HGNC:2073
E3 ubiquitin-protein ligase TRIM13	TRIM13	HGNC:9976
Tripartite motif-containing protein 3	TRIM3	HGNC:10064
Testis-specific Y-encoded-like protein 2	TSPYL2	HGNC:24358
Thioredoxin domain-containing protein 11	TXNDC11	HGNC:28030
Thioredoxin domain-containing protein 16	TXNDC16	HGNC:19965
Ubiquitin-like modifier-activating enzyme 1	UBA1	HGNC:12469
Ubiquitin-like modifier-activating enzyme 5	UBA5	HGNC:23230
Ubiquitin-like modifier-activating enzyme 6	UBA6	HGNC:25581
Ubiquitin-associated domain-containing protein 2	UBAC2	HGNC:20486
Ubiquitin-conjugating enzyme E2 A	UBE2A	HGNC:12472
Ubiquitin-conjugating enzyme E2 B	UBE2B	HGNC:12473
Ubiquitin-conjugating enzyme E2 C	UBE2C	HGNC:15937
Ubiquitin-conjugating enzyme E2 D1	UBE2D1	HGNC:12474
Ubiquitin-conjugating enzyme E2 D2	UBE2D2	HGNC:12475
Ubiquitin-conjugating enzyme E2 D3	UBE2D3	HGNC:12476
Ubiquitin-conjugating enzyme E2 D4	UBE2D4	HGNC:2164
Ubiquitin-conjugating enzyme E2 E1	UBE2E1	HGNC:12477
Ubiquitin-conjugating enzyme E2 E2	UBE2E2	HGNC:12478
Ubiquitin-conjugating enzyme E2 E3	UBE2E3	HGNC:12479
Ubiquitin-conjugating enzyme E2 G1	UBE2G1	HGNC:12482
Ubiquitin-conjugating enzyme E2 G2	UBE2G2	HGNC:12483

Ubiquitin-conjugating enzyme E2 H	UBE2H	HGNC:12484
Ubiquitin-conjugating enzyme E2 J1	UBE2J1	HGNC:17598
Ubiquitin-conjugating enzyme E2 J2	UBE2J2	HGNC:19268
Ubiquitin-conjugating enzyme E2 K	UBE2K	HGNC:4914
Ubiquitin-conjugating enzyme E2 L3	UBE2L3	HGNC:12488
Ubiquitin/ISG15-conjugating enzyme E2 L6	UBE2L6	HGNC:12490
Ubiquitin-conjugating enzyme E2 N	UBE2N	HGNC:12492
(E3-independent) E2 ubiquitin-conjugating enzyme	UBE2O	HGNC:29554
Ubiquitin-conjugating enzyme E2 Q1	UBE2Q1	HGNC:15698
Ubiquitin-conjugating enzyme E2 Q2	UBE2Q2	HGNC:19248
Ubiquitin-conjugating enzyme E2 R2	UBE2R2	HGNC:19907
Ubiquitin-conjugating enzyme E2 S	UBE2S	HGNC:17895
Ubiquitin-conjugating enzyme E2 T	UBE2T	HGNC:25009
Ubiquitin-conjugating enzyme E2 U	UBE2U	HGNC:28559
Ubiquitin-conjugating enzyme E2 variant 1	UBE2V1	HGNC:12494
Ubiquitin-conjugating enzyme E2 variant 2	UBE2V2	HGNC:12495
Ubiquitin-conjugating enzyme E2 W	UBE2W	HGNC:25616
Ubiquitin-conjugating enzyme E2 Z	UBE2Z	HGNC:25847
Ubiquitin conjugation factor E4 A	UBE4A	HGNC:12499
Ubiquitin conjugation factor E4 B	UBE4B	HGNC:12500
Ubiquitin-like protein 4A	UBL4A	HGNC:12505
Ubiquilin-1	UBQLN1	HGNC:12508
UBX domain-containing protein 4	UBXN4	HGNC:14860
Ubiquitin-fold modifier-conjugating enzyme 1	UFC1	HGNC:26941
Ubiquitin recognition factor in ER-associated degradation protein 1	UFD1L	HGNC:12520
E3 UFM1-protein ligase 1	UFL1	HGNC:23039
Ubiquitin-fold modifier 1	UFM1	HGNC:20597
UDP-glucose:glycoprotein glucosyltransferase 1	UGGT1	HGNC:15663
UDP-glucose:glycoprotein glucosyltransferase 2	UGGT2	HGNC:15664
Ubiquitin carboxyl-terminal hydrolase 13	USP13	HGNC:12611
Ubiquitin carboxyl-terminal hydrolase 14	USP14	HGNC:12612
Ubiquitin carboxyl-terminal hydrolase 19	USP19	HGNC:12617
Ubiquitin carboxyl-terminal hydrolase 25	USP25	HGNC:12624
Vesicle-associated membrane protein-associated protein B/C	VAPB	HGNC:12649
Transitional endoplasmic reticulum ATPase	VCP	HGNC:12666
Deubiquitinating protein VCIP135	VCPIP1	HGNC:30897
Selenoprotein S	VIMP	HGNC:30396
Wolframin	WFS1	HGNC:12762
WD repeat domain phosphoinositide-interacting protein 1	WIPI1	HGNC:25471
X-box-binding protein 1	XBP1	HGNC:12801
Protein YIF1A	YIF1A	HGNC:16688

Ubiquitin thioesterase OTU1	YOD1	HGNC:25035
Zinc finger and BTB domain-containing protein 17	ZBTB17	HGNC:12936

Table S9. Differentially expressed proteostasis genes after treatment with effective PRs

Gene \ Treatment	MG132 [fold change]	BTZ [fold change]	CLC [fold change]	EerI [fold change]
ADRM1	1.97	2.12	1.94	1.97
AHSA1	2.19	1.89	n.c.	n.c.
CCL2	n.c.	1.59	1.52	1.72
CREB3L1	1.56	1.54	n.c.	n.c.
DDIT3	1.66	n.c.	n.c.	n.c.
DNAJA1	1.98	1.71	n.c.	n.c.
DNAJB1	5.12	3.05	n.c.	n.c.
ERO1LB	1.52	n.c.	n.c.	n.c.
FAF2	n.c.	1.51	n.c.	n.c.
FAM129A	2.54	2.44	2.00	2.08
FKBP7	1.54	n.c.	n.c.	n.c.
HSP90AA1	1.89	1.67	n.c.	n.c.
HSP90AB1	1.86	1.68	n.c.	n.c.
HSPA1A	5.67	4.83	n.c.	1.94
HSPA1B	12.59	9.42	n.c.	2.34
HSPB6	1.65	1.61	1.62	n.c.
HSPH1	2.79	2.16	n.c.	n.c.
JKAMP	1.74	1.79	n.c.	1.50
LMAN2L	1.61	n.c.	n.c.	n.c.
NGLY1	1.51	n.c.	n.c.	n.c.
NPLOC4	1.98	1.97	n.c.	1.56
NSFL1C	1.97	1.97	1.56	1.72
PSMA1	1.85	1.88	1.74	1.7
PSMA2	2.08	2.11	1.65	1.58
PSMA3	2.39	2.43	1.82	1.99
PSMA4	2.03	2.08	1.63	1.67
PSMA5	2.12	2.22	1.60	1.77
PSMA6	1.88	1.92	1.59	1.68
PSMA7	1.59	1.63	1.55	1.51
PSMB1	1.53	1.57	1.58	1.58
PSMB2	2.14	2.23	1.85	1.78
PSMB3	2.68	2.71	2.57	1.87
PSMB4	2.20	2.18	1.92	1.78
PSMB5	1.88	1.87	1.63	1.60
PSMB6	1.96	1.96	1.91	1.58
PSMB7	1.97	1.89	1.75	1.64
PSMC1	1.84	1.87	1.63	1.70
PSMC2	1.71	1.85	1.62	1.76
PSMC3	1.69	1.70	1.61	n.c.
PSMC4	3.04	3.17	2.43	2.08
PSMC5	2.05	2.06	1.78	1.64

**PSMC6**	2.38	2.37	1.53	1.91
**PSMD1**	2.02	2.04	1.72	1.70
**PSMD11**	2.45	2.63	1.93	2.05
**PSMD12**	2.25	2.33	n.c.	1.76
**PSMD13**	2.33	2.41	1.92	1.83
**PSMD14**	2.2	2.27	1.98	1.95
**PSMD2**	1.66	1.59	1.56	n.c.
**PSMD3**	2.38	2.55	1.99	2.04
**PSMD4**	1.88	1.86	1.54	n.c.
**PSMD6**	2.20	2.19	n.c.	1.58
**PSMD8**	1.72	1.71	1.57	n.c.
**STIP1**	2.25	1.97	n.c.	n.c.
**UBE2C**	1.67	n.c.	n.c.	1.75
**UBE2K**	1.57	1.61	n.c.	n.c.
**UBE2O**	1.84	1.83	n.c.	n.c.
**UBE2T**	1.54	n.c.	n.c.	1.59
**UBE4B**	1.53	1.52	n.c.	n.c.
**UBQLN1**	1.67	1.71	n.c.	n.c.
**UBXN4**	1.70	1.80	n.c.	n.c.
**UFD1L**	2.57	2.50	1.99	1.85
**USP14**	1.98	2.13	1.51	1.57
**VCP**	1.56	1.60	n.c.	n.c.
**WFS1**	2.51	2.35	1.77	n.c.

n.c.: not changed (differential expression < 1,5-fold or adjusted p-value ≤ 0.05)
red: upregulation; green: downregulation

Table S10. Differentially expressed lysosomal genes after treatment with effective PRs

Gene \ Treatment	MG132 [fold change]	BTZ [fold change]	CLC [fold change]	EerI [fold change]
**AP3S2**	1.53	1.52	n.c.	n.c.
**AP4B1**	1.64	1.76	n.c.	n.c.
**ARRB1**	1.80	1.53	1.51	n.c.
**ATP6V0A1**	1.85	1.68	n.c.	1.85
**ATP6V0B**	1.76	1.67	n.c.	1.67
**ATP6V0D2**	1.54	n.c.	n.c.	n.c.
**ATP6V1A**	2.24	2.19	1.59	1.66
**CLCN7**	1.52	n.c.	n.c.	n.c.
**CLU**	2.25	1.80	1.52	n.c.
**CTNS**	n.c.	n.c.	n.c.	1.54
**CTSC**	1.59	n.c.	n.c.	n.c.
**CTSL1**	n.c.	n.c.	n.c.	1.57
**DNAJC6**	2.93	2.97	1.54	1.71
**EGF**	2.15	2.12	1.90	n.c.
**ENPP1**	1.56	n.c.	1.74	1.92
**EPDR1**	1.52	n.c.	n.c.	n.c.
**GJA1**	1.59	n.c.	n.c.	n.c.
**GLA**	5.41	5.18	4.04	2.80
**GNB4**	1.89	1.71	n.c.	n.c.
**GNAQ**	1.51	1.53	n.c.	n.c.
**IFI30**	n.c.	n.c.	n.c.	1.54
**LDLR**	1.76	n.c.	n.c.	n.c.
**MAN2B1**	1.70	1.55	1.53	n.c.
**NPC1**	2.29	1.91	n.c.	1.80
**PLBD2**	1.76	1.53	1.66	n.c.
**RNF152**	1.65	1.66	n.c.	n.c.
**RPTOR**	1.55	n.c.	n.c.	n.c.
**SGSH**	1.69	1.53	n.c.	n.c.
**SLC48A1**	n.c.	1.55	n.c.	1.85
**SORT1**	1.66	n.c.	n.c.	n.c.
**TLR3**	1.52	n.c.	n.c.	n.c.
**TMEM97**	n.c.	n.c.	1.58	1.82
**USP5**	2.63	2.61	2.26	1.76

n.c.: not changed (differential expression < 1,5-fold or adjusted p-value ≤ 0.05)

red: upregulation; green: downregulation
